# Bone Research Society 2021 Abstracts

**DOI:** 10.1002/jbm4.10552

**Published:** 2021-10-26

## Overview of the BRS 2021 Online Annual Meeting

The Bone Research Society (BRS), formerly the Bone and Tooth Society, was founded in 1950. The BRS is one of the largest national scientific societies in Europe dedicated to clinical and basic research into mineralised tissues and is the oldest such society in the world. Meetings are held annually, attracting a wide audience from throughout the UK and beyond. The presentations are traditionally balanced between clinical and laboratory studies. The participation of young scientists and clinicians is actively encouraged.

Due to the unprecedented times which we had all experienced over the last year and like many other meetings, the BRS Annual Meeting 2021 was held online rather than in person, but that didn't stop our community continuing to produce high quality science and connecting with each other to exchange ideas and knowledge.

BRS 2021 was the platform for this, where the society ran a full programme of a webinars online over three days, with invited speakers, oral communications, virtual posters and satellite symposia from our Industry partners. In addition, three workshops were held in the evenings – the Rare Bone Disease workshop, Muscle and Bone workshop and the New Investigators workshop.

Online sessions were held over the course of three days from Monday 28 June to Wednesday 30 June 2021 covering:

 

• Rare Bone Disease

• Muscle and Bone

• Global musculoskeletal health

• Mineralisation cells and vesicles

• Applied mechanobiology

• Omics approaches to bone and muscle

• Using big data for research

• Animal models and bone

• Cancer and bone

• Kidneys and bone

 

***Organising Committee***

 

Dr Kassim Javaid

Dr Isabel Orriss

Prof. Elaine Dennison

Dr Claire Clarkin

Dr Dimitros Vlachopoulos

Dr Andrew Chantry

Prof. Kate Ward

Dr Michelle Lawson

Dr Laura Watts

Dr Ben Faber

Dr Alanna Green

Dr Sarah Allison

## Invited Speaker Abstracts

## Mineralomics and pathological calcification

### Sergio Bertazzo

#### *Department of Medical Physics & Biomedical Engineering University College London, London,* *UK*

**Abstract**

Physiological mineralization occurs naturally and is responsible for the formation and regeneration of hard tissues. On the other hand, mineralization of soft tissues, such as mineralization of the vasculature, has been associated to several diseases, including cardiac diseases, cancer and Alzheimer's disease. In this work we introduce the mineralomics (briefly, the characterization and study of minerals present in a biological context) and discuss how it may help us unveil key biological and medical information about cardiovascular diseases, breast cancer and Alzheimer's disease.

On cardiovascular diseases, if not the direct cause of death, calcification contributes considerably to complications that can lead to heart failure or heart attack. The origins and mechanisms of formation of vascular calcification are still strongly debated, with competing theories present in the literature. Almost a decade ago, we showed that nano‐sized and micron‐sized calcified spherical particles formed by a single crystal of magnesium‐containing calcium phosphate were the first calcified structure that could be detected in vascular tissue. Starting with the characterization of the mineral present on the vascular tissue and moving to the relationship between this mineral and the cells and proteins in the vascular system, we were able to propose a new mechanism for the origins of the first calcified structures in the vascular tissue.

Using the same principles of characterization of minerals, we also examined minerals found in breast tumors. Our results demonstrated the presence of nanosized and micron‐sized spherical particles made of highly crystalline whitlockite, which are exclusively found in the arterial wall of malignant invasive tumors.

More recently, we have also been working in the characterization of minerals present in the brains of Alzheimer's patients. Although mechanisms for the initial formation of minerals in the brain are not yet fully understood, the abnormalities mentioned in literature are directly related to a number of diseases, such as Fahr's disease, hypoparathyroidism, Alzheimer's disease, among others. Our characterization study shows the unique morphology, composition, crystallinity, and location of minerals in brain tissue samples from aged donors, Alzheimer's patients, and young donors. Moreover, we discovered that these minerals are related to the Tau protein, and are primarily located on nuclei of brain cells. The causes of this phenomenon are still elusive and are being investigated by my group, since they could have an important role on charting brain aging and further implications for the study of mental disorders such as Alzheimer's.

Seen as a whole, our work demonstrates the potential of the mineralomics field and shows that mineralomics enables the study of pathological minerals, which have long held and undervalued power to predict disease onset and provide valuable information about diseases and their mechanisms. We believe that mineralomics has the potential to provide critical insights into human (patho)biology and to contribute to breakthroughs in medicine, especially when integrated into a multiomics research approach.

## Insights and challenges in the management of children with achondroplasia

### Moira S. Cheung

#### *Evelina London Children*'*s Hospital, London, UK*

**Abstract**

Achondroplasia is the most common form of skeletal dysplasia and caused by a variant in the fibroblast growth factor receptor 3 (FGFR3).

Previously, the majority of children with achondroplasia were managed locally as there were no perceived treatment options. The natural history and complications, therefore, were poorly described. Advances in drug trials and potential treatment options has helped to centralize the management and larger cohorts of patients.

Greater expertise and understanding of the natural history of achondroplasia in children has emerged. Along with this the management of children with achondroplasia has also rapidly progressed.

This talk will give a brief outline of the medical complications that affect children with achondroplasia and some of the management strategies that have been developed. The talk will focus on the newborn period and the challenges of foramen magnum stenosis and also cover areas which remain challenging and present as future areas of research.

## Global differences in fracture epidemiology

### Cyrus Cooper, OBE

#### Lifecourse Epidemiology Unit, University of Southampton; and Institute of Musculoskeletal Sciences, University of Oxford, Southampton/Oxford, UK

**Abstract**

Osteoporosis constitutes a major public health problem through its association with age related fractures.

These fractures typically occur at the hip, spine, and distal forearm. It has been estimated from incidence rates derived in North America that the lifetime risk of a hip fracture in Caucasian women is 17.5%, with a comparable risk in men of 6%. Age and sex‐adjusted hip fracture rates are generally higher in Caucasian than in Asian populations.

Furthermore, the pronounced female preponderance in fracture incidence observed in white populations is not seen among blacks or Asians in whom age‐adjusted female to male incidence ratios approximate unity. Life expectancy is increasing around the globe and the number of elderly individuals is rising in every geographic region. Assuming constant age‐specific incidence rates for fracture, the number of hip fractures occurring worldwide among people aged 65 years and over will rise from 1.66 million in 1990 to 6.26 million in 2050.

Studies performed in the United States, Scandinavia, and the United Kingdom, between 1930 and the late 1980s, consistently reported increases in the age‐adjusted incidence of hip fractures among men and women. This increase appears to have leveled off, in the northern regions of the United States, as well as more recently in Europe. Rates in Asian populations continue to show substantial rises between the 1960s and the present time.

In the most recent data available from the United States, the incidence of first ever hip fracture declined by 1.37% per year among women and 0.06% per year among men. The cumulative incidence of a second hip fracture after 10 years was 11% among women and 6% among men, when death was treated as a competing risk. The reduction in hip fracture occurrence was even greater than that expected from the declining incidence of hip fractures more generally. Age‐period‐cohort models have suggested influences of all three contributors to these secular trends. Among current risk factors for low bone density and trauma (low body mass index, cigarette smoking, alcohol consumption, physical inactivity, and dietary calcium intake) the trends are best explained by physical inactivity. Developmental contributors to peak bone and muscle mass, for example maternal nutrition and lifestyle, also appear capable of contributing to cohort effects.

Finally, debate continues on the role of more aggressive osteoporosis risk assessment and therapeutic strategies in contributing to the secular decline in hip fracture rates generally. Although pharmacologic intervention might be efficacious, only a minority of hip fracture patients remain so treated, and the scope for even greater reductions in incidence remains an enticing prospect.

## Characterization of patterns of disuse‐related osteoporosis

### Sylvie Coupaud

#### Department of Biomedical Engineering at the University of Strathclyde, Glasgow, UK

**Abstract**

The rapid deconditioning of body systems following spinal cord injury (SCI) has been likened to accelerated aging. Specifically, in the musculoskeletal system, disuse‐related bone loss follows muscle atrophy, and is manifested as a decrease in bone mineral density (of up to 50% in the first year post‐SCI) and a thinning of the cortical shaft, rendering the bones more susceptible to fracture from everyday activities. Osteoporotic fractures are common in chronic SCI, especially around the lower limb joints.

Coupaud and her team at the University of Strathclyde, alongside clinical collaborators at the Queen Elizabeth National Spinal Injuries Unit (Glasgow, UK), perform longitudinal, cross‐sectional and interventional patient studies, using multiscale imaging and modeling to characterize bone loss after SCI. Individualized finite‐element models of the long bones provide predictions of the osteoporotic bones' susceptibility to fracture in simulated loading scenarios.

Animal models are used to investigate factors affecting spatial and temporal patterns of bone loss associated with paralysis, but in the absence of the confounding co‐morbidities affecting patients. A rodent model of SCI is providing a comprehensive characterization of changes in the microarchitecture and mechanical properties of cancellous and cortical bone compartments of the long bones, to quantify their effects on fracture susceptibility.

## Forty years of research into bone turnover markers—Dent Lecture

### Richard Eastell

#### Department of Oncology and Metabolism, University of Sheffield, Sheffield, UK

**Abstract**

I started my research into bone metabolism around 40 years ago and at that time we could assess bone resorption using urinary hydroxyproline and the fasting urine calcium to creatinine ratio and bone formation by measuring total alkaline phosphatase activity. None of these markers were specific to bone and as a result they were not suitable for studying small changes in bone turnover that we see in osteoporosis.

The first bone‐specific marker for bone formation was osteocalcin and this was introduced early in the 1980s and the following 15 years were the golden age for bone turnover markers with HPLC assays for pyridinium crosslinks of type I collagen and then immunoassays for collagen‐based formation markers such as procollagen I N‐ and C‐propeptides and for resorption markers such as the N‐ and C‐telopeptides of type collagen, free deoxypyridinoline, and tartrate resistant acid phosphatase type 5B. In the setting of osteoporosis, the most sensitive markers appear to be the C‐telopeptide of type I collagen for bone resorption and the N‐propeptide of type I collagen for bone formation.

These markers are endorsed by the International Osteoporosis Foundation and have proven useful in drug development for identifying the best dose and treatment regimen and in clinical practice for monitoring osteoporosis treatment.

## Management of achondroplasia in adults

### Svein O. Fredwall

#### TRS National Resource Centre for Rare Disorders, Sunnaas Rehabilitation Hospital, Nesoddtangen, Norway

**Abstract**

Achondroplasia is the most common disproportionate form of skeletal dysplasia, affecting more than 250,000 individuals worldwide. Achondroplasia has been known for centuries, yet the existing literature on natural history, including medical complications, physical functioning, and psychosocial health in adults with this condition is sparse. Although several clinical guidelines and recommendations have been developed for the management and care of children with achondroplasia, no such guidelines exists for the management of adults.

This presentation will provide recommendations for the clinical practice management of adults with achondroplasia. The recommendations are based on: current literature evidence; clinical experience and research evidence obtained from The Norwegian Adult Achondroplasia Study; and the recently developed international consensus‐based statements for management and care of achondroplasia.

Key topics will include neurological complications, in particular symptomatic spinal stenosis, orthopedic complications, physical functioning, pain, obstructive sleep apnea, cardiovascular risk factors, obesity, hearing, and psychosocial issues. The presentation will also discuss some general principles for the optimal management and care of adults with achondroplasia, and outline remaining research gaps.

## The joys of being an accidental academic—Dent Lecture

### Miep Helfrich

#### University of Aberdeen, Aberdeen, UK

**Abstract**

In this Dent lecture I will look back and reflect on what made my work as researcher and then as academic enjoyable. Of course, this includes interesting research findings, such as discovering the ligands of osteoclast integrins, or culturing osteoclasts from patients with osteopetrosis or Paget's disease to study their defects, and working up new methods for ultrastructural imaging of bone cells. But just as important were the collaborations with other researchers, the teaching and training of students and postdoctoral researchers, the mentoring of colleagues, speaking to the general public about science, and being an active member of scientific societies.

Being an academic includes contributing to the running of the Institution where you work. I enjoyed getting involved in committees that help shape the working environment and thereby understanding more about the role of the many departments and staff in them that make it possible for academics to do what we do. OK, I admit, it was not always all rosy, and I may mention a frustration or two in passing, but the focus will remain firmly on what I found the positive and enjoyable aspects.

## Neuromuscular factors influencing muscle force

### Tom Maden‐Wilkinson

#### Advanced Wellbeing Research Centre, Sheffield Hallam University, Sheffield, UK

**Abstract**

This talk will discuss the both the neural and muscular factors which underpin maximal force production, the relative importance of these factors in maximizing how quickly force can be produced (rate of force development) and how this relates to functional performance in humans. Following this, the talk will have a particular emphasis on the neural and muscular adaptations to strength training and the time course and relative importance of them for increasing maximal strength.

**References**

1. Maden‐Wilkinson T, Balshaw T, Massey G, Folland J. Muscle architecture and morphology as determinants of explosive strength. *Eur J Appl Physiol*. 2021;121, 1099‐1110. http://doi.org/10.1007/s00421-020-04585-1

2. Balshaw TG, Maden‐Wilkinson T, Massey GJ, Folland JP. The human muscle size and strength relationship. Effects of architecture, muscle force and measurement location. *Med Sci Sports Exerc*. 2021;53(10):2140‐2151. http://doi.org/10.1249/mss.0000000000002691

3. Maden‐Wilkinson TM, Balshaw TG, Massey G, Folland JP. What makes long‐term resistance‐trained individuals so strong? A comparison of skeletal muscle morphology, architecture, and joint mechanics. *J Appl Physiol*. 2020;128(4):1000‐1011. http://doi.org/10.1152/japplphysiol.00224.2019

4. Balshaw TG, Massey GJ, Maden‐Wilkinson T, Lanza MB, Folland JP. (2018). Neural adaptations after 4 years vs. 12 weeks of resistance training vs. untrained. *Scand J Med Sci Sports*. 2018;29(3):348‐359. http://doi.org/10.1111/sms.13331

5. Balshaw TG, Massey GJ, Maden‐Wilkinson T, et al. Changes in agonist neural drive, hypertrophy and pre‐training strength all contribute to the individual strength gains after resistance training. *Eur J Appl Physiol*. 2017;117(4):631‐640. http://doi.org/10.1007/s00421-017-3560-x

6. Balshaw TG, Massey GJ, Maden‐Wilkinson T, Tillin NA, Folland JP. Training‐specific functional, neural, and hypertrophic adaptations to explosive‐ vs. sustained‐contraction strength training. *J Appl Physiol*. 2016;120(11):1364‐1373. http://doi.org/10.1152/japplphysiol.00091.2016

## Animal models of bone mineralization and vascular calcification

### José Luis Millán

#### *Sanford Children's Health Research Center, Sanford Burnham Prebys Medical Discovery Institute, La Jolla,* *CA* *,* *USA*

**Abstract**

Work in my laboratory focuses on understanding the mechanisms of initiation of skeletal and dental mineralization using the power of mouse genetics. These studies have helped clarify the pathophysiology of heritable forms of rickets/osteomalacia (hypophosphatasia or HPP and PHOSPHO1 deficiency) and also ectopic calcification disorders, involving the vascular intima and media.

In this talk, I will describe the phenotypes of murine models of *Alpl* deficiency, that via ablating or reducing the expression of tissue‐nonspecific alkaline phosphatase (TNAP), phenocopy early‐onset and late‐onset HPP and refer to our past and current preclinical efforts to treat this disease.

I will also refer to the PHOSPHO1 deficiency as a form of Pseudo‐HPP, and the *Alpl/Phospho1* double knockout mice that have given us unique insights into the role of these enzymes in matrix vesicles‐mediated mineralization. Upregulation of TNAP in the vascular media leads to severe vascular calcification that phenocopies generalized arterial calcification of infancy, while upregulating TNAP in the intima in atherogenic mouse models phenocopies features of atherosclerosis.

In collaboration with medicinal chemist at our Institute we have developed first‐in‐class TNAP inhibitors able to mitigate the severity of ectopic calcification in mouse models of rare diseases, chronic kidney disease, and atherosclerosis.

## Nutritional rickets in the modern times—Dent Lecture

### Zulf Mughal

#### *Department of Pediatric Endocrinology Royal Manchester Children's Hospital, Manchester University* *NHS* *Foundation Trust, Manchester, Ukraine*

**Abstract**

Nutritional rickets (NR) is a disorder of the growing child arising from impaired mineralization of the growth plate and osteoid, secondary to vitamin D and/or dietary calcium deficiency.

In the 19th century, there was a marked increase in the prevalence of NR in England as it was the first country to embrace the industrial revolution, which was accompanied by the mass movement of people from rural communities to dark, heavily polluted, and overcrowded cities. The misery of English children growing up in such appalling slums is vividly described in the novels by Charles Dickens. Hence, NR became known as the “English disease.” Series of nutritional experiments and studies during the early 20th century confirmed that ultraviolet light caused the synthesis of a fat‐soluble substance in the skin that we now know as vitamin D. Thus, it became apparent that exposure to sunlight or vitamin D supplementation (either as cod liver oil or synthetic vitamin D) was able to cure NR. Improvements in infant nutrition and free distribution of cod liver oil/vitamin D supplements to children in child welfare clinics during the 1940s led to the virtual eradication of NR in the UK. In the post–Second World War period, the shortage of labor in factories in the UK led to the migration of workers from the West Indies, India, & Pakistan (which at the time also incorporated Bangladesh). From the 1960s onward, NR started emerging as an important health problem among children of immigrants.

My interest in this condition started in the 1990s when I started seeing children with NR during my Pediatric training. In my lecture, I shall describe how clinical cases, along with studies in the UK, India and Afghanistan have helped my understanding of the etiology, risk factors, treatment, and prevention of NR.

## How big data answers big questions well and not so well

### Jon H. Tobias

#### Musculoskeletal Research Unit, Translational Health Sciences, University of Bristol, Bristol, UK

**Abstract**

Recent years have seen an exponential rise in size of datasets for osteoporosis and osteoarthritis research. This partly reflects the increasing number of individuals comprising study cohorts, exemplified by UK Biobank with around 500,000 participants. In addition, high dimensional data is increasingly used, such as molecular genetic data, and phenotypic data obtained from complex imaging modalities like MRI and CT. For example, in genome‐wide association studies (GWAS), over 25 million single nucleotide polymorphisms (SNPs) across the genome, imputed from SNPs genotyped on arrays, are analyzed in relation to quantitative traits like bone mineral density (BMD), or to case status such as a history of fracture or osteoarthritis.

Several studies have utilized GWAS data to identify new therapeutic targets for osteoporosis and osteoarthritis. Disease‐associated loci can be matched to genes that are the target of drugs already in use or in development, and online resources can be used to interrogate other promising candidates. A difficulty is to establish whether the locus identified is causally related to the disease under study, rather than, for example, being in linkage disequilibrium with a distinct locus that is itself responsible for the disease. Colocalization studies using eQTL data are used to address this question, by examining whether genetic association with the trait colocalizes with mRNA expression. Colocalization with protein can also be evaluated, based on circulating biomarkers, which would have accurately predicted successes of the RANKL antibody, denosumab, and sclerostin antibody, romosozumab, in treating osteoporosis. Big data can also be harnessed to identify new therapeutic targets by studying rare bone diseases. The most successful example was identification of a *SOST* mutation underlying the high bone mass disorder (HBM) sclerosteosis, ultimately leading to the development of the romosozumab. Having established the HBM cohort from individuals undergoing DXA scanning in the UK, we subsequently developed and applied a whole exome sequencing pipeline, identifying *SMAD9*, *GALNT3*, and *PIEZO1* as additional genes related to HBM, which might also represent useful therapeutic targets.

GWAS findings can be leveraged in Mendelian RANDOMIZATION (MR) studies intended to examine causal relationships with risk factors. For example, in a MR study of fracture risk, although grip strength was found to have a causal, protective effect on fracture risk, no effect was seen for vitamin D levels. MR has also been applied to interrogate adverse effects of drug therapies. For example, whereas concerns have been raised over possible increased cardiovascular risk following romosozumab, SNPs associated with reduced *SOST* expression were recently found to be unrelated to cardiovascular endpoints. MR studies are subject to a number of assumptions; however, newer analysis techniques enable these to be tested and controlled for. GWAS findings can also be used to generate polygenic risk scores, which can be used to predict disease occurrence. However, to date, the additional predictive value of polygenic risk scores for fracture risk is somewhat low when combined with conventional factors, particularly BMD. High‐dimensional imaging data may also enable improved accuracy of disease prediction, as illustrated by recent findings that hip shape modes, derived from principle components analysis of hip DXA scans, are predictive of total hip replacement in UK Biobank.

## Oral Communications Abstracts

### Animal Models

## Disuse‐related cortical bone loss exhibits delayed onset yet stabilizes more rapidly than trabecular bone

### Samuel Monzem ^1,2^, Behzad Javaheri^1^, Roberto de Souza^2^, Andrew Pitsillides^1^

#### ^*1*^ *Royal* *Veterinary College, London, UK.* ^*2*^ *Veterinary* *College ‐* *UFMT* *, Cuiaba, Brazil*

**Abstract**

Disuse osteoporosis occurs after extended periods of bed rest or nerve damage leading to increased risk of fracture. It remains to be established, however, whether the trajectory of bone loss is equivalent in bone's cortical and trabecular compartments following long‐term disuse. We aim to use μCT to evaluate whether trabecular and cortical bone compartments behave similarly in response to defined periods of disuse. The right hind‐limb of seventeen 12‐week‐old female mice was subjected to sciatic neurectomy (SN, left limb served as control) and euthanized in four groups (*n* = 3–5/group) at 5, 35, 65, and 95 days. Trabecular bone was examined at the proximal metaphysis and cortical bone mass and geometry were evaluated along almost the entire tibia length. We found trabecular bone volume/total volume, number, and mineral density rapidly decreased within the first 5 days and exhibited a trajectory of loss that plateaued only after 65 days post‐SN. In contrast, decreased cortical cross‐sectional area (CSA, and others measures of mass/geometry) along the tibia length reached levels of significance (*p* < 0.001) only much later, at 35 days, and surprisingly no further deterioration was observed thereafter. These data indicate that disuse‐related cortical bone loss exhibits delayed onset yet stabilizes more rapidly than trabecular bone (Fig. 1). These data suggest that trabecular and cortical bone compartments behave as distinct modules in response to disuse.

**Figure:**
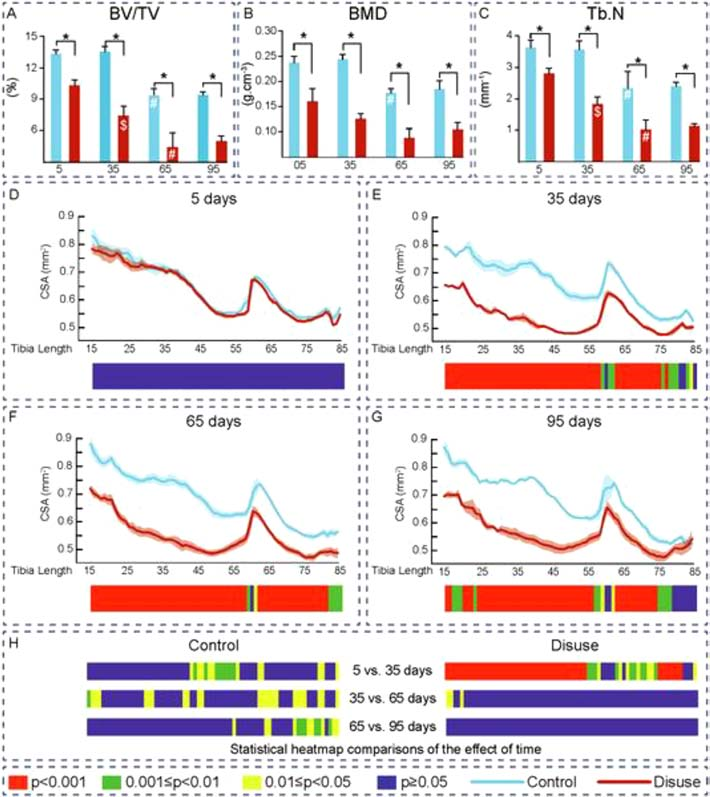
**Fig. 1.** Mean ± SEM of A: Bone volume/Trabecular volume (BV/TV), B: Bone Mineral Density (BMD); C: Trabecular Number (Tb.N); Cross sectional area (CSA) and statistical heatmap after: D: 5 days, E: 35 days, F: 65 days and G: 95 days of disuse. H: Levels of significance along the time of 12 week‐old‐mice; left tibia (Control) and right tibia (Disuse‐ by Sciatic Neurectomy) after 5, 35, 65 and 95 days of disuse. $: p < 0.05 when compare 5 vs 35 days inside same group. #: p < 0.05 when compare 35 vs 65 days inside same group.

## Do 3D epiphyseal bone architectural changes in ageing STR/Ort and healthy mice reveal early imaging biomarkers of osteoarthritis?

### Lucinda Evans ^1^, Eva Herbst^2^, Alessandro Felder^3^, Sara Ajami^4^, Behzad Javaheri^5^, Andrew Pitsillides^1^

#### ^*1*^ *Skeletal* *Biology Group, Royal Veterinary College, London, UK.* ^*2*^ *Paleontological* *Institute and Museum, University of Zurich, Zurich, Switzerland.* ^*3*^ *Research* *Software Development Group, University College London, London, UK.* ^*4*^ *Great* *Ormond Street Institute of Child Health, University College London, London, UK.* ^*5*^ *School* *of Mathematics, Computer Science and Engineering, City University of London, London, UK*

**Abstract**

Novel imaging biomarkers are required to advance research into, and treatment of, knee joint osteoarthritis (OA). Currently, early‐stage OA is undetectable in humans, and treatment effectiveness cannot be reliably monitored. STR/Ort mice are an age‐related model of progressive OA, in which predisposition (at 10 weeks of age), early‐stage onset (at 20 weeks), and late‐stage OA (at 40 weeks) are well‐defined. Using STR/Ort (OA) and CBA (healthy parental control) mice at these age intervals, knee joints were non‐invasively μCT‐imaged with effective pixel size 5 μm. Tibial epiphyses were semi‐automatically segmented from joints and separated into their constituent anatomical components: cortical bone, trabecular bone, and marrow space volume (Fig. 1*A*–*D*). Crucially, these bony features can be detected in human knees using clinical in vivo scanners (XtremeCT II HR‐pQCT, Scanco Medical) making translation of our research realistically attainable in the near‐future.

3D analyses of tibial epiphyses followed by two‐way ANOVA confirmed significant age‐ and/or strain‐related differences in epiphyseal cortical bone volume (*p* ≤ 0.001), trabecular bone volume (*p* ≤ 0.001), mean trabecular and cortical bone thicknesses (both *p* values ≤ 0.010), trabecular volume relative to cortical volume (*p* ≤ 0.001), and degree of anisotropy, a descriptive measurement of trabecular orientation (*p* = 0.001, Fig. 1*E*). The two mouse strains had different epiphyseal growth patterns throughout life with respect to total epiphyseal volume (*p* = 0.023), marrow space volume (*p* = 0.002), and trabecular volume relative to epiphyseal interior volume (*p* = 0.024), as well as changes in trabecular anisotropy indicating divergent and potentially exciting age‐related interactions in these OA‐prone and healthy mouse strains.

Our findings disclose new imaging biomarkers of pre‐OA, OA onset, and OA progression in the STR/Ort mouse, an established animal model of spontaneous age‐related OA. Due to our exploitation of only gross‐anatomical features in the tibial epiphysis, which can also be segmented from clinical CT scans, we anticipate future translation of this promising research into human clinical practice.

**Figure:**
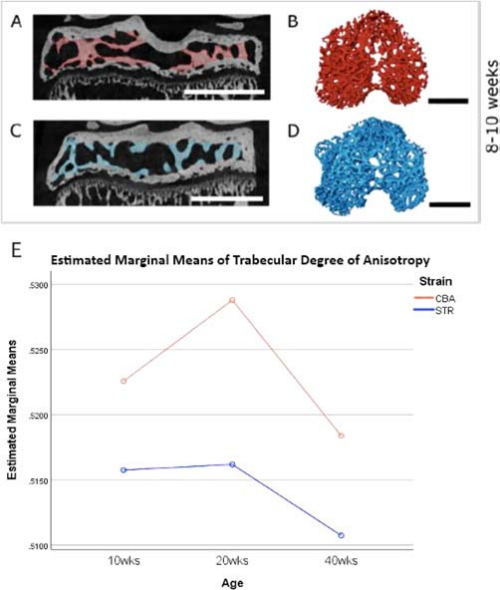
**Figure 1:** Semi‐automated segmentation and analysis of mouse tibial epiphyses. Red = CBA (healthy) trabeculae, blue = STR/Ort (OA) trabeculae. A & C: Central slices from reconstructed μCT scans viewed in coronal plane, with segmented trabeculae highlighted in colour. B & D: Corresponding trabeculae as rendered as a 3D volume, viewed from above. A‐B: 8‐10wk CBA mouse; C‐D: 8‐10wk STR/Ort mouse. Scale bars = 1mm. E: Estimated marginal means plot depicting significant trabecular anisotropy differences between STR/Ort and CBA mice (p = 0.001) at landmark ages.

## The novel high bone mass–associated Smad9 transcription factor is predominantly expressed in osteochondral progenitor populations of zebrafish skeletal elements

### Georgina McDonald , Mengdi Wang, Chrissy Hammond, Dylan Bergen

#### University of Bristol, Bristol, UK

**Abstract**

**Background:** Osteoporosis is a prevalent age‐related disease characterized by low bone mineral density and fragility fractures. In the clinic there is a demand for new osteoanabolic treatments to strengthen bones. A powerful way to identify new osteoanabolic targets is by understanding the genetics of high bone mass (HBM). Recently, a damaging p.Leu22Pro mutation in *SMAD9* was identified in a HBM pedigree, which was independently confirmed in two unrelated HBM cases. SMAD9 is an inhibiting transcription factor in the bone morphogenetic protein (BMP) signaling pathway, whereas SMAD1/5 activate transcription. Zebrafish are a suitable model as they have similar skeletal physiology to humans and can be easily pharmacologically manipulated and imaged.

**Methods:** We used zebrafish transgenic reporters to visualize osteoblasts (*sp7:GFP*) and the BMP‐pathway (*BMPre:GFP*). Protein expression was visualized with α‐Smad9, α‐GFP and α‐collagen2a1 antibodies. Three day‐old larvae were treated with 0.01% DMSO, 4μM dorsomorphin (DM), 1μM retinoic acid (RA), or 25μM prednisolone (PD) for 2 days. Six‐hour nifurpirinol (5μM) treatment was used to ablate osteoblasts in the sp7*:mCherry‐NTR* transgenic. Adult caudal fins were amputated or fractured and assessed 7 days later. Experiments were ethically reviewed and performed under UK Home Office license (30/3801).

**Results:** This research shows that Smad9 is expressed by a group of osteochondral precursor cells in the developing skeleton, which do not express Smad1/5. Specifically, Smad9 is expressed within cartilage‐bone interfaces and joints during early craniofacial development. Smad9^+^ cells are proliferative and expand during osteoblast regeneration after chemically induced ablation. DM, RA, and PD are drugs known to manipulate skeletal cell fate. RA caused a loss of Smad9 expression and inhibited osteoblast regeneration. PD reduced Smad9 expression at bone and cartilage sites whereas DM had no effect. Smad9 expression changes when joint morphology is altered in mutants and by immobilization. In caudal dermal bone fin regeneration and fracture repair, we observed elevated Smad9 expression in osteoblasts that make newly formed bone.

**Conclusions:** We show that Smad9 is a marker for an undifferentiated osteochondral precursor population in both larval and adult skeletal tissues. This population can be pharmacologically manipulated and responded to osteoblast ablation in larvae. In adult dermal bone of the caudal fin Smad9 expression is unregulated in response to fracture repair and bone regeneration. Because BMP‐inhibitor DM did not show an effect, we showed that Smad9 is likely a repressor R‐Smad in vivo. These findings make Smad9 an attractive candidate for an osteoanabolic drug target.

## Repairing myeloma bone disease; can the combination of bone anabolic and antiresorptive therapy improve bone quality and repair?

### Becky Andrews ^1,2^, Alanna Green^1^, Holly Evans^1^, Jenny Down^1^, Darren Lath^1^, Janet Brown^1,2^, Michelle Lawson^1^, Andy Chantry^2,1^

#### ^*1*^ *University* *of Sheffield, Sheffield, UK.* ^*2*^ *Sheffield* *Teaching Hospitals,* *NHS* *Foundation Trust, Sheffield, UK*

**Abstract**

**Background:** Approximately 90% of patients diagnosed with multiple myeloma (MM) will suffer from myeloma‐induced bone disease (MBD). MBD causes increased fracture risk, poor mobility and chronic pain for patients. Antiresorptives (eg, zoledronic acid [ZA]) are standard of care, but there is increasing interest in the clinical use of bone anabolics to attempt to repair bone. Inhibitors of TGF‐β have shown bone anabolic effects and MBD repair in preclinical studies, but these have not been compared to, or combined with, antiresorptive treatments in a chemotherapy‐treated model.

**Aims:** We hypothesized that combination of antiresorptive and anabolic therapies would repair MBD more effectively than either monotherapy.

**Methods:** NSG mice (*n* = 8/group) were inoculated via tail vein injection with 1 × 10^6^ U266‐GFP‐Luc human myeloma cells. Tumor was monitored by bioluminescence imaging (BLI) and MBD by in vivo μCT imaging. When MBD was established, mice were randomized into five treatment groups (*n* = 8/group): (i) vehicle, (ii) dual chemotherapy (Bortezomib [B] and Lenalidomide [L]), (iii) B/L and SD208 (a TGF‐β receptor‐I kinase inhibitor), (iv) B/L and ZA, and (v) B/L/ZA/SD208, receiving treatment for 4 weeks before euthanasia.

**Results:** Tumor reduced rapidly in the first week of treatment in all B/L treatment groups compared to vehicle as demonstrated with BLI (*p* < 0.01); ex‐vivo flow cytometry (*p* < 0.01) and ex‐vivo serum IgE (*p* < 0.01). Total bone volume (TBV) was analyzed as percentage change from baseline, with increases demonstrated with B/L as early as week 2. At week 4, percentage increase in TBV was 48% for B/L (*p* < 0.01), 58% for B/L/SD208 (*p* < 0.01), 117% for B/L/ZA (*p* < 0.01), and 129% for B/L/ZA/SD208 (*p* < 0.01). Significant osteolytic lesion repair was observed with B/L/SD208, B/L/ZA, and B/L/SD208/ZA treatment by week 2 (week 3 for B/L). Increased trabecular BV was observed in B/L/SD208, B/L/ZA, and B/L/ZA/SD208 treated groups compared to vehicle. Raman spectroscopy performed on cortical bone suggested mineralization, carbonate substitution, and matrix maturity in treatment groups is comparable to that of naïve, age‐matched mice. Biomechanical testing (femur three‐point bending) demonstrated recovery of bone stiffness in all treatment groups, with analysis ongoing to assess fracture resistance.

**Conclusions:** SD208 elicited no tumorigenic effects. Combined treatment demonstrated the most significant increase in TBV. Bone composition appeared comparable to naïve mice in all treatment groups, suggesting healthy composition of repaired bone. Analysis is ongoing to assess changes in the bone microenvironment, and determine possible therapeutic benefit to bone quality and repair with combination therapy.

## Transcriptomic profiling of regenerating zebrafish scales identifies osteogenic genes that are involved in human bone disease

### Dylan Bergen ^1^, Qiao Tong^1^, Ankit Shukla^2^, Elis Newham^1^, Jan Zethof^3^, Mischa Lundberg^2^, Rebecca Ryan^1^, Scott Youlten^4^, Erika Kague^1^, Eleftheria Zeggini^5^, Peter Croucher^4^, Gert Flik^3^, Rebecca Richardson^1^, John Kemp^2^, Chrissy Hammond^1^, Juriaan Metz^3^

#### ^*1*^ *University* *of Bristol, Bristol, UK.* ^*2*^ *University* *of Queensland, Brisbane, Australia.* ^*3*^ *Radboud* *University, Nijmegen, Netherlands.* ^*4*^ *Garvan* *Institute, Sydney, Australia.* ^*5*^ *Institute* *of Translational Genomics, Munich, Germany*

**Abstract**

**Background:** Exoskeletal structures were the earliest elements to gain biomineralization capacities in the vertebrate common ancestor. During evolution, this process is thought to have functioned as a template for other structures that can mineralize, including endoskeletal elements. However, terrestrial vertebrate exoskeletal structures have been mostly lost during evolution. Most bony fish have retained exoskeletal structures in the form of scales. Scales are mineralized plates that can completely regenerate involving de novo matrix mineralization. Each scale is an independent mineralized unit, and therefore this regenerative capacity offers a potential route to identify new potential osteoanabolic factors as they both have mineralized matrix resorbing and building cells. However, it is long thought that zebrafish scales were more odontogenic rather than osteogenic in their formation processes.

**Results:** To address this, we defined the transcriptomic profiles of ontogenetic and regenerating scales of zebrafish. We identified 604 differentially expressed genes (DEGs) that were enriched for extracellular matrix, ossification, and cell adhesion pathways but not in enamel or tooth formation processes. To further determine whether DEGs resembled genes involved in bone formation, we deployed gene set enrichment analyses of DEG human orthologous on monogenic and polygenic association studies. Indeed, we found that human orthologues of DEGs were 2.8 times more likely to cause human monogenic skeletal diseases (*p* < 8 × 10^−11^), and they showed enrichment for human orthologues associated with polygenetic disease traits (UK‐Biobank) including stature, bone density and osteoarthritis (*p* < 0.005). To determine whether zebrafish scales DEGs and their association profiles were evolutionary conserved, zebrafish mutants of two human orthologues that were robustly associated with height and osteoarthritis (*COL11A2*) or estimated bone mineral density only (*SPP1*) were phenotypically assessed for both exoskeletal and endoskeletal abnormalities. Consistent with our genetic association studies, *col11a2* ^*Y228X/Y228X*^ mutants showed exoskeletal and endoskeletal features consistent with abnormal growth and osteoarthritis. The *spp1* ^*P160X/P160X*^ mutants showed mineralization defects in regenerating scales whereas endoskeletal elements depicted elevated eBMD (*p* < 0.05).

**Conclusions:** We show that scales have a stronger osteogenic expression profile than previously thought, Despite the fact that there are many differences between scale and endoskeletal developmental processes, our data highlight a relation with bone diseases. Understanding scale regeneration processes beyond their physiological context could therefore reveal novel evolutionarily conserved osteoanabolic processes. Thus, regenerating scales offer an attractive alternative of identifying new genes and pathways involved in bone formation that resemble a sub‐population of genes that are relevant to human skeletal disease.

## Investigating skeletal architecture in the mdx mouse

### Mark Hopkinson, Dominic Wells, Andrew Pitsillides

#### Royal Veterinary College, London, UK

**Abstract**

Duchenne muscular dystrophy (DMD), caused by dystrophin gene mutations, elicits poor bone health; DMD patients have short stature, low BMD, osteomalacia, osteopenia, and increased fracture risk. These skeletal characteristics have historically been attributed solely to muscle weakness and correspondingly reduced bone loading. There are, however, studies indicating that dystrophin may have direct roles in skeletal tissues. Most have reported skeletal defects, after the onset of muscle damage, in either mdx mice or human DMD patients. Few, importantly, have focused on whether bone changes may occur sooner, before the onset of muscle damage, and shown similar reduction in cortical and trabecular bone volume and impaired collagen organization culminating in weaker bones in mdx mice. Interrogation of these data reveals that their histological and μCT based phenotyping was however limited only to selected bone regions, highlighting that microarchitectural bone changes in entire mdx bones remained unresolved. My studies address these shortcomings by undertaking an analysis of entire tibia and femur of 3‐week‐old male mdx (versus wild‐type [WT], *n* = 6). by μCT.

Analyses revealed that mdx mice exhibit elevated tibial cortical bone volume (*p* < 0.05) and a divergent shape (versus WT); rounder cross‐sectional profile close to the tibiofibular junction (*p* < 0.01). Greatest differences were however evident in tibial trabecular regions, where mdx mice exhibited a *less mature* organization with more numerous, thinner, less separated, highly connected trabeculae (*p* < 0.001). This was somewhat at odds with the interesting observation that mdx mouse tibiae were also longer (versus WT) (*p* < 0.05). Analysis of the femur reinforced the findings of greater cortical bone area, which was significantly thicker at the midshaft and distally (*p* < 0.05). Intriguingly, mdx femoral shape divergence was also aligned with those in tibias, where specific regions also comprised significantly rounder cross‐sectional profiles (*p* < 0.01) and greater predicted indices of bending strength (between 30% and 50% of the proximodistal length) (*p* < 0.05). Akin to the tibia, the femoral trabecular bone in mdx mice comprised more numerous, thinner, less separated, and less connected trabeculae and, in addition, occupied a significantly smaller medullary area (*p* < 0.001). Tissue mineral density of cortical bone was also significantly lower in mdx mice, most notably around the knee joint in the distal femur and proximal tibia (*p* < 0.05).

Overall, this study revealed that mdx mice possess tibial and femoral microarchitecture differences when compared to WT mice prior to the onset of muscle damage, consistent with the hypothesis that dystrophin serves direct roles in skeletal tissues.

## Are epiphyseal growth plates sensitive to short‐term load‐induced mechanical stimuli*?*

### Dionysia Valkani, Behzad Javaheri, Andrew A Pitsillides

#### Royal Veterinary College, London, UK

**Abstract**

Endochondral ossification (EO), critical during skeletal growth, is a pivotal contributor to the massive morbidity and £billions/year burden of osteoarthritis and delayed fracture repair. EO is sensitive to local biomechanics, yet these control mechanisms remain ill‐defined. To decipher these mechanisms, our studies exploit in vivo tibia loading to characterize growth plate (GP) responses to defined mechanical stimuli.

In vivo μCT scanning of 12‐week‐old mice approaching skeletal maturity revealed that transient applied dynamic compressive loads (six episodes, comprising 40, 2 Hz 12 N cycles over 2 weeks) initially delayed but later accelerated EO, culminating in significantly longer tibias 12 weeks thereafter. Specifically, mean tibia length change (versus starting length) was 36.5% greater (p < 0.01) 8 weeks after commencement of the loading protocol, compared to basal growth‐related changes in non‐loaded limbs. This acceleration was preserved 12 weeks post‐loading, when mean tibia length change was 58.9% (p < 0.01) greater than in non‐loaded limbs. To pinpoint the specific chondrocyte stage(s) targeted, studies focused on acute load‐related changes in GP chondrocyte proliferation optimized in younger 3‐week‐old or 6‐week‐old mice with inherently faster growth. BrdU‐dosing with immunofluorescent‐tracking of labeled nuclear DNA disclosed rapid (6 hours) dynamic load‐induced triggering of proliferation in proximal tibial GPs, with markedly raised numbers of labeled nuclei (versus contralateral) predominantly in the lateral compartment, where loads are known to be greater. This indicates that mechanical stimuli influence murine GPs by controlling resident chondrocyte proliferation to accelerate EO.

Additional studies analyzing three‐dimensional ultra‐high resolution joint scans, loaded in real‐time, from differently aged mice have disclosed marked deformation within the entirely cartilaginous GP of young 8‐week‐old mice, which lack the GP bone “bridges” seen in skeletally‐mature, 36‐week‐old or 60‐week‐old mice. Bone morphology reveals less epiphyseal trabecular bone, more porous basal plates and thinner metaphyseal trabeculae, which imply less effective metaphyseal load‐transfer in younger mice. Ongoing digital volume correlation analysis is seeking to measure intra‐GP strains directly in such images to define GP deformation in healthy, aging and OA mice for the first time.

Current focus is on: (i) developing quantitate methods for regional assessment of BrdU‐EdU labeling to define spatiotemporal relationships to GP load‐engendered strains; (ii) examining responses at alternative endpoints (4–24 hours) to fully characterize acute proliferative response trajectory; and (iii) histological evaluation of changes in GP zone heights and organization. It is envisioned that merging of studies, measuring load‐induced GP deformation, proliferation, changes in zonal organization, bridge formation and growth rates within our tibial loading model will together define roles for load‐induced mechanical stimuli in controlling EO.

## Transcriptomic analysis reveals a link between mitochondrial dysfunction and experimental chronic kidney disease–mineral bone disorder

### Shun‐Neng Hsu ^1,2^, Louise A. Stephen^1^, Kanchan Phadwal^1^, Scott Dillon^3^, Roderick Carter^1^, Amanda Novak^1^, Katie Emelianova^1^, Elspeth Milne^1^, Behzad Javaheri^4^, Andrew Pitsillides^4^, Vicky E. Macrae^1^, Tom Freeman^5^, Katherine Staines^6^, Colin Farquharson^1^

#### ^*1*^ *University* *of Edinburgh, Edinburgh, UK.* ^*2*^ *Tri‐Service* *General Hospital, Taipei, Taiwan.* ^*3*^ *University* *of Cambridge, Cambridge, UK.* ^*4*^ *The* *Royal Veterinary College, London, UK.* ^*5*^ *The* *Janssen Pharmaceutical Companies of Johnson and Johnson, Pennsylvania,* *USA* *.* ^*6*^ *University* *of Brighton, Brighton, UK*

**Abstract**

**Objectives**: Chronic kidney disease–mineral and bone disorder (CKD‐MBD) is a systemic disorder that presents with dysregulated mineral metabolism causing ectopic calcification and compromised bone formation. It is characterized by hyperphosphatemia, elevated parathyroid hormone (PTH), as well as fibroblast growth factor‐23 (FGF23) levels. Uremic toxins, eg, indoxyl sulfate (IS), that promote oxidative stress are also associated with the development and progression of CKD in humans. To obtain an improved understanding of how this altered systemic milieu results in CKD‐MBD, we used whole transcriptome sequencing (RNA‐seq) to identify possible molecular mechanisms responsible for the poor bone health in CKD‐MBD.

**Methods:** To induce CKD‐MBD, male C57BL/6 mice were fed a diet containing 0.6% calcium, 0.9% phosphate, and 0.2% adenine. Control mice received the same diet without adenine. Tissue was analyzed at 5‐day intervals over a 35‐day time course to identify temporal changes with disease progression. Serum was analyzed for BUN, creatinine, calcium, phosphorus, PTH, FGF23, and bone turnover markers. Urine specific gravity (SG) and albumin and creatinine concentration were determined. The architecture and bone mineral density of cortical and trabecular bone were determined by μCT and histomorphometry. RNA‐seq analysis was completed on femurs (marrow removed) and data were analyzed by Graphia software. In vitro experiments of mitochondrial function in murine primary osteoblasts treated with IS (2mM) were completed to extend the RNA‐seq results.

**Results:** CKD mice presented with hyperphosphatemia, hyperparathyroidism, and elevated FGF23 BUN, phosphorus, and serum creatinine concentrations together with decreased urine SG, albumin, and creatinine. Kidney fibrosis was also noted. μCT and histomorphometry revealed osteopenia with structural changes to both trabecular and cortical bone in CKD mice (*p* < 0.01). Trabecular bone mineralization was decreased (*p* < 0.01) and bone turnover markers were increased (*p* < 0.01). Genes with a 1.5‐fold change in expression levels (FDR <0.05) from day 20 and 35 time points (CKD versus control) were selected to establish gene clusters associated with CKD. An unexpected finding was the significant downregulation of genes involved in mitophagy, mitochondrial function, and oxidative phosphorylation in CKD bones. In isolated osteoblasts, IS treatment altered mitochondrial morphology, increased production of reactive oxygen species, and reduced Parkin expression, mitochondrial membrane potential, and oxidative phosphorylation.

**Conclusion:** This is the first transcriptomic study of bone in an experimental model of CKD‐MBD. Our findings are consistent with the hypothesis that mitochondrial dysfunction contributes to the altered bone remodeling characteristic of CKD‐MBD. The full clinical implications of this conclusion are still to be determined.

## Bioengineering

## 3D printed regenerative and mechanical competent bone implant as a treatment for hip dysplasia

### Miguel Castilho ^1,2^, Nasim Golafshan^1^, Koen Willemsen^1^, Harrie Weinans^1^, Bart van der Wal^1^, Jos Malda^1^

#### ^*1*^ *University* *Medical Center Utrecht, Utrecht, The Netherlands.* ^*2*^ *Eindhoven* *University of Technology, Eindhoven, The Netherlands*

**Abstract**

Osteoarthritis (OA) of the hip is a painful and debilitating condition that affects over 40 million people just in Europe. One of the two main causes for hip OA is hip dysplasia, which is an instability of the hip joint. This instability is caused by incomplete coverage of the femoral hip by the acetabulum and is commonly observed in humans, including children, as well as in veterinary patients, mainly dogs (Fig. 1*A*). Currently, the most common surgical methods to correct the dysplasia involve the realignment of the hip socket (osteotomy) or the insertion of a bone graft to enlarge the socket (shelf arthroplasty) (Fig. 1*B*). Although the osteotomy is invasive, the shelf arthroplasty requires donor tissue. In addition, both procedures only have limited success rates. Here, we developed a 3D‐printed regenerative, yet stable, bone implant for the treatment of hip dysplasia that can overcome the drawbacks with current treatments.

A patient‐specific hip implant was designed based on CT scans of three cadaveric dogs (Fig. 1*C*) and 3D printed using a room temperature extrusion printing process and a biomaterial ink, composed of a magnesium phosphate (MgP) ceramic phase, modified with osteoinductive strontium (Sr^2+^) ions (MgPSr), and a degradable polycaprolactone (PCL) polymer phase. After implant fabrication, the effect of the implant composition and structure on mechanical stability, fixation to host bone and osteoconductive properties were investigated. To anticipate the in‐vivo mechanical performance of the resorbable implant, the implant was tested under physiological loading conditions using a custom‐made (bio)mechanical setup.

The 3D printing process and biomaterial ink, allowed the successful fabrication of a patient specific hip implant that ensured perfect fit to the hip socket of a canine dysplastic hip (Fig. 1*D*). Moreover, mechanical and in vitro biological characterization confirmed the flexible and bone‐inducing properties of the implant. Finally, ex vivo biomechanical evaluation confirmed that the developed implant can be fixed with metallic screws and, importantly, support physiological loading conditions, even after accelerated in vitro degradation (Fig. 1*E*,*F*). This novel implant opens up great perspectives with respect to the treatment of hip dysplasia as it provides a stable, long‐term, and regenerative solution that can fully integrate with the host bone tissue. Moreover, the regenerative nature offers great potential for the treatment of young (pediatric) patients.

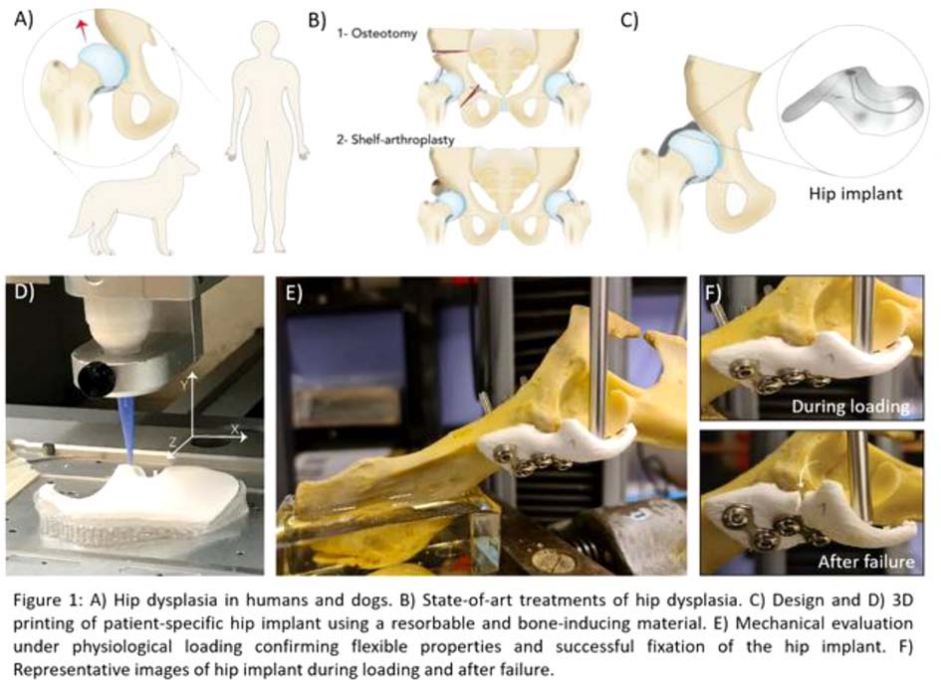

## Cellular and Molecular

## PHOSPHO1, matrix vesicles and biomineralization

### Scott Dillon ^1^, Louise Stephen^1^, José Luis Millán^2^, Fabio Nudelman^1^, Colin Farquharson^1^

#### ^*1*^ *University* *of Edinburgh, Edinburgh, UK.* ^*2*^ *Sanford* *Burnham Prebys Medical Discovery Institute, La Jolla,* *USA*

**Abstract**

Bone biomineralization is a fundamental process critical to the development of the skeleton and is orchestrated by nanoscopic extracellular vesicles known as matrix vesicles (MVs). MVs are embedded in the mineralizing extracellular matrix and are hypothesized to accumulate amorphous calcium and inorganic phosphate to induce hydroxyapatite mineral nucleation. The phosphatase orphan phosphatase 1 (PHOSPHO1) has been shown to be critical to this process and young PHOSPHO1‐null (*Phospho1* ^*−/−*^) mice exhibit severe bone hypomineralization. The biochemical mechanism through which PHOSPHO1 substrates are generated within MVs, however, is still unknown.

Skeletal development in *Phospho1* ^*−/−*^ and wild‐type animals was characterized in mouse embryos at 17 days of development (E17) using mesoscopic optical projection tomography (OPT) while the ultrastructure was characterized using focused ion beam‐scanning electron microscopy (FIB‐SEM) and transmission electron microscopy (TEM). To understand the biogenesis and biochemistry of MVs vesicles were isolated by differential ultracentrifugation from primary osteoblast cultures under mineralizing conditions and subjected to mass spectrometry.

OPT revealed a marked loss of mineralized bone throughout the skeleton in *Phospho1* ^*−/−*^ animals compared to controls (*p* < 0.001) affecting both endochondral and intramembranous‐derived bones. The ultrastructure of wild‐type embryonic bone investigated by FIB‐SEM exhibited a distinct core of mineralized material associated with a forming surface characterized by mineralization foci propagating through a fibrous matrix (Fig. 1). In contrast, *Phospho1* ^*−/−*^ bone was entirely fibrous in places with numerous electron dense particles embedded throughout a collagenous network (Fig. 1). Correlated TEM revealed these to be unmineralized MVs containing osmophilic material but no mineral crystals.

Lipidomics performed on vesicle isolates showed a significant enrichment of some lysophospholipid species in *Phospho1* ^*−/−*^ MVs (*p* < 0.05), supporting the hypothesis that the breakdown of vesicle membrane phospholipids contributes to intravesicular generation of PHOSPHO1 substrates. Characterization of the vesicle proteome by mass spectrometry was also performed to gain insights into potential mechanisms of vesicle biogenesis. Protein interaction network analysis revealed a potential role for RhoA/Rac1 GTPase signaling in integrating protein trafficking to determine vesicle cargo and cytoskeletal dynamics during vesicle budding.

PHOSPHO1 is critical for embryonic bone development and its ablation prevents mineral nucleation from MVs. The generation of PHOSPHO1 substrates within vesicles likely occurs via breakdown of the vesicle membrane, however further detailed experiments are required to fully elucidate the detailed biochemistry occurring within MVs.

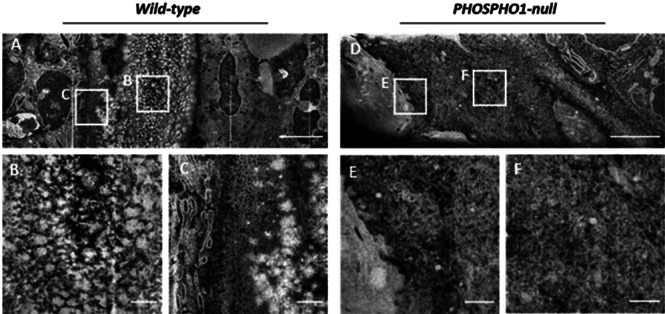

## Why do a third of all cats lose their teeth? The genetics of unruly odontoclasts in feline tooth resorption

### Seungmee Lee^1^, Brandon Shek^1^, Colin Farquharson^1^, Gurå T Bergkvist ^1,2^

#### ^*1*^ *Roslin* *Institute, The University of Edinburgh, UK.* ^*2*^ *R* *(D)* *SVS* *, The University of Edinburgh, UK*

**Abstract**

**Background:** Feline tooth resorption (TR) is a painful and progressive disease commonly diagnosed in small animal practice and characterized by loss of the mineralized tissues of the tooth. Surveys of the feline population reports incidences of up to 29% in the mixed breeds but this increases to 75% in some purebred populations. Odontoclasts are the osteoclasts of the dental microenvironment responsible for the resorption of milk teeth to allow eruption of the permanent dentition. Unlike bone, teeth do not remodel so odontoclasts have a very limited role later in life. In TR however the odontoclasts become reactivated and start to attack the adult dentition (Figure).

**Methods and Results:** We performed RNAseq of the dental transcriptome of TR‐ve and TR+ve teeth and identified 1732 differentially expressed genes. Pathway analysis highlighted a number of genes of interest involved in osteoclast differentiation and calcium signaling; three of these have been investigated further: matrix metalloproteinase 9 (MMP9), cathepsin K (CTSK), and purinergic receptor P2X4 (P2X4R). Single nucleotide polymorphism (SNP) analysis of the RNAseq data revealed high‐impact SNPs associated with osteoclast differentiation and activity exclusively in TR+ve (coenzyme Q4, myosin heavy chain 13, mitochondria encoded cytochrome B) and TR‐ve teeth (MMP14). Immunohistochemistry co‐localized the expression of MMP9, CTSK, and P2X4R to TRAP positive, multinucleated cells located in resorption pits on the mineralized surfaces of TR+ve teeth. The effect of inhibition of MMP9 during odontoclastogenesis was investigated using a semi‐selective MMP9 inhibitor and siRNAs. Preliminary investigations into the effect of CTSK and P2X4R siRNAs were performed. Drug inhibition of MMP9 caused a dose‐dependent reduction in odontoclast numbers and resorption activity. Targeting of MMP9 using siRNA resulted in decreased odontoclast numbers but had no impact on the numbers of resorption pits formed on dentine when compared to scrambled controls. When repeated using osteoplates, a decrease in resorption area was observed between MMP9 and scrambled controls. Significantly less TRAP positive cells formed on plastic following electroporation with siRNAs against CTSK and P2X4R when compared to scrambled controls.

**Conclusions:** By mapping the pattern of expression of these genes and their gene products during odontoclast differentiation and investigating the effect on odontoclast activity when blocking them, we can elucidate their role in disease initiation and progression. This could form the basis for the development of genetic tests to help breed down the incidence in the purebred cat population, as well as identify potential future drug targets.

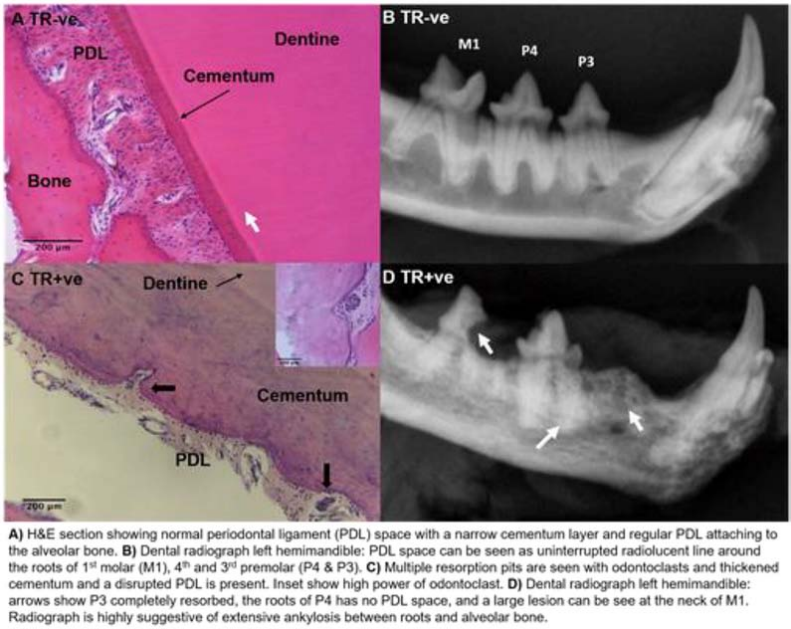

## Caloric restriction abrogates vascular calcification by autophagic and proteasomal degradation of RUNX2, a key osteogenic transcription factor

### Kanchan Phadwal ^1^, Dominic Kurian^1^, Benjamin Thomas^2^, Eve Koo^1^, Nicholas Morton^2^, Dongxing Zhu^3^, Will Cawthorn^2^, Vicky MacRae^1^

#### ^*1*^ *The* *Roslin Institute R(D)* *SVS* *, University of Edinburgh, Easter Bush, Edinburgh, UK.* ^*2*^ *Centre* *for Cardiovascular Science, The Queens Medical Research Institute, University of Edinburgh, Edinburgh, UK.* ^*3*^ *School* *of Basic Medical Sciences, Guangzhou Medical University, Guangzhou, China*

**Abstract**

Vascular calcification (VC), a pathophysiological consequence of atherosclerosis, is associated with a stiffened arterial system, leading to the development of cardiovascular disease. Currently, there are no effective strategies for treating vascular calcification. Caloric restriction (CR) is a dietary intervention that involves reduction of total calories below ad libitum (AL) intake without nutritional insufficiency or malnutrition. Over the past 20 years, the potential for CR to delay the onset of age‐related chronic diseases and offer direct cardioprotective effects has become increasingly well recognized. However, the effect of CR on VC has yet to be examined.

VSMCs from the aortas of CR mice were isolated and cultured under calcifying conditions (3mM Pi). VSMCs derived from CR mice showed reduced calcification compared to VSMCs from AL mice (*p* < 0.0001). Calcified VSMCs from CR mice show reduced protein expression of runt‐related transcription factor (RUNX2) (two fold, *p* < 0.05). Comparable results were obtained following treatment with the CR mimetic metformin (1μM) and in vitro starvation in cell culture. Investigation of the latter treatment by stable isotope labeling using amino acids in cell culture (SILAC) based quantitative mass spectrometry further identified upregulation of ubiquitin proteasome and autophagy pathway proteins.

To investigate the regulation of RUNX2 by CR, we first blocked its degradation via the ubiquitin proteasome pathway (UPS) with MG132 (0.5μM). Next, we employed Bafilomycin‐A (10nM), a selective V‐ATPase inhibitor to block autophagic trafficking. Blocking both trafficking pathways increased RUNX2 expression in calcifying VSMCs derived from CR mice (two fold, *p* < 0.05). Silencing key genes underpinning UPS (Cullin2 and UBE2H) and autophagy (ATG3 and LAMP1) pathways increased VSMC calcification (*p* < 0.05). Furthermore, calcified VSMCs treated with metformin show translocation of RUNX2 from the nucleus to the cytoplasm where RUNX2 is present with in the autophagosomes. Silencing Atg3 blocks the translocation of nuclear RUNX2 to cytoplasm suggesting a novel mechanism of RUNX2 degradation via autophagosomes.

In summary, our data strongly suggests that CR and the CR mimetic metformin inhibit VC by promoting autophagy and UPS‐mediated degradation of RUNX2. This novel finding could lead to the application of CR‐mimetics to combat vascular calcification.

## A simple and robust in vitro method for long‐term osteocyte 3D culture using either primary or immortalized cell lines

### Melissa Finlay ^1^, Georgiana Neag^1^, Jonathan Lewis^1^, Erik A B Hughes^2^, Miruna Chipara^2^, Laurence Hill^2^, Kieran Patrick^1^, Lisa J Hill^3^, James R Edwards^4^, Liam M Grover^2^, Amy J Naylor^1^

#### ^*1*^ *Institute* *of Inflammation and Ageing, University of Birmingham, Birmingham, UK.* ^*2*^ *School* *of Chemical Engineering, University of Birmingham, Birmingham, UK.* ^*3*^ *Institute* *of Clinical Sciences, University of Birmingham, Birmingham, UK.* ^*4*^ *Nuffield* *Department of Orthopaedics, Rheumatology, and Musculoskeletal Sciences, Botnar Research Centre, University of Oxford, Oxford, UK*

**Abstract**

Bone remodeling is the intercellular process that is vital for bone development, maintenance, and repair; dysregulation of which leads to progressive and debilitating bone conditions, like osteoporosis. The increasing demand for effective drug therapies and ethical concerns about the use of animals in research warrants the development of in vitro models that can recapitulate the bone remodeling cycle. Advances in our understanding of the bone remodeling cycle have demonstrated that the osteocyte is central to its control, by regulating the differentiation and activity of the major bone cells involved, the osteoclasts and the osteoblasts.

At present, in vitro bone models lack osteocytes, due to the challenging and demanding conditions required for maintaining them in long‐term culture. We have developed a Self‐Structuring Bone Model (SSBM) that can support osteoblasts and osteocytes long‐term and provide a platform for bone remodeling studies in vitro, by building on a successful methodology developed at the University of Birmingham.

An immortalized human osteoblast cell line, hFOB 1.19, was seeded and cultured on fibrin hydrogels that were prepared and formed between two calcium phosphate cement anchors. Over a subsequent 12‐week culture, the cells cause contraction of the hydrogel scaffold around the anchors, forming a dense fibrin scaffold. The matrix of this scaffold is gradually replaced with a progressively mineralizing, collagen‐rich, extracellular matrix, confirmed using imaging techniques including XRF, confocal microscopy, and μCT. The progress of construct formation and maturation was compared against similar constructs seeded with primary rat periosteal cells (as used in the original SSBM development study: Iordachescu et al. 2019) and our initial findings show that this methodology is transferable and repeatable using the hFOB 1.19 cell line. Further work is ongoing to fully characterize the degree of osteocyte differentiation, as well as the development of the constructs' structural composition over the experimental timeframe using qPCR, ELISA, and scanning electron microscopy analysis.

The need for a simple, reliable and reproducible in vitro bone model is apparent. Here we describe the first 3D human‐derived self‐structuring construct of its kind to differentiate and culture osteoblasts and osteocytes long‐term. Our ambition now is to use this as a platform to enable complete recapitulation of the bone remodeling cycle in vitro.

## Proteomic identification of the targets of the pro‐metastatic transcription factor TWIST1 within breast cancer bone metastasis

### Steven Wood, Ana Lopez, Phillippe Clezardin, Janet Brown

#### University of Sheffield, Sheffield, UK

**Abstract**

**Rationale and hypothesis:** Considerable progress has been made in breast cancer treatment; however, despite this, metastatic spread is currently a major cause of patient morbidity and reduced quality of life. Bone is one of the major sites of metastatic spread within breast cancer, with spread to the skeleton being detectable in over 70% of advanced breast cancer patients. The transcription factor TWIST1 and its downstream target micro‐RNA (miR‐10b) have been identified as key regulators in the early stages of metastasis of cancers. Mechanistic understanding of the genes and proteins regulated by TWIST1 – miR‐10b signaling will yield novel drug targets (and potential biomarkers) relevant to the early stages of cancer spread.

**Objectives:** The complete set of downstream targets of miR‐10b within bone‐metastatic breast cancer cells cannot be predicted by in silico analysis alone. Thus, state‐of‐the‐art proteomic methods were used to identify the proteins which alter in expression in response to miR‐10b.

**Methodology:** A bone metastatic variant of the triple negative breast cancer cell line MDA‐MB‐231 (termed BOM2) was transfected with either miR‐10b synthetic oligonucleotides or control oligonucleotides. Quantitative proteomics using pulsed SILAC (pSILAC) was employed to identify the proteins which alter in expression with the intracellular compartment of these cells in response to miR‐10b. In a complimentary study, bio‐orthogonal noncanonical amino acid tagging (BONCAT) was used, to identify the proteins differentially secreted by these cells in response to miR‐10b.

**Results:** Over 6000 proteins were identified and quantified within the BOM2‐cell lysates, with 407 proteins displaying a statistically significant difference in expression (*p* < 0.05), in response to miR‐10b (using four biological replicates) (see Fig. 1). Extensive bioinformatic analysis has resulted a panel of 10 proteins, taken forward for validation.

In a parallel series of experiments, the BONCAT method identified over 40 proteins differentially secreted by BOM2 cells upon expression of miR‐10b (see Fig. 1). These differentially secreted proteins include key components of extra‐cellular matrix remodeling and cell‐signaling.

**Conclusion:** Proteomic analysis of the bone homing BOM2 cell line has identified a panel of both intracellular and secreted proteins, which alter in response to the pro‐metastatic micro‐RNA miR‐10b. Identification of these proteins has provided insights into the mechanism of action of miR‐10b within the early stages metastasis. In addition, they are being further pursued as potential biomarkers within the clinical management of breast cancer.

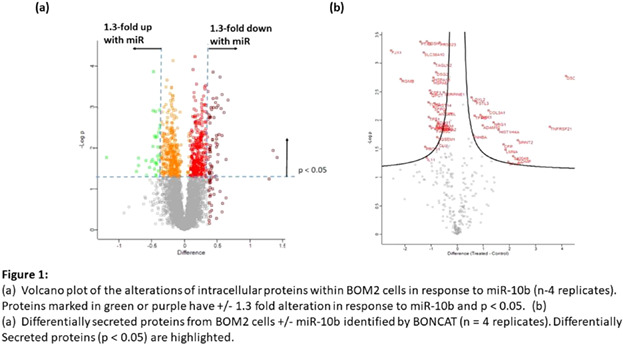

## Clinical Cases

## Vanishing bones and persistent pain resistant to treatment

### Faidra Laskou ^1,2^, Claire Holmes^2^, Justin Davies^2^, Madeleine Sampson^2^, Ray Armstrong^2^, Brian Davidson^2^, Kassim Javaid^3^, Elaine Dennison^1,2^

#### ^*1*^ *MRC* *Lifecourse Epidemiology Unit, Southampton, UK.* ^*2*^ *University* *Hospital Southampton* *NHS* *Foundation Trust, Southampton, UK.* ^*3*^ *Nuffield* *Department of Orthopaedics, Rheumatology and Musculoskeletal Sciences, University of Oxford, Oxford, UK*

**Abstract**

**Introduction:** Multicentric carpotarsal osteolysis (MCTO) is a rare skeletal dysplasia caused by mutations in *MAFB*. Individuals present with demineralization and osteolysis mainly affecting the carpal and tarsal bones. MCTO can be associated with progressive nephropathy and corneal clouding. We present a MCTO case and her challenging treatment path.

**Case:** The 23‐year‐old woman first presented at the age of 3 years with a right distal radius fracture and radiographic evidence of osteolysis affecting her right carpal bones. Genetic testing confirmed heterozygosity for the p.Ser69Leu mutation. Her father presented as an inflammatory type of arthritis at the age of 5 years and was known to have de novo mutation. Due to ongoing pain and progressive osteolysis, she received a number of treatments including IV Pamidronate monthly for 20 months (weight‐adjusted dose ∼37 mg monthly). Due to concerns over long‐term use of bisphosphonates at her age, and her ongoing pain, Pamidronate was discontinued and calcitonin (200 IU intranasally followed by subcutaneous injections of 50 IU/3x week) initiated. Osteolysis progressed affecting both her carpal and tarsal bones and she was walking to limited extent by the age of 5 years. Following a drug holiday of 2 years, clinical progression, and ongoing pain, she was treated with IV zoledronic acid (first dose of 0.0125 mg/kg followed by 0.025 mg/kg) every 6 months, and had received four doses in total when she presented with a fracture of her medial column of distal humerus and was admitted with urinary sepsis. In 2011, 40 mg Denosumab injections 6‐monthly (four doses) were initiated followed by 2‐monthly 60‐mg injections (July 2013 to November 2013). Denosumab dose increased to monthly injections from January 2014 until June 2014 when she presented with an atypical subtrochanteric right femur fracture that was managed with an intramedullary nail and 18 months of teriparatide. Unfortunately, her condition has progressed radiologically showing complete osteolysis of the carpal bones, right elbow, humeral trochlear is essentially dissolved, and erosions in both feet. She has a low‐level proteinuria managed with an ACEI (father had primary focal segmental glomerulosclerosis). Club‐shaped metaphyses and Pamidronate‐induced osteopetrosis noted on X‐rays. Serial DXA scans show normal bone density and HRpQCT (2011–2017) are unchanged.

**Conclusion:** We present a case of MCTO that has been very challenging to manage. Imaging and genetic testing pays a crucial role in establishing the diagnosis but MCTO management often raises important and difficult treatment questions. Currently, targeted therapies are lacking.

## Experiences of a virtual multidisciplinary team meeting process to review unsolved musculoskeletal families from the 100 K Genomes Project

### Alistair Pagnamenta ^1^, Meena Meena Balasubramanian^2^, Christine Burren^3^, Raymond Dalgleish^4^, Julie Evans^5^, Genomics England Research Consortium^6^, Edoardo Giacopuzzi^1^, Melita Irving^7^, Kassim Javaid^1^, Ruth Newbury‐Ecob^3^ , Amaka Offiah^8^, Ataf Sabir^9^, Debbie Shears^10^, Sarah Smithson^3^, Jenny Taylor^1^, Andrew Wilkie^1^, Louise Wilson^11^

#### ^*1*^ *University* *of Oxford, Oxford, UK.* ^*2*^ *Sheffield* *Children's* *NHS* *Foundation Trust, Sheffield, UK.* ^*3*^ *University* *Hospitals Bristol and Weston* *NHS* *Foundation Trust, Bristol, UK.* ^*4*^ *University* *of Leicester, Leicester, UK.* ^*5*^ *South* *West Genomic Laboratory Hub, Bristol, UK.* ^*6*^ *Consortium* *members listed at* www.genomicsengland.co.uk/about-gecip/publications *, London, UK.* ^*7*^ *Guy* '*s and St Thomas'* *NHS* *Foundation Trust, London, UK.* ^*8*^ *University* *of Sheffield, Sheffield, UK.* ^*9*^ *Birmingham* *Women's and Children's* *NHS* *Foundation Trust, Birmingham, UK.* ^*10*^ *Oxford* *University Hospitals NHS Foundation Trust, Oxford, UK.* ^*11*^ *Great* *Ormond Street Hospital for Children, London, UK*

**Abstract**

To shorten the diagnostic odyssey for rare disease patients, the 100,000 Genomes Project (100KGP) undertook genome sequencing (GS) for rare disease and cancer patients. A subsidiary aim of the project was to establish the utility of GS in the NHS. The musculoskeletal domain recruited 750 families in whom a clinical diagnosis of osteogenesis imperfecta, unexplained skeletal dysplasia, multisuture/syndromic craniosynostosis, or multiple epiphyseal dysplasia had been made (Table). GS data were assessed using standard clinical bioinformatic pipelines focusing on curated gene‐panels. Initial diagnostic yields across these categories was 12%–31%. Here we present preliminary findings arising from closer scrutiny of specific families of interest that remained unsolved.

Recruiting clinicians were invited to attend a virtual multidisciplinary team (MDT) meeting to present families and assess the results of further bioinformatic analyses. Additional analyses included searching for homozygous loss‐of‐function variants and manual review of read‐alignments for genes implicated based on a strong clinical suspicion for a specific diagnosis. Clinicians were asked to circulate background information prior to meeting, including the suspected diagnosis and sets of genes/pathways potentially involved.

Multiple disciplines attended the meeting, including clinical geneticists, adult and pediatric endocrinologists/rheumatologists, musculoskeletal radiologists and data analysts. Of seven families presented over two sessions, four are now considered solved. This included a three‐generational family with suspected Greig syndrome where targeted *GLI3* sequencing performed in 2015 had been negative. We identified a 1.2 Mb chromosome 7 inversion disrupting *GLI3* (Figure). A separate scenario involves an affected father‐daughter with hypophosphatemic rickets where a 112‐kb duplication from 11p15.5 had been inserted into intron 9 of *PHEX*. A recommendation was made that RNA analysis should be performed to confirm the effects of this rearrangement at the transcript level. Other individuals were identified with homozygous frameshifts in *PRKG2* and *PKDCC*, helping to confirm recently proposed skeletal dysplasia disease genes. For the remaining families, recommendations were made for additional genes to analyze (*n* = 2), further radiological investigations (*n* = 1) and sampling of the parents to complete the trio (*n* = 1).The experience from running our first MDT meetings highlighted the need for close collaboration with the clinical community when analyzing GS data. Furthermore, the process supports a strategy to select high priority genes and pathways based on characteristic phenotypic features.

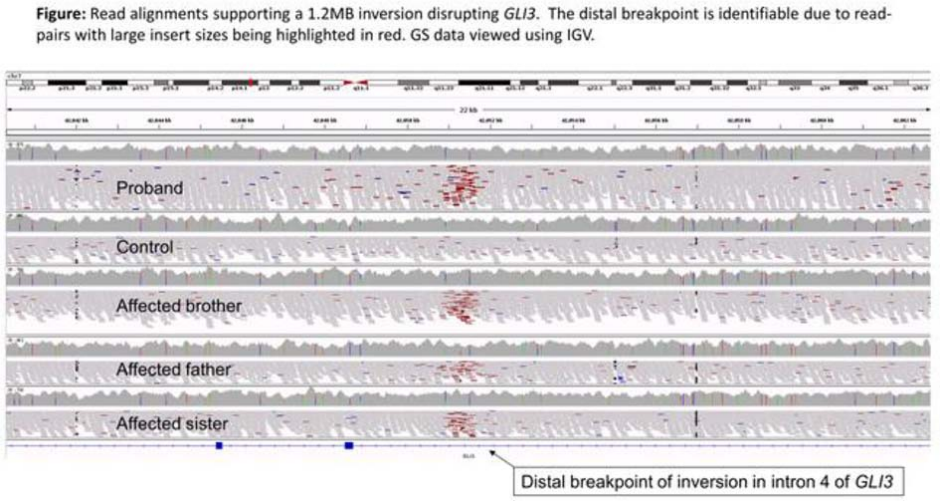

**Table:** **Table.** Diagnostic yield by disease in the 100KGP from the exit questionnaire (v11)

Specific disease	Families	Diagnostic yield
Osteogenesis imperfecta	363	31.4%
Unexplained skeletal dysplasia	237	13.5%
Craniosynostosis syndromes	117	12.0%
Multiple epiphyseal dysplasia	33	21.2%

## Common MSK Disorders

## Osteophyte size and location rather than joint space width are associated with hip pain: findings from UK Biobank

### Benjamin Faber ^1^, Raja Ebsim^2^, Fiona Saunders^3^, Monika Frysz^1^, Claudia Lindner^2^, Jenny Gregory^3^, Richard Aspen^3^, Nicholas Harvey^4^, George Davey Smith^1^, Timothy Cootes^2^, Jon Tobias^1^

#### ^*1*^ *University* *of Bristol, Bristol, UK.* ^*2*^ *The* *University of Manchester, Manchester, UK.* ^*3*^ *University* *of Aberdeen, Aberdeen, UK.* ^*4*^ *University* *of Southampton, Southampton, UK*

**Abstract**

**Objective:** It remains unclear how the different features of radiographic hip osteoarthritis (rHOA) contribute to hip pain. We examined the relationship between rHOA, including its individual components, and hip pain using a novel dual‐energy X‐ray absorptiometry (DXA)‐based method.

**Methods:** Left hip DXAs were obtained from UK Biobank. An automated method was developed to obtain minimum joint space width (mJSW) in millimeters (mm) from points placed around the femoral head and acetabulum with a Random Forest machine learning–based approach. Osteophyte areas measured in square millimeters (mm^2^) at the lateral acetabulum, superior and inferior femoral head were derived manually. Semiquantitative measures of osteophytes and joint space narrowing (JSN) were combined to provide a measure of rHOA. Logistic regression was used to examine the relationships between these variables and hip pain, obtained via questionnaires. The adjusted model included the demographic covariates: age, sex, height, weight, and ethnicity.

**Results:** A total of 6807 left hip DXAs were examined. rHOA was present in 353 (5.2%) individuals and was associated with hip pain (OR 2.07; 95% confidence interval [CI], 1.54–2.80) and hospital diagnosed OA (OR 5.73; 95% CI, 2.89–11.36). Total osteophyte area and mJSW were associated with hip pain (1.29 [95% CI, 1.21–1.36], 0.84 [95% CI, 0.77–0.92]), respectively) in unadjusted models. After mutually adjusting and adding demographic covariates, total osteophyte area continued to have strong evidence of association with hip pain (1.31; 95% CI, 1.23–1.39), but mJSW did not (0.95; 95% CI, 0.87–1.04) (Fig. 1). Acetabular, superior and inferior femoral head osteophyte areas were all independently associated with hip pain (1.19 [95% CI, 1.13–1.26], 1.22 [95% CI, 1.15–1.29], and 1.21 [95% CI, 1.14–1.28], respectively).

**Conclusion:** The relationship between DXA‐derived rHOA and prevalent hip pain is explained by osteophyte area rather than mJSW. Our results indicate it is bone formation in the form of osteophytes, rather than cartilage loss measured as mJSW, that is associated with hip pain. These results have potential implications for clinical care given change in mJSW is a common primary end point for drug trials in OA. Osteophytes at different locations showed important, partially independent, associations with hip pain, possibly reflecting the contribution of distinct biomechanical pathways that require further research.

**Figure:**
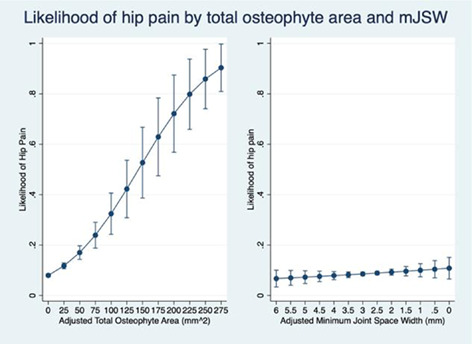
Figure 1: Left graph shows the likelihood of hip pain by total osteophyte area, adjusted for minimum joint space width (mJSW), age, height, weight, sex and ethnicity. Right graph shows likelihood of hip pain For Review Onlyby mJSW, adjusted for total osteophyte area, age, sex, height, weight and ethnicity, the x‐axis is reversed to reflect the direction of effect.

## Femoroacetabular impingement may not underlie the association between cam morphology and hip osteoarthritis: findings from UK Biobank

### Benjamin Faber ^1^, Raja Ebsim^2^, Fiona Saunders^3^, Monika Frysz^1^, Jenny Gregory^3^, Richard Aspden^3^, Nicholas Harvey^4^, George Davey Smith^1^, Timothy Cootes^2^, Claudia Lindner^1^, Jon Tobias^1^

#### ^*1*^ *University* *of Bristol, Bristol, UK.* ^*2*^ *The* *University of Manchester, Manchester, UK.* ^*3*^ *University* *of Aberdeen, Aberdeen, UK.* ^*4*^ *University* *of Southampton, Southampton, UK*

**Abstract**

**Objectives:** The biomechanical concept of femoroacetabular impingement (FAI) suggests that cam and pincer morphologies cause hip osteoarthritis (OA) due to aberrant biomechanical forces where the acetabulum impinges on the femoral head. Separately, acetabular dysplasia (AD) is thought to cause OA via joint instability due to lack of acetabular coverage of the femoral head. We aimed to examine whether the aforementioned hip morphologies are associated with radiographic hip osteoarthritis (rHOA) and hip pain, and, if so, whether they are associated with a distribution of osteophytes in keeping with FAI or more global joint degeneration.

**Methods:** Left hip dual‐energy X‐ray absorptiometry (DXA) scans taken on UK Biobank participants had alpha angle (AA), lateral center‐edge angle (LCEA), and joint space narrowing (JSN) derived using automatically placed points around the hip. Cam and pincer morphologies, and AD were defined using AA and LCEA (Fig. 1). Osteophytes were measured manually and the points were moved so as not to encompass them. rHOA grades were based on the presence of JSN and an osteophyte.

**Results:** A total of 6807 individuals were selected (mean age: 62.7 years; 3382/3425 males/females). Cam morphology was more prevalent in males than females (15.4% and 1.8%, respectively). In males, cam morphology was associated with rHOA (OR 3.20; 95% CI, 2.41–4.25), JSN (OR 1.53; 95% CI, 1.24–1.88), and acetabular (OR 1.87; 95% CI, 1.48–2.36), superior (OR 1.94; 95% CI, 1.45–2.57), and inferior (OR 4.75; 95% CI, 3.44–6.57) femoral head osteophytes, and hip pain (OR 1.48; 95% CI, 1.05–2.09). Broadly similar associations were seen in females, but with considerably weaker statistical evidence: rHOA (OR 2.47; 95% CI, 0.96–6.36), JSN (OR 1.75; 95% CI, 0.97–3.14), and acetabular (OR 0.99; 95% CI, 0.45–2.21), superior (OR 1.83; 95% CI, 0.72–4.67), and inferior (OR 10.07; 95% CI, 4.49–22.61) femoral head osteophytes, and hip pain (OR 1.11; 95% CI, 0.52–2.37). Neither pincer morphology nor AD showed any associations with rHOA or hip pain.

**Conclusions:** Cam morphology was predominantly seen in males in whom it was associated with rHOA and hip pain. In males and females, cam morphology was particularly strongly associated with inferior femoral head osteophytes. This suggests that FAI, assumed to cause acetabular and superior femoral head osteophytes, does not explain the relationship between cam morphology and OA. Furthermore, pincer morphology showed no association with rHOA casting doubt that FAI is important on a population level at causing OA.

**Figure:**
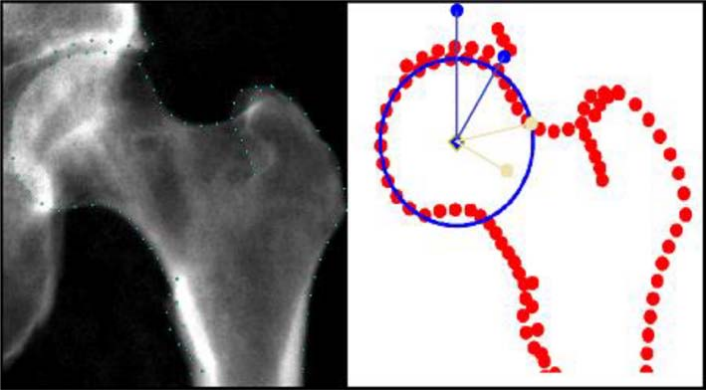
Figure 1. Left: DXA from UK Biobank with points. Right: a graphical representation of the points with a circle of best fit (blue) placed over the femoral head points that allows the LCEA (blue lines) and AA (yellow lines) to be derived.

## Midlife women's understanding of musculoskeletal health in older age: a qualitative study from Zimbabwe

### Nyasha Buwu ^1^, Sarah Drew^2^, Celia Gregson^2^, Rashida Ferrand^1,3^, Ruramayi Rukuni^3^, Rachael Gooberman‐Hill^2^

#### ^*1*^ *Biomedical* *Research and Training Institute, Harare, Zimbabwe.* ^*2*^ *University* *of Bristol, Bristol, UK.* ^*3*^ *London* *School of Hygiene and Tropical Medicine, London, UK*

**Abstract**

**Background:** Musculoskeletal conditions are a leading contributor to disability across sub‐Saharan Africa, with prevalence increasing with age. Understanding people's beliefs about health and illness in midlife can improve prevention and ensure healthcare delivery is appropriately designed, enabling healthcare to reach the people who need it. This study aimed to characterize the understandings and beliefs of midlife women towards musculoskeletal health in older age in Zimbabwe.

**Methods:** Twenty semi‐structured interviews were conducted with women aged 40–60 years in Harare, Zimbabwe. Purposive sampling identified women, taking into account age, comorbidities, HIV status and socio‐economic background. Data were audio‐recorded, transcribed and analyzed thematically.

**Results:** Participants provided their understandings about what happened to women's bones as they aged. These most commonly included joint, back, and leg pain. Most women described this as “eating,” “burning in the bones,” “biting,” and “aching.” Some talked about bones “losing strength,” making it more difficult to participate in everyday life. There was a belief that bones no longer healed after fracture or dislocation and that they could become brittle, “loose,” and fall out of place. Several contrasted these changes to the bones of children that they described as “healthy” and “active.”

Many women were unsure what caused these changes and saw them as an inevitable part of aging. Several provided biological explanations about arthritis, with participants explaining that loss of “gel” (synovial fluid) caused friction and pain. Being overweight was linked to musculoskeletal problems. Some thought excess weight made bones “ache” because of increased pressure, another thought that being overweight made them more likely to break bones if they fell. Participants also said that the bones in older people moved out of place due to lack of bone marrow. Inadequate nutrition was mentioned as a cause of musculoskeletal problems, both in terms of the types of foods older people ate and the quantity. Participants also talked about the impact of women's lifestyle that they contrasted with men's. Several thought that working hard, “carrying the weight of a man” during intercourse, pregnancy, and childbearing made their bones weaker. One woman attributed her joint pain to supernatural causes or “witchcraft.”

**Conclusions:** This study provides novel findings about understandings of musculoskeletal health and aging in midlife women in Zimbabwe. Information can be used to develop contextually appropriate educational and treatment interventions and enhance communication with healthcare professionals, improving musculoskeletal health in midlife women before they enter older age.

## Accelerated growth plate fusion has no effect on osteoarthritis vulnerability

### Hasmik Jasmine Samvelyan ^1^, Carmen Huesa^2^, Lucy Cui Lin^3^, Colin Farquharson^3^, Katherine Staines^1^

#### ^*1*^ *University* *of Brighton, Brighton, UK.* ^*2*^ *University* *of Glasgow, Glasgow, UK.* ^*3*^ *The* *University of Edinburgh, Edinburgh, UK*

**Abstract**

Osteoarthritis is the most prevalent systemic musculoskeletal disorder characterized by articular cartilage degeneration and subchondral bone sclerosis. Suppressor of cytokine signaling 2 (SOCS2) is an intracellular negative regulator of growth hormone signaling, thus mice deficient in SOCS2 (Socs2−/−) display accelerated bone growth. Here we sought to examine the contribution of accelerated growth to osteoarthritis development using this murine model of excessive longitudinal growth.

We examined the vulnerability of 16 week‐old male Socs2−/− mice to osteoarthritis following surgical induction of disease (destabilization of the medial meniscus [DMM]), and with aging (12–13 months old), by histology and micro‐computed tomography (μCT).

We observed increase in the number and density of growth plate bridges in Socs2−/− tibias in comparison to wild‐type (WT), regardless of osteoarthritis intervention (Fig. 1). Histological examination of WT and Socs2−/− knee joints revealed the development of osteoarthritic articular cartilage lesions with DMM in comparison to sham (WT DMM:3.4 ± 0.4; WT sham:0.3 ± 0.05; KO DMM:3.2 ± 0.8; KO sham:0.8 ± 0.3; *p* < 0.05). Articular cartilage lesion severity scores (mean and maximum) were similar in WT and Socs2−/− mice with either DMM, or with aging. μCT analysis revealed decreases in subchondral bone thickness, epiphyseal trabecular number and thickness in the medial compartment of Socs2−/− knee joints, in comparison to WT (*p* < 0.001). DMM had no effect on subchondral bone plate thickness in comparison to sham in neither WT nor Socs2−/− knee joints.

Together these data from the study of Socs2−/− mice suggest that enhanced growth hormone signaling on long bones accelerates growth plate fusion but has no effect on osteoarthritis vulnerability in this model.

**Figure:**
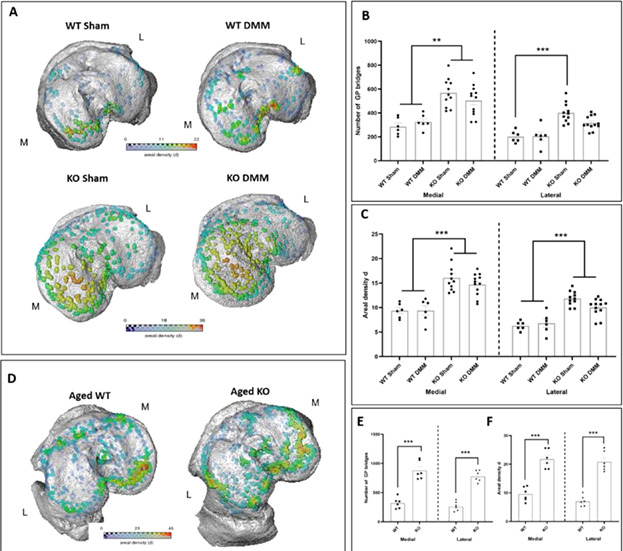
Figure 1. Socs2 deficient mice exhibit accelerated growth plate fusion mechanisms. (A) Location and areal densities of bridges across the growth plate projected on medial (M) and lateral (L) tibial joint surface in WT and Socs2‐/‐ (KO) DMM and sham operated joints. (B) Number of bridges. (C) Areal density (d) of bridges defined as number of bridges per 256 mm x 256 mm window. (D) Location and areal densities of bridges across the growth plate projected on medial (M) and lateral (L) tibial joint surface in aged WT and Socs2‐/‐ (KO) mice. (E) Number of bridges. (F) Areal density (d) of bridges defined as the number of bridges per 256 mm × 256 mm window. Data are presented as mean ± SEM and showing individual animals. Statistical test performed: Two‐way ANOVA with Bonferroni adjustments for multiple comparisons within each joint compartment. * p<0.05 ** p<0.01 *** p<0.001

**Epidemiology**

## Impact of menopause and HIV status on the peripheral skeleton of urban black South African women

### Mícheál Ó Breasail ^1^, Celia L Gregson^2,3^, Shane Norris^3^, Tafadzwa Madanhire^4,2^, Nicole Jaff^5^, Nigel Crowther^5^, Lisa Micklesfield^3^, Kate A Ward^6^

#### ^*1*^ *Medical* *Research Council Nutrition and Bone Health Research Group, Cambridge, UK.* ^*2*^ *Musculoskeletal* *Research Unit, Bristol Medical School, University of Bristol, Bristol, UK.* ^*3*^ *SAMRC* */Wits Developmental Pathways for Health Research Unit, Department of Paediatrics, School of Clinical Medicine, University of the Witwatersrand, Johannesburg, South Africa.* ^*4*^ *Biomedical* *Research and Training Institute, Harare, Zimbabwe.* ^*5*^ *Department* *of Chemical Pathology, National Health Laboratory Service and University of the Witwatersrand, Johannesburg, South Africa.* ^*6*^ *Medical* *Research Council Lifecourse Epidemiology, University of Southampton, Southampton, UK*

**Abstract**

**Introduction:** Menopause is associated with an accelerated loss of bone mineral; however, few data are available from sub‐Saharan African (SSA) populations. This analysis aimed to determine whether the menopause transition, and HIV status, were associated with bone density and strength at the distal radius, a common fracture site.

**Methods:** Forearm peripheral QCT data with verified menopause and HIV status were available for a subset (*n* = 430) of Black South African women, aged 40–61 years (*n* = 77 [18%] with HIV) from The Study of Women in and Entering Endocrine Transition. pQCT outcomes included: total cross‐sectional area (CSA), total volumetric bone mineral density (vBMD), and compressive bone strength (BSIc) at the distal 4% radius; and cortical vBMD, total CSA, and cortical thickness at the 66% proximal radius. Linear regression assessed the association between premenopausal, perimenopausal, and postmenopausal groups and pQCT outcome adjusting for age, height, and weight, and then stratified by HIV status, with mean (95% confidence interval [CI]) and tests for trend across menopausal groups presented.

**Results:** The 430 women were of age 49.2 ± 5.3 years (mean ± SD), with a body mass index (BMI) of 32.4 ± 6.3 m/kg^2^. After adjustment, transitioning through menopause was associated with lower distal (4%) radius total vBMD (premenopause: 345.7 [95% CI, 335.8–355.5] mg/cm^3^ versus postmenopause: 330.1 [95% CI, 322.7–337.6] mg/cm^3^), and BSIc (premenopause: 0.39 [95% CI, 0.37–0.41] g^2^/cm^4^ versus postmenopause: 0.36 [95% CI, 0.35–0.37] g^2^/cm^4^) (*p* = 0.017 and 0.012 for trend, respectively). Similar trends were observed in proximal (66%) radius cortical vBMD (premenopause: 1146.8 [95% CI, 1138.9–1154.6] mg/cm^3^ versus postmenopause: 1136.1 [95% CI, 1130.1–1142.0] mg/cm^3^) and cortical thickness (premenopause: 2.01 [95% CI, 1.95–2.06] mm versus postmenopause: 1.93 [95% CI, 1.89–1.98] mm) (*p* = 0.028 and 0.036 for trend, respectively). By contrast total CSA varied little by menopause at either scan site. When stratified by HIV status, distal radius total vBMD and BSIc were generally lower in perimenopausal and postmenopausal women compared to premenopausal, though only significant in the HIV‐negative group (Fig. 1). At the proximal radius advancing menopausal stage was associated with lower cortical vBMD, particularly in women with HIV (premenopause: 1152.9 [95% CI, 1128.5–1177.2] mg/cm^3^ versus postmenopause: 1123.6 [95% CI, 1106.0–1141.2] mg/cm^3^, *p* = 0.048 for trend).

**Conclusions:** In South African women, menopause is associated with bone loss, particularly at the distal radius, a common fracture site of osteoporotic fracture. Although the sample size was small, women with HIV had evidence of lower cortical density than their HIV negative counterparts. These findings raise concern for the incidence of Colles' fractures in postmenopausal women living with HIV infection, and larger longitudinal studies are required.

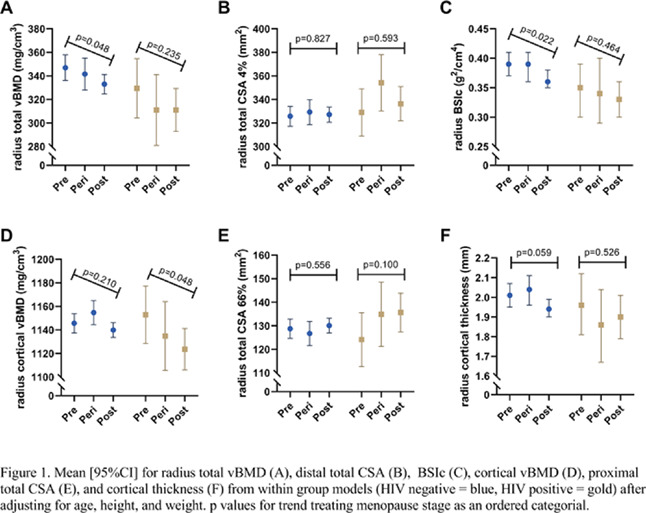

## The mediating role of endocrine factors in the positive relationship between fat mass and bone mass in children aged 9–11 years: The Physical Activity and Nutrition in Childhood Study

### Annie Constable ^1,2^, Dimitris Vlachopoulos^1^, Alan Barker^1^, Sarah Moore^3^, Sonja Soininen^2,4^, Eero Haapala^2,5^, Juuso Väistö^2^, Jarmo Jääskeläinen^6^, Raimo Voutilainen^6^, Seppo Auriola^2^, Merja Häkkinen^2^, Tomi Laitinen^7^, Timo Lakka^2,7,8^

#### ^*1*^ *University* *of Exeter, Exeter, UK.* ^*2*^ *University* *of Eastern Finland, Kuopio, Finland.* ^*3*^ *Dalhousie* *University, Halifax, Canada.* ^*4*^ *Social* *and Health Center, Varkaus, Finland.* ^*5*^ *University* *of Jyväskylä, Jyväskylä, Finland.* ^*6*^ *University* *of Eastern Finland and Kuopio University Hospital, Kuopio, Finland.* ^*7*^ *Kuopio* *University Hospital, Kuopio, Finland.* ^*8*^ *Kuopio* *Research Institute of Exercise Medicine, Kuopio, Finland*

**Abstract**

**Introduction**: Increased fat mass may increase bone mass through greater biomechanical load on bones. Further, fat mass has an endocrine function which may influence bone metabolism both positively and negatively. The mediating role of endocrine factors in the positive relationship between fat mass and bone mass in prepubertal and early‐pubertal children is unclear. The aim of this study was to examine the association between fat mass and bone mineral content (BMC), and to investigate whether this relationship is mediated by insulin, free leptin index, adiponectin, dehydroepiandrosterone sulfate, testosterone, and estradiol in girls and boys aged 9 to 11 years.

**Methods**: We utilized cross‐sectional data from the 2‐year follow‐up of the Physical Activity and Nutrition in Childhood study, an ongoing longitudinal study in a population sample of Finnish children (*n* = 396; 203 girls). Total body less head (TBLH) BMC and fat mass were assessed with dual‐energy X‐ray absorptiometry. Endocrine factors were assessed from fasted venous blood samples. We applied the novel four‐way decomposition method to analyze associations between fat mass, endocrine factors, and BMC, adjusting for age, stature, pubertal status, lean mass, and baseline BMC. The four‐way decomposition method allows the total effect of an exposure on an outcome to be decomposed into a controlled direct effect, a reference interaction, a mediated interaction, and a pure indirect effect.

**Results**: Fat mass had a positive controlled direct effect on BMC in girls and boys (β = 0.033 to 0.68, *p* < 0.001). We observed a negative interaction between fat mass and adiponectin with BMC in girls (β = −0.003, *p* = 0.033), and a negative mediated interaction between fat mass and free leptin index with BMC in boys (β = −0.007, *p* = 0.007).

**Conclusions**: In children with greater levels of adiponectin and free leptin index, the relationship between fat mass and BMC became less positive, in girls and boys, respectively. Fat mass primarily positively influenced BMC through pathways not related to the endocrine factors we assessed, likely through mechanical loading. Because the relationship between fat mass and endocrine factors with BMC is likely moderated by weight status and pubertal stage, further research is needed to assess whether these observations extend to children and adolescents with overweight and obese weight status.

**Figure:**
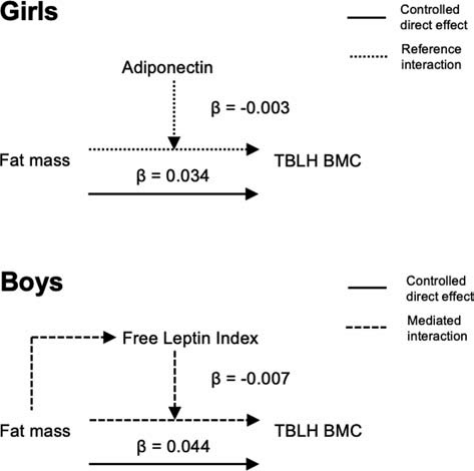
Figure 1. Summary of significant mediation and moderation effects from the 4‐way decomposition, adjusted for age, stature, pubertal status, lean mass, and baseline BMC. Girls: n = 191, Boys: n = 181.

## The epidemiology of osteoporosis and sarcopenia in a high HIV prevalence setting in rural South Africa: a cross‐sectional study

### Celia Gregson ^1,2^, Tafadzwa Madanhire^3,2^, Andrea Rehman^4^, Rashida Ferrand^4,3^, Anne Cappola^5^, Steven Tollman^2^, Tshepiso Mokoena^2^, The ARK Consortium^2^, Lisa Micklesfield^2^, Alisha Wade^2^, June Fabian^2^

#### ^*1*^ *University* *of Bristol, Bristol, UK.* ^*2*^ *University* *of the Witwatersrand, Johannesburg, South Africa.* ^*3*^ *Biomedical* *Research and Training Institute, Harare, Zimbabwe.* ^*4*^ *London* *School of Hygiene and Tropical Medicine, London, UK.* ^*5*^ *University* *of Pennsylvania, Pennsylvania,* *USA*

**Abstract**

**Background:** An estimated 17.2% of older adults are living with HIV in South Africa. With increased access to treatment, life expectancy is rising across the region. However, age‐related diseases, eg, osteoporosis and sarcopenia, remain poorly characterized. We aimed to determine the prevalence of osteoporosis, sarcopenia and osteosarcopenia, and understand relationships between HIV, BMD, muscle strength, mass, and gait speed.

**Methods:** A cross‐sectional study of men and women age 20–80 years resident in a health and sociodemographic surveillance site in rural South Africa, collected demographic and clinical data, including HIV status, and measured grip strength, gait speed, body composition, and BMD by DXA. Probable and confirmed sarcopenia were defined by European (EWGSOP2) guidelines, osteoporosis by BMD *T*‐score ≤ −2.5 (if age ≥50 years), and osteosarcopenia as probable or confirmed sarcopenia plus osteoporosis. Multivariate linear regression assessed relationships between HIV and components of sarcopenia and BMD outcomes.

**Results:** The mean ± SD age of 805 participants was 44.6 ± 14.8 years, 547 (68.2%) were female, of whom 187 (34.2%) were postmenopausal; 34 (13.2%) men and 129 (23.6%) women had HIV. A femoral neck *T*‐score ≤ −2.5, seen in four of 95 (4.2%) men and 39 of 201 (19.4%) women age ≥50 years, was more common in women with than without HIV (13/35 [37.1%] versus 26/166 [15.7%]; *p* = 0.003). Although no individual had confirmed sarcopenia, probable sarcopenia was seen in more men than women (30/258 [11.6%] versus 24/547 [4.4%]; *p* = 0.001). Osteosarcopenia was rare, affecting just 2%. Although appendicular skeletal mass (ASM)/height^2^ was lower in both men and women with HIV, there were no differences in grip strength, gait speed, or probable sarcopenia by HIV status. In multivariate analyses older age, female sex, lower ASM/height^2^, slower gait speed, and HIV infection were all independently associated with lower femoral neck BMD. In women, an interaction was detected between HIV and menopause on total body less‐head (TB‐LH) and total hip BMD (interaction *p* values 0.01 and 0.05, respectively), such that of 187 postmenopausal women, the 31 with HIV had 0.035 (95% confidence interval [CI], 0.004–0.065) g/cm^2^ lower TB‐LH BMD and 0.056 (95% CI, 0.008–0.104) g/cm^2^ lower total hip BMD than the 156 without HIV.

**Conclusion:** Osteoporosis rather than sarcopenia is the common musculoskeletal disease of aging in rural South Africa; postmenopausal women with HIV may experience greater bone losses than women without HIV. Findings raise concerns over future age‐related fracture risk in Southern Africa, and suggest HIV clinics in South Africa should consider routine bone health assessment, particularly in postmenopausal women.

## The impact of adiposity and lean mass on bone health and metabolic profile in Asian women of reproductive age

### Mya Thway Tint ^1,2^, Andrea Cremaschi^1^, Jerry Kok Yen Chan^3^, Lyenette Pei Chi Shek^2^, Peter Gluckman^4^, Yap‐Seng Chong^1,2^, Keith Godfrey^5^, David Cameron‐Smith^1^ , Maria De lorio^1,2^, Shiao‐Yng Chan^1,2^, Johan Eriksson^1,2,6,7^

#### ^*1*^ *Singapore* *Institute of Clinical Sciences, Singapore, Singapore.* ^*2*^ *Yong* *Loo Lin School of Medicine, National University of Singapore, Singapore, Singapore.* ^*3*^ *KK* *Women's and Children's Hospital, Singapore, Singapore.* ^*4*^ *Liggins* *Institute, Auckland, New Zealand.* ^*5*^ *University* *of Southampton, Southampton, UK.* ^*6*^ *University* *of Helsinki, Helsinki, Finland.* ^*7*^ *Folkhalsan* *, Helsinki, Finland*

**Abstract**

**Background and Objectives:** It is increasingly recognized that the risk of fractures is increased in individuals with type 2 diabetes, challenging the traditional view of a positive influence of adiposity on bone mineral density (BMD). Obesity increases fat deposition in muscle tissue and production of inflammatory cytokines. These cytokines can interfere with bone resorption and bone formation, influencing overall bone health negatively. These findings underscore the complex interrelationships between fat mass (FM), lean mass (LM), and bone health in obesity and metabolic diseases. Women tend to have higher degree of adiposity, lose bone earlier and faster compared to men. We therefore investigated the impact of LM, FM, and metabolic profile on bone health in Asian women of reproductive age.

**Methods:** A total of 191 Asian women of reproductive age from the Singapore Preconception Study of Long‐Term Mother and Child Outcomes (S‐PRESTO) were studied. BMD of femoral neck (BMD_FN_), lumbar spine (BMD_LS_), whole body (BMD_WB_), LM, and FM were measured by dual‐energy X‐ray absorptiometry (DXA) scans. Metabolic markers fasting plasma glucose (FPG), 2‐hour plasma glucose (2‐hrPG), fasting plasma insulin (FPI), blood lipid concentrations, and high‐sensitivity C‐reactive protein (HS‐CRP) were measured. Homeostatic assessment of insulin resistance (HOMA‐IR) was calculated as (FPI [mU/L] * FPG [mmol/L])/22.5. Multivariable regression analyses were performed to determine the associations of LM, FM, and metabolic markers with BMD.

**Results:** Women were between 24 to 45 years (mean ± SD) 32 ± 4 years and had a BMI between 15.5 to 43.3 kg/m^2^ (mean ± SD) 23.5 ± 4.8 kg/m^2^). Adjusting for potential confounders (ethnicity, age, age at menarche, height, education, vitamin D concentrations, physical activity), BMI had positive associations with BMD_FN_, BMD_LS_, and BMD_WB_. We further investigated the individual contribution of LM and FM on BMD. LM had a positive association with BMD_FN_, BMD_LS_, and BMD_WB_ (Table 1). However, higher FM was associated with lower BMD_WB_. Moreover, FPG, 2‐hrPG, HOMA‐IR showed inverse relationships with BMD_FN_, BMD_LS_ and/or BMD_WB_ independent of level of adiposity (Fig. 1). Higher concentrations of high density lipoprotein were associated with higher BMD_WB._

**Conclusion:** LM but not FM was a strong determinant of BMD in Asian women of reproductive age. Our findings suggest that maintaining a healthy muscle mass and favorable metabolic profiles have beneficial effects on bone health and might lower the risk for osteoporosis. Future studies to examine modifiable risk factors and underlying complex mechanisms between LM/FM and bone mass are warranted.

## Bone loss and osteoporosis in older adults in rural Gambia: findings from the Gambian Bone and Muscle Ageing Study (GamBAS)

### Camille Parsons^1^, Mícheál Ó Breasail^2^, Landing Jarjou^3^, Ayse Zengin^4^, Ann Prentice^3^, Peter Ebeling^4^, Cyrus Cooper^1,5^, Kate Ward ^1,2,3^

#### ^*1*^ *MRC* *Lifecourse Epidemiology Unit, University of Southampton, Southampton, UK.* ^*2*^ *MRC* *Nutrition and Bone Health Group, Cambridge, UK.* ^*3*^ *MRC* *Unit The Gambia at the London School of Hygiene and Tropical Medicine, Banjul, Gambia.* ^*4*^ *Department* *of Medicine, School of Clinical Sciences, Faculty of Medicine, Nursing and Health Sciences, Monash University, Victoria, Australia.* ^*5*^ *National* *Institute for Health Research (* *NIHR* *) Musculoskeletal Biomedical Research Unit, University of Oxford, Oxford, UK*

**Abstract**

**Introduction:** Due to an increase in longevity and rapid urbanization in sub‐Saharan Africa (SSA), it is predicted that the incidence of hip fracture will increase sixfold in Africa and Asia by 2050. To date there are no longitudinal studies focusing on musculoskeletal aging in SSA. The aims were to describe and characterize changes in bone mass of older adults and to determine prevalence of osteoporosis and osteopenia in the population.

**Methods:** The Gambian Bone and Muscle Aging Study (GamBAS; ISRCTN17900679) is a prospective observational study in women and men from rural Gambia. Participants were recruited into sex‐stratified, 5‐year age bands. The primary outcome was change in total hip areal bone mineral density (aBMD) over a 1.5‐year to 2‐year period (follow‐up was randomized to account for seasonality). A GE‐Lunar Prodigy scanner was used to obtain proximal femur scans for assessment of aBMD. Annualized percentage change in aBMD was calculated. Total hip BMD *T*‐scores were calculated using National Health and Nutrition Examination Survey (NHANES) data and *Z*‐scores were calculated using manufacturer reference data as per International Society for Clinical Densitometry (ISCD) recommendations. Results are presented as mean ± standard deviation (SD) change.

**Results:** A total of 383 (54.6% women) had repeat dual‐energy X‐ray absorptiometry (DXA) scans (median follow‐up 1.72 years); median age 59 years; range, 40–92 years; body mass index (BMI) men 20.9 ± 3.0 kg/m^2^; women 21.9 ± 3.5 kg/m^2^. At baseline, the mean total hip *Z*‐score was −0.23 ± 0.98 in men and −0.55 ± 1.06 in women. The mean total hip *T*‐score in men was 0.23 ± 1.10 and 1.25 (1.42) in women. Forty‐two (20.6%) women, and one (0.6%) man had osteoporosis, and *n* = 82 (40.2%) women and 25 (14.5%) men had osteopenia. Figure 1 shows the mean annualized percentage change in total hip aBMD.

In men, total hip aBMD changed most after the age of 70 years: 70–74 years −1.10 ± 1.20, and 75+ years −1.24 ± 0.98. In women, the change in total hip a BMD change was −0.17 ± 1.19 in 40–44 years; −1.09 ± 1.46 in 45–49 years; −1.22 ± 1.23 in 50–54 years; −1.22 ± 1.27 in 55–59 years; −0.73 ± 1.31 in 60–64 years; −1.22 ± 1.04 in 65–69 years; −1.00 ± 1.36 in 70–74 years; and −1.50 ± 1.35 in 75+ years.

**Conclusions:** These are the first longitudinal musculoskeletal DXA data in an older‐adult population from SSA. The change at the hip was comparative to that in higher‐income countries where fracture risk is high. Over 40% of women were osteopenic and osteoporotic. These data provide important insights into musculoskeletal health in The Gambian population, which was previously thought to be at low risk of fracture.

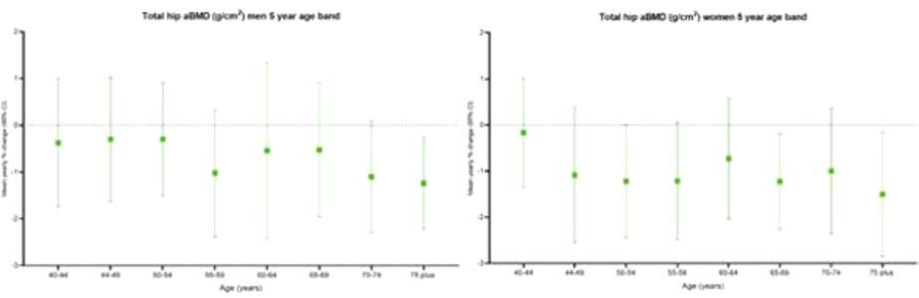

## Associations between inflammatory status and bone phenotype: Results from the MRC National Survey of Health and Development

### Ruth Durdin ^1^, Camille Parsons^1^, Diana Kuh^2^, Elaine M. Dennison^1^, Rachel Cooper^3^, Cyrus Cooper^1^, Kate A Ward^1^

#### ^*1*^ *MRC* *Lifecourse Epidemiology Unit, University of Southampton, Southampton, UK.* ^*2*^ *MRC* *Unit for Lifelong Health and Ageing, University College London, London, UK.* ^*3*^ *Manchester* *Metropolitan University, Manchester, UK*

**Abstract**

**Background:** Osteoporosis is of concern given its association with fragility fractures. Adipose tissue, a source of inflammatory markers and adipokines, is a potentially important determinant of poorer bone health.

**Aims:** The aim was to investigate associations between interleukin‐6 (IL‐6), adiponectin, and leptin with dual‐energy X‐ray absorptiometry (DXA) and peripheral quantitative computed tomography (pQCT)‐measured bone phenotypes at age 60–64 years.

**Methods:** The MRC National Survey of Health and Development (NSHD) is a prospective birth cohort study; 766 men and 820 women included in these cross‐sectional analyses had DXA (total hip, spine, whole body areal BMD [aBMD]) and pQCT (radius: 4% and 50% sites) measurements. Multiple linear regression, stratified by sex, was used to determine associations between inflammatory markers (IL‐6, adiponectin, and leptin) and bone adjusted for fat mass residuals (the residual of fat mass after adjustment for lean mass and height). Results are presented as standardized β (95% confidence interval [CI]) *p* value; β is change in aBMD or volumetric BMD (vBMD) in standard deviations per unit increase in inflammatory marker.

**Results:** In men, after adjustment for fat mass residuals, log(IL‐6) was negatively associated with total hip (−0.12 [95% CI, −0.23 to −0.02], *p* = 0.02) and whole body (−0.15 [95% CI, −0.25 to −0.04], *p* = 0.01) aBMD; in women, associations did not remain after adjustment (total hip: 0.03 [95% CI, −0.07 to 0.13] *p* = 0.56; whole body: 0.06 [95% CI, −0.05 to 0.16], *p* = 0.29). In men, log(IL‐6) was negatively associated with total vBMD (men: −0.13 [95% CI, −0.25 to −0.01], *p* = 0.03) and in women there was a positive association (0.14 [95% CI, 0.03–0.26], *p* = 0.02). After adjustment, associations between log(leptin) and aBMD remained only at the total hip (men: 0.13 [95% CI, 0.01–0.25], *p* = 0.03; women: 0.24 [95% CI, 0.13–0.35], *p* < 0.01); log(leptin) was also associated with trabecular vBMD in men and women (men: 0.18 [95% CI, 0.04–0.32], *p* = 0.01; women: 0.20 [95% CI, 0.07–0.34], *p* < 0.01). The association between log(leptin) and cortical vBMD remained in women only (0.17 [95% CI, 0.04–0.30], *p* = 0.01). Adiponectin was negatively associated with aBMD, at all sites (whole body: men: −0.01 [95% CI, −0.02 to −0.0002], *p* = 0.02; women: −0.01 [95% CI, −0.02 to −0.003], *p* = 0.01), as well as with trabecular vBMD, in men and women. Associations between adiponectin and cortical and total vBMD existed in women only (cortical: −0.01 [95% CI, −0.02 to −0.003], *p* = 0.01; total: −0.02 [95% CI, −0.03to −0.01], *p* < 0.01).

**Conclusions:** Overall, these results highlight differing associations between markers of inflammation and adiposity with bone, which might influence the aging process. Although sex interactions were not tested, these results suggest that some associations may differ in men and women.

## Epigenetic age acceleration associations with skeletal outcomes: Differential impacts in men and women

### Nicholas Fuggle ^1^, Michael Clynes^1^, Micheal Ó Breasail^2^, Camille Parsons^1^, John Holloway^3^, Negusse Kitaba^3^, Kate Ward^1^, Cyrus Cooper^1^, Elaine Dennison^1^

#### ^*1*^ *MRC* *Lifecourse Epidemiology Unit, Southampton, UK.* ^*2*^ *University* *of Bristol, Bristol, UK.* ^*3*^ *University* *of Southampton, Southampton, UK*

**Abstract**

**Background:** Osteoporosis is traditionally considered a condition of aging. Epigenetic clocks are composed of a selection of CpG sites which have the potential to capture “biological age” and provide a measure of age acceleration (calculated as the difference between biological and chronological age). Here we investigate the associations between age acceleration (according to three different clocks: HorvathAge, GrimAge, and PhenoAge) and hip dual‐energy X‐ray absorptiometry (DXA) parameters.

**Methods:** Participants were recruited across three generations of the Hertfordshire Intergenerational Study; original cohort members, their children, and grandchildren. Questionnaires were used to capture demographic and lifestyle information and participants attended research clinics at which height (m) and weight (kg) were measured and hip DXA was performed (Lunar iDXA; GE Healthcare). Blood samples were collected and DNA methylation analyzed using the Illumina 850 k array (Infinium MethylationEPIC BeadChip). After preprocessing (using the *meffil* package) GrimAge, PhenoAge, and HorvathAge acceleration were calculated. Associations with DXA hip measures (including bone mineral density [BMD], bone mineral content [BMC], and bone area) were analyzed using linear regression in sex‐stratified unadjusted models and those adjusted for age and body mass index (BMI). Results are presented as β coefficients with 95% confidence intervals (CIs).

**Results:** A total of 114 participants (39 males and 75 females) were recruited, mean age 56 years (range, 18 to 88 years). Relationships varied between clocks; HorvathAge acceleration was not associated with DXA measures in any models. However, greater GrimAge acceleration was associated with significantly lower hip BMC (β = −0.94 [95% CI, −1.50 to −0.38], *p* < 0.01 and lower bone area (β = −0.28 [95% CI, −0.55 to −0.01], *p* < 0.05) in males in fully adjusted models, and with lower hip BMD in males in unadjusted models (β = −0.02 [95% CI, −0.04 to −0.01], *p* < 0.05). No significant associations with GrimAge were observed in females. Greater PhenoAge acceleration was associated with lower hip BMC in males in models adjusted for age and BMI (β = −0.34 [95% CI, −0.65 to −0.03], *p* < 0.05) and lower hip BMD in males in unadjusted models only (β = −0.01 [95% CI, −0.02 to −0.00], *p* < 0.05). In females, greater PhenoAge acceleration was associated with greater hip BMD (β = 0.01 [95% CI, 0.00–0.01], *p* < 0.05) and BMC (β = 0.26 [95% CI, 0.02–0.50], *p* < 0.05), in unadjusted models alone.

**Conclusions:** Age acceleration according to newer iterations of epigenetic clocks (GrimAge and PhenoAge) which were designed to measure age‐related phenotypic changes were associated with bone measures at the hip in males, whereas the first‐generation clock (HorvathAge) was not. The sex‐specific associations and the unexpected trend for accelerated biological aging associated with greater bone outcomes in females, require further investigation.

## Incidence and number of hip fractures in South Africa: estimated projections from 2020 to 2050

### Samuel Hawley ^1^, Sapna Dela^2^, Anya Burton^1^, Farhanah Paruk^3^, Bilkish Cassim^3^, Celia Gregson^1^

#### ^*1*^ *Musculoskeletal* *Research Unit, Translational Health Sciences, Bristol Medical School, University of Bristol, Bristol, UK.* ^*2*^ *Department* *of Internal Medicine, Edendale Hospital, Pietermaritzburg, South Africa.* ^*3*^ *Department* *of Rheumatology, School of Clinical Medicine, University of Kwa* *Zulu‐Natal* *, Pietermaritzburg, South Africa*

**Abstract**

**Background:** Hip fracture is an established major public health problem among older adults in high‐income settings; however, data from the sub‐Saharan African region are scarce. Yet, this century, the number of older adults in sub‐Saharan Africa is expected to grow faster than any other region globally. We aimed to use emerging data on hip fracture incidence in South Africa to estimate future burden of hip fracture for the country over the next three decades.

**Methods:** Previously collected data on hip fracture patients from eight districts within the Gauteng, KwaZulu‐Natal, and Western Cape regions of South Africa were re‐analyzed. All patients aged ≥40 years with a radiograph‐confirmed hip fracture over a 12‐month period in one of 94 included hospitals were enrolled. High‐velocity trauma, pathological and periprosthetic fractures were excluded. Age‐, sex‐ and ethnicity‐specific incidence rates were generated and standardized to the 2011 South African census population and to future South African population projections estimated by the United Nations (UN). A correction factor was applied to UN projections for the population size aged ≥80 years, derived from the underestimated 2011 UN population size compared to the South African 2011 census.

**Results:** The 2767 included hip fracture patients had mean ± standard deviation (SD) age 73.7 ± 12.7 years; 69% were female. Incidence rates (per 100,000 people), standardized to the estimated South African population in 2020, were 104 for females and 47 for males. Rates for Black Africans (the largest ethnic group in South Africa; 79.2% of total population) were lower at 63 for females and 40 for males. Overall projected incidence rates were discernibly higher by the year 2040 (122 and 53 for females and males, respectively) and increased further by the year 2050 (141 and 60 for females and males, respectively). In terms of the overall annual number of hip fractures for the country, estimates increased from approximately 10,000 in 2020 to approximately 23,000 by 2050 (approximately 16,500 in Black Africans and approximately 6500 in other ethnic groups). The overall and age‐stratified number of hip fractures are shown in the figure.

**Conclusion:** The hip fracture burden for South Africa, whose last census population was 52 million, is estimated to more than double over the next 30 years, to approximately one‐third of those currently seen in the UK. Significant investment in fracture prevention services and inpatient fracture care is likely to be needed to meet this demand.

**Acknowledgments:** NIHR‐Wellcome Partnership for Global Health Research (217135/Z/19/Z).

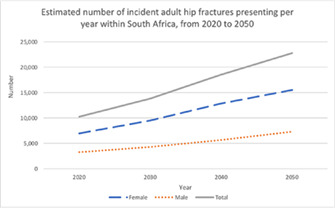

## Frailty is associated with inflammation and poorer bone mineral density independent of body mass index: findings from UK Biobank

### Elizabeth Curtis ^1^, Stefania D'Angelo^1^, Stephen Woolford^1^, Ruth Durdin^1^, Zahra Raisi‐Estabragh^2^ ^,3^, Kate Ward^1^, Cyrus Cooper^1,4,5^, Nicholas Harvey^1,4^

#### ^*1*^ *MRC* *Lifecourse Epidemiology Unit, University of Southampton, Southampton, UK.* ^*2*^ *William* *Harvey Research Institute,* *NIHR* *Barts Biomedical Research Centre, Queen Mary University of London, London, UK.* ^*3*^ *Barts* *Heart Centre, St Bartholomew′s Hospital, Barts Health* *NHS* *Trust, London, UK.* ^*4*^ *NIHR* *Southampton Biomedical Research Centre, University of Southampton and University Hospital Southampton* *NHS* *Foundation Trust, Southampton, UK.* ^*5*^ *NIHR* *Biomedical Research Centre, University of Oxford, Oxford, UK*

**Abstract**

**Background:** Frailty, a common syndrome associated with aging, represents a huge public health burden. It is associated with fundamental processes of aging such as chronic inflammation but the independence of these relationships from age, sex, lifestyle, and adiposity is not clear. We therefore investigated in UK Biobank, associations between frailty, blood biomarkers, and bone health, independent of these characteristics.

**Methods:** A total of 502,640 participants aged 40–69 years at baseline were recruited to UK Biobank 2006–2010. Venous blood samples were obtained. From 2014 onward, a subset underwent an imaging follow‐up visit, which included whole‐body dual‐energy X‐ray absorptiometry (DXA) (GE Lunar iDXA), grip strength measurement (Jamar dynamometer), and a questionnaire. Frailty was defined using a modification of Fried's classification (at least three of weight loss, mental exhaustion, low physical activity, slow gait speed, and low grip strength). The presence of one to two criteria designated pre‐frailty. Linear regression was used to discern associations between frailty status, biochemical markers (C‐reactive protein [CRP], 25(OH)‐vitamin D, glycated hemoglobin [HbA1c]) and bone outcomes, adjusting for age, sex, body mass index (BMI), smoking, alcohol, and educational level. Non‐frail was the reference category and blood biomarkers were standardized with the beta coefficient representing the mean difference in standard deviation (SD).

**Results:** A total of 22,332 participants (11,484 women and 10,848 men) with frailty assessment and either DXA bone measures or blood biochemistry were included in the analysis; of these 547 (2.4%) were frail and 9359 (41.9%) were pre‐frail. Frail participants were more likely to be female (59.6% versus 50.9%), older (mean ± SD, 63.2 ± 7.9 versus 62.6 ± 7.3 years), and of higher BMI (mean ± SD, 30.7 ± 6.4 versus 25.9 ± 4.0 kg/m^2^). After full adjustment, frail participants had higher CRP (+0.29 SD; 95% confidence interval [CI], 0.21–0.37]), lower 25(OH)‐vitamin D levels (−0.36 SD [95% CI, −0.45 to −0.27]), and higher HbA1c (+0.32 SD [95% CI, 0.23–0.40]), all *p* < 0.001. Frail participants also had lower femoral neck (−0.02 g/cm^2^ [95% CI, −0.03 to −0.01], *p* = 0.002) and lumbar spine bone mineral density (BMD) (−0.02 g/cm^2^ [95% CI, −0.03 to −0.01], *p* = 0.005). BMD associations were only apparent after adjustment for BMI. Similar associations were observed for pre‐frail versus non‐frail participants.

**Discussion:** Frailty is associated with greater levels of systemic inflammation, lower 25(OH)‐vitamin D, poorer glucose handling, and lower BMD, independent of age, sex, lifestyle, and BMI. These findings suggest that frailty associations with age‐associated inflammation (inflammaging) are only partly mediated via adiposity and warrant further mechanistic investigation.

## Imaging

## The assessment of mechanostructural properties in the femoral neck sampled from obese cadaveric men: a pilot study

### Jelena Jadzic ^1^, Petar Milovanovic^1^, Marija Djuric^1^, Vladimir Zivkovic^2^, Slobodan Nikolic^2^, Danijela Djonic^1^

#### ^*1*^ *Laboratory* *for Anthropology and Skeletal biology, Institute for Anatomy, Faculty of Medicine, University of Belgrade, Belgrade, Serbia.* ^*2*^ *Institute* *of Forensic Medicine, Faculty of Medicine, University of Belgrade, Belgrade, Serbia*

**Abstract**

Despite growing evidence for the detrimental effects of obesity on bone strength, health professionals worldwide cannot precisely assess the site‐specific fracture risk in obese individuals. Having in mind the significance and severity of femoral fractures in the general population, this pilot study aimed to compare microstructural and mechanical properties of the femoral neck sampled from obese donors (body mass index [BMI] >30 kg/m^2^) and normal‐weight sex‐matched controls (BMI between 20 and 25 kg/m^2^). General exclusion criteria for the study participants were a positive history of endocrine, metabolic, and cancerous diseases affecting the skeletal system, data on the previous femoral fractures, permanent immobility, alcohol consumption, and the use of bone affecting therapy. After the Institutional Ethics Committee approved the sample collection, we performed micro–computed tomography (μCT) and Vickers microhardness analysis of superolateral and inferomedial femoral neck samples (Figure), divided into obese (*n* = 10, age: 49 ± 16 years) and control group (*n* = 10, age: 47 ± 20 years).

Our data suggest better quality of trabecular and cortical microstructure of the superolateral (trabecular number [Tb.N] 1.085 ± 0.166/mm versus 0.951 ± 0.134/mm; cortical porosity [Ct.Po] 14.581 ± 2.571% versus 21.377 ± 7.859%; cortical thickness [Ct.Th] 3.554 ± 0.365 mm versus 3.055 ± 0.811 mm) and inferomedial femoral neck (bone volume/total volume [BV/TV] 29.338 ± 6.707% versus 23.400 ± 4.436%; trabecular thickness [Tb.Th] 0.247 ± 0.022 mm versus 0.224 ± 0.032 mm; Ct.Po 5.610 ± 1.320% versus 9.720 ± 3.005%; Ct.Th 6.7 ± 1.1 mm versus 5.2 ± 1.5 mm; pore diameter [Po.Dm] 0.131 ± 0.036 mm versus 0.186 ± 0.035 mm) in obese donors than in healthy controls (*p* < 0.05, Student's *t* test). Still, we did not demonstrate significant difference in cortical Vickers hardness (VH) values of the superolateral (76.9 ± 6.0 VH versus 71.8 ± 7.3 VH, *p* = 0.106) and inferomedial portion of the femoral neck (76.2 ± 8.1 VH versus 71.4 ± 6.6 VH, *p* = 0.163) sampled from obese, when compared to control individuals.

In conclusion, obese individuals had thicker and less porous cortex of the superolateral and inferomedial femoral neck samples, compared to their normal‐weight counterparts. Additionally, greater trabecular density in the inferomedial neck was caused by increased trabecular thickness in obese men. These microarchitectural changes are likely associated with continuous mechanical load due to increased body weight in obese men. Still, cortical Vickers microhardness of the femoral neck samples was not significantly more favorable in obese individuals. Thus, the additional multi‐scale bone quality assessment could be required to give better insights into fracture development among obese men.

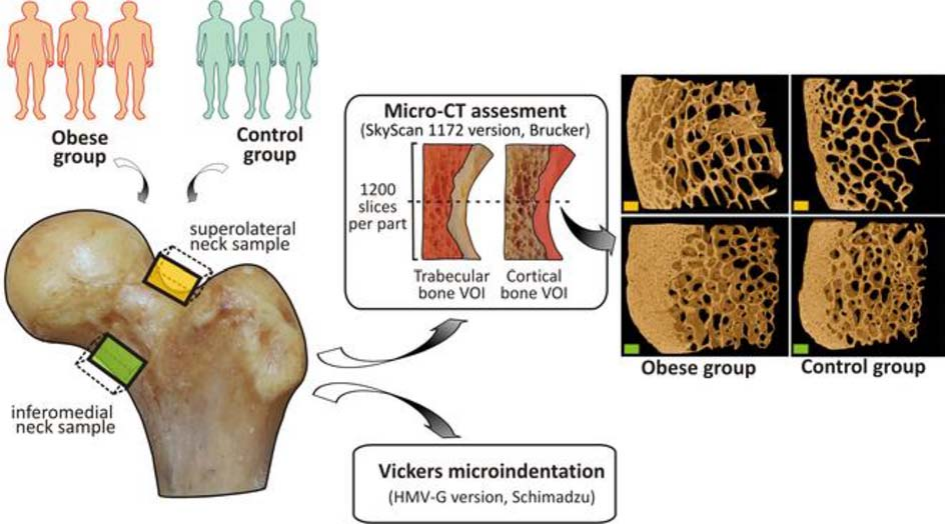

## Tumors calcinosis in a hemodialysis patients: report of two cases

### Kawtar Nassar, Saadia Janani

#### *Department of Rheumatology, Ibn Rochd University Hospital Center, Hassan* *II* *University, Faculty of Medicine and Pharmacy. Casablanca, Casablanca, Morocco*

**Abstract**

**Background:** Tumor calcinosis (CT) is a rare condition characterized by the calcium material deposition in extra‐articular soft tissues taking a tumor form. It can be primary or secondary. Chronic renal failure is the main cause of metastatic calcifications. Different factors predispose the dialysis patient to calcium salt deposits. We report two cases of tumors calcinosis in a hemodialysis patients.

**Case reports**

**Case 1:** A 61‐year‐old‐patient who is followed for hypertensive nephropathy for which he is under periodic hemodialysis. He consults for mechanical pain in the upper extremity of the right leg and both shoulders. Standard radiographs revealed polycyclic, heterogeneous formations, occupying the periarticular space of the two shoulders, especially on the right one. In the right knee, there is a multiple calcifications of the superior and postero‐external soft tissue. The magnetic resonance imaging (MRI) notes multiple images of macro‐calcifications (Fig. 1). The biological assessment found calcemia at 103 mg/L, hyperphosphatemia at 71 mg/L, hyperparathyroidism at 128.50 pg/mL, and uric acid at 69 mg/L.

**Case 2:** A 53‐year‐old man in hemodialysis for 6 years. He consults for mechanical knee pain. Physical exam found a 6‐cm swelling at the latero‐extremity of the left knee. Radiograph and ultrasound revealed polycyclic and heterogenous formations retrocondylar (Fig. 2). Biological exam found hyperphosphoremia at 60 mg/L, calcium at 110 mg/L, and hyperparathyroidism at 300 pg/mL.

**Discussion:** Different factors predispose the dialysis patient to calcium salt deposits: hyperparathyroidism, elevation of phosphocalcic product, excess intake of calcium or vitamin D, poor compliance of the patient with regular intake of chelators, insufficient dialysis, or a dialysate rich in aluminum.

The clinical context and the radiological data made the diagnosis of pseudotumoral calcinosis in our patients with chronic dialysis. The management is based on the correction of the Ca/ph product. The reduction of calcifications go through the correction of hyperparathyroidism or the increase of dialysis sessions with low‐Ca dialysate, while checking the aluminum level in the dialysate. Kidney transplantation can promote rapid decrease in calcifications and is desirable in cases of severe calcinosis. Surgery with as complete a resection as possible is recommended when there is a risk of vascular damage or on joint mobility.

## Sexual dimorphism in cortical vascular canals during arduous military training

### Lysanne V. Michels ^1^, Alice Goring^1^, Jacob Trend^1^, Andrew A Pitsillides^2^, Philipp Schneider^1^, Thomas J O'Leary^3,4^, Claire E Clarkin^1^, Julie P Greeves^3,4,5^

#### ^*1*^ *University* *of Southampton, Southampton, UK.* ^*2*^ *Royal* *Veterinary College, London, UK.* ^*3*^ *Army* *Health and Performance Research, Andover, UK.* ^*4*^ *University* *College London, London, UK.* ^*5*^ *University* *of East Anglia, Norwich, UK*

**Abstract**

The incidence of bone stress injuries is higher in women than in men during arduous military training (O'Leary et al.^(1)^).The human bone cortex is penetrated by blood vessels via the Haversian and Volkmann canals, and this cortical porosity might be involved in the development of stress fractures. We have recently reported sexual dimorphism in blood vessel function of the murine bone cortex, with vascular signaling showing direct links to bone mineralization and strength via sex‐specific gene expression (Goring et al.^(2)^). Herein, we aimed to identify whether sexual dimorphism in the cortical vasculature exists in humans during arduous military training. We have undertaken high‐resolution peripheral quantitative computed tomography imaging of age‐matched male (*n* = 5) and female (*n* = 6) British Army Officers during 1, 14, 28, and 44 weeks of training. These diaphyseal tibial scans with a voxel size of 61 μm were imported into ImageJ, where intracortical blood vessel canals were manually separated from trabecular pores to extract cortical porosity (Ct.Po), cortical bone tissue volume (Ct.TV), canal volume (Ca.V), canal diameter (Ca.Dm), and canal density (Ca.Dn). Significant sexual divergence in cortical parameters was detected from baseline: Ct.Po (*p* < 0.05), Ct.TV (*p* < 0.001), and Ca.Dn (*p* < 0.005) were higher, Ca.V was similar (*p* > 0.05), and Ca.Dm was lower (*p* < 0.001) in men than women across all time points (*p* > 0.05 for exercise in every parameter). Men therefore have more and narrower blood vessel canals than women, who have fewer but wider canals. Our results indicate that regulation of the cortical vasculature may be sex‐specific, which could contribute to increased stress fracture risk in women compared with men. These findings can aid future development of exercise regimes targeting bone injury prevention in both sexes.

**References**

1. O'Leary TJ, Wardle SL, Gifford RM, et al. (2021). Tibial macrostructure and microarchitecture adaptations in women during 44‐weeks of arduous military training. *J Bone Miner Res*. 2021;36(7):1300‐1315. https://doi.org/10.1002/jbmr.4290.

2. Goring A, Sharma A, Javaheri B, et al. Regulation of the bone vascular network is sexually dimorphic. *J Bone Miner Res*. 2019;34(11):2117‐2132. https://doi.org/10.1002/jbmr.3825.

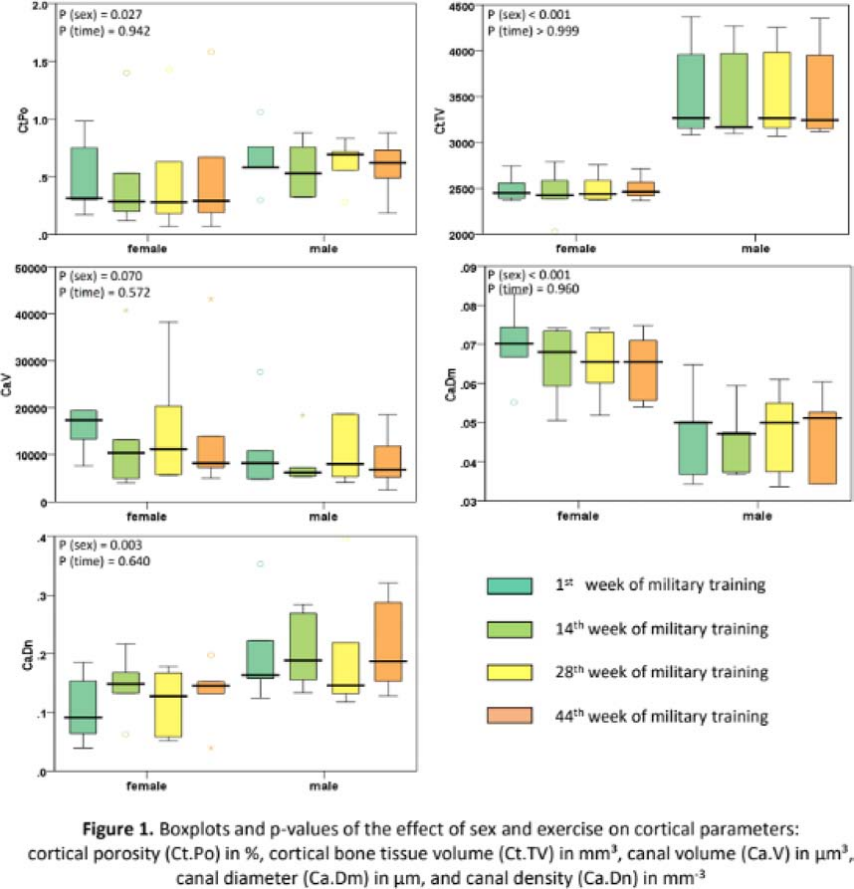

## Prospects of multimodal nonlinear microscopy for research and diagnosis of osteosarcoma

### Belle Creith, Peter Johnson, James Harrison, Richard Oreffo, Sumeet Mahajan, Claire Clarkin

#### University Of Southampton, Southampton, UK

**Abstract**

**Introduction:** Osteosarcoma (OS) is the most common primary cancer of bone, typically describing highly aggressive pediatric tumors characterized by an aberrant, immature, extracellular matrix (ECM) (Shapiro^(1)^; Shapiro and Eyre^(2)^). The manipulated matrix of OS is believed to drive malignant progression, though mechanisms for which are yet to be concluded (Cui et al.^(3)^). In recent years, novel approaches such as nonlinear microscopy have been utilized in the field of oncology for rapid, noninvasive detection of tumors—via examination of the ECM—as well as uncovering potential therapeutic targets (Nadiarnykh et al.^(4)^; Provenzano et al.^(5)^). Herein, we aim to investigate the potential of multimodal, nonlinear microscopy for distinction of OS from healthy bone.

**Method:** Multimodal, nonlinear microscopy—including second harmonic generation (SHG) imaging and two‐photon excitation fluorescence (TPEF)—was employed to examine decalcified and paraffin‐embedded human bone and OS tissue sections. The imaging protocol was optimized on human bone biopsies (*n* = 3 male *n* = 3 female). Image analysis by ImageJ and CT‐FIRE–enabled quantification of matrix‐collagen parameters including fiber length, width, angle, straightness, and number.

**Results:** In nondiseased cortical bone, several matrix collagen parameters were quantified including: average fiber length (27.48 ± 1.22 mm, 28.08 ± 1.32 mm), average fiber width (2.20 ± 0.017 mm, 2.19 ± 0.062 mm), average fiber angle (90.44° ± 1.69 degrees, 87.59° ± 2.4 degrees) and average fiber straightness (0.92 ± 0.0025, 0.92 ± 0.0017) for male and female bone sections, respectively. Preliminary data from human OS tissue revealed that both collagen fiber length (31.08 ± 1.21 mm) and collagen fiber number (387.25 ± 10.22) were particularly distinctive between normal bone (Fig. 1A,B) and OS tissue (Fig. 1C,D).

**Conclusion:** Further investigations into matrix disparities between normal bone and various malignancy grades of OS will provide clearer insight into potential diagnostic signatures that may be used in clinical settings for OS diagnosis. This preliminary data highlights the promising potential of nonlinear microscopy for research and diagnosis of OS. Our continued work aims to utilize nonlinear, optical modalities for in‐depth characterization of the unique OS matrix and elucidate a potential diagnostic signature that may be used for noninvasive OS examination in clinical settings.

**References**

1. Shapiro F. Ultrastructural observations on osteosarcoma tissue: a study of 10 cases. *Ultrastruct Pathol*. 1983;4(2‐3):151‐161. https://doi.org/10.3109/01913128309140786

2. Shapiro FD, Eyre DR. Collagen polymorphism in extracellular matrix of human osteosarcoma. *J Natl Cancer Inst*. 1982;69(5):1009‐1016.

3. Cui J, Dean D, Hornicek FJ, et al. The role of extracellular matrix in osteosarcoma progression and metastasis. *J Exp Clin Cancer Res* 2020;39:178. https://doi.org/10.1186/s13046-020-01685-w

4. Nadiarnykh O, LaComb RB, Brewer MA, et al. Alterations of the extracellular matrix in ovarian cancer studied by Second Harmonic Generation imaging microscopy. *BMC Cancer*. 2010;10:94. https://doi.org/10.1186/1471-2407-10-94

5. Provenzano PP, Eliceiri, K.W., Campbell, J.M. et al. Collagen reorganization at the tumor‐stromal interface facilitates local invasion. *BMC Med*. 2006;4:38. https://doi.org/10.1186/1741-7015-4-38

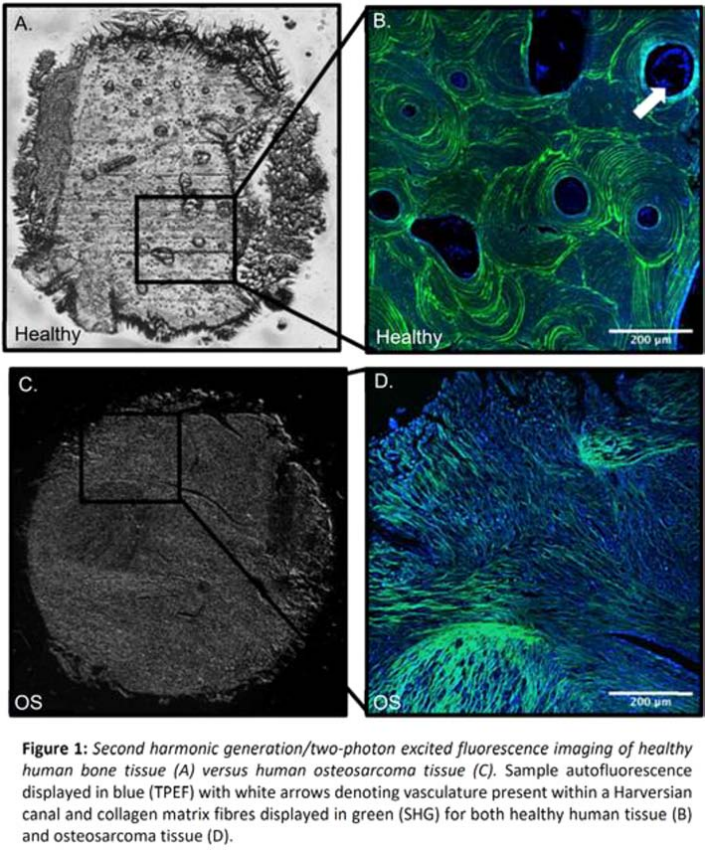

## Other

## A signal processing and machine learning methodology for classification of activity types in postmenopausal women using triaxial accelerometer data

### Cameron Huggins ^1^, Rebecca Clarke^1^, Daniel Abásolo^1^, Erreka Gil‐Rey^2^ , Jonathan Tobias^3^, Kevin Deere^3^, Sarah Allison^4^

#### ^*1*^ *Faculty* *of Engineering and Physical Sciences, University of Surrey, Guildford, UK.* ^*2*^ *Faculty* *of Psychology and Education, University of Deusto,* *San‐Sebastián* *, Spain.* ^*3*^ *Bristol* *Medical School (* *THS* *), University of Bristol, Bristol, UK.* ^*4*^ *Faculty* *of Health and Medical Sciences, University of Surrey, Guildford, UK*

**Abstract**

**Introduction:** Triaxial accelerometers are widely used to assess physical activity; however, this is generally in terms of energy consumption; methods for classifying the output in terms of different types of activity, of relevance to effects of exercise on the skeleton, are not currently available.

**Purpose:** The purpose of this study was to assess the accuracy of four machine learning models (k‐Nearest Neighbors (NN) Manhattan, k‐NN Euclidian, Decision Tree (DT), and Support Vector Machine (SVM)) on a binary movement classification (stationary and walking) and a tertiary movement classification (stationary, walking, and running) from postmenopausal women.

**Methods:** Eighty postmenopausal women performed a submaximal incremental shuttle test on an indoor track, of which 30 also performed the same test on an indoor treadmill, all while wearing a hip‐worn triaxial accelerometer. The raw accelerometer data were preprocessed by filtering out noise and segmenting the data into 2‐second segments per subject. A feature set was then generated that consisted of four different wavelet decompositions across three levels using the Daubechies 2 mother wavelet. To evaluate the method using leave‐one‐out validation, each feature set per subject was used sequentially as the test set for the four machine learning models, with the remainder of the subjects making up the training set.

**Results:** The highest mean leave‐one‐out classification accuracy for the binary classifier, 99.57% ± 1.383%, was produced by the k‐NN Manhattan classifier. The highest mean leave‐one‐out classification accuracy for the tertiary classifier, 92.64% ± 5.385%, was also produced by the k‐NN Manhattan classifier. Sixty (60) of 110 tests for the binary k‐NN Manhattan classifier produced results of 100% with two tests that produced results significantly lower than the mean (>3 standard deviations [SDs]). The tertiary k‐NN Manhattan classifier's highest individual test accuracy was 99.78% and had three tests that produced results significantly lower than the mean (>3 SD).

**Conclusions:** The k‐NN Manhattan classifier trained with triaxial accelerometer data provided accurate recognition of specific types of activity in postmenopausal women.

## The mediating role of lean soft tissue in the relationship between somatic maturation and bone density in adolescent practitioners and non‐practitioners of sports

### Ricardo R Agostinete ^1^, André O Werneck^2^, Santiago Maillane‐Vanegas^1^ , Luis Gracia‐Marco^3^ ^,4^, Esther Ubago‐Guisado^3^ ^,4,5^, Annie M Constable^6,7^, Romulo A Fernandes^1^, Dimitris Vlachopoulos^6^

#### ^*1*^ *Laboratory* *of* *Investigation* *in Exercise (* *LIVE* *), Department of Physical Education, Sao Paulo State University (* *UNESP* *), Presidente Prudente, Brazil.* ^*2*^ *Center* *of Epidemiological Research in Nutrition and Health, Department of Nutrition, School of Public Health, University of São Paulo, São Paulo, Brazil.* ^*3*^ *PROFITH* *“* *PROmoting FITness* *and Health through Physical Activity”, Research Group, Sport and Health University Research Institute (* *iMUDS* *), Department of Physical Education and Sport, Faculty of Sport Sciences, University of Granada, Granada, Spain.* ^*4*^ *Instituto* *de Investigación Biosanitaria ibs* *GRANADA* *, Granada, Spain.* ^*5*^ *Escuela* *Andaluza de Salud Pública (* *EASP* *), Granada, Spain.* ^*6*^ *Children* *'s Health and Exercise Research Centre, Sport and Health Sciences, University of Exeter, Exeter, UK.* ^*7*^ *Institute* *of Biomedicine, University of Eastern Finland, Kuopio Campus, Kuopio, Finland*

**Abstract**

**Purpose:** The studies in the literature only analyzed the association between biological maturation and bone variables directly. The potential mediation role of lean soft tissue (LST) in this association has not been previously investigated. Thus, this study aimed to identify the mediating effect of LST in the association between somatic maturation and areal bone mineral density (aBMD) in adolescents by sex and sport participation.

**Methods:** The sample included 558 adolescents (401 males, mean age of 14.0 years) that were practitioners of sports (11 sport modalities, *n* = 402) and a non‐sport group (*n* = 157). Somatic maturation was assessed by using a validated peak height velocity prediction equation. Dual‐energy X‐ray absorptiometry (DXA) was used to assess aBMD (upper and lower limbs, spine and total body less head [TBLH]) and LST. The mediation analyses were performed using the command “med4way” on Stata 15.1, with statistical significance level at *p* < 0.05. The theoretical mediation model is presented in Figure 1.

**Results:** For both sexes, LST mediated the association between somatic maturation and aBMD at all skeletal sites (mediation percentage ranging from 36.3% to 75.4%). For sport and non‐sport groups, the LST also mediated the association between somatic maturation and aBMD at all skeletal sites (mediation percentage ranging from 51.6% to 85.6%). The direct effect was observed in all groups, except for lower limbs and TBLH in the non‐sport group.

**Conclusion:** The association between somatic maturation and aBMD was mediated by LST in adolescents of both sexes and regardless of involvement in organized sports. Our findings highlighted the role of improving LST to mitigate the association of somatic maturation with aBMD.

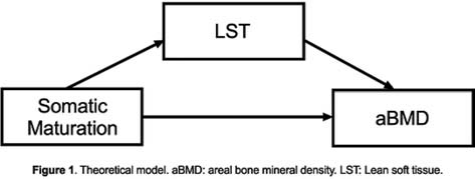

## Rare Conditions

## Pycnodysostosis: Report of two cases

### Kawtar Nassar, Saadia Janani

#### *Department of Rheumatology, Ibn Rochd University Hospital Center, Hassan* *II* *University, Faculty of Medicine and Pharmacy. Casablanca, Casablanca, Morocco*

**Abstract**

**Background:** Pycnodysostosis, or Toulouse‐Lautrec syndrome, is a rare autosomal recessive skeletal dysplasia, due to a defect in the gene encoding cathepsin K. Its prevalence is 1/100,000. It is characterized by short‐limbed, short stature, typical facial appearance; convex nasal ridge and small jaw with obtuse mandibular angle, osteosclerosis with increased bone fragility, acro‐osteolysis of the distal phalanges, delayed closure of the cranial sutures, and dysplasia of the clavicle. The diagnosis is usually based on the clinic and bone X‐rays, or better by the study of the gene abnormally located on the cathepsin K gene (1q21). We present two isolated and separate cases, with typical clinical‐radiological criteria, who were admitted for the assessment of bone status.

**Method:** Two Case reports.

**Results:** We present two isolated and separate cases of pycnodysostosis with the context of consanguinity and past history of fractures. The cases are a 32‐year‐old woman and a 19‐year‐old man. The clinical exam found short stature, brachydactyly, dystrophic nails, facial dysmorphia with frontal bossing, bilateral ocular proptosis, micrognathia, open fontanels, prominent nose, and abnormal teeth. Radiography showed acro‐osteolysis of the distal phalanges, diffuse osteosclerosis, brachydactyly, and wide cranial sutures (Figures). Both patients presented normal levels of vitamin D, phosphatase alkaline, thyroid‐stimulating hormone (TSH), free T4, blood, and urinary calcium and phosphate levels. They were followed at bone disease unit in rheumatology department without any complications.

**Conclusion and Discussion:** Pycnodysostosis is a rare inherited disorder of the bone, autosomal recessive. The diagnosis is essentially based on radioclinical criteria. The bone pathological fractures occur due to sclerosis. Skull bones appear thickened with generalized osteosclerosis. It can be revealed by fractures or exceptionally neurological complications that require specific treatment. The main differential diagnosis is osteopetrosis, of which there is a close and complex link. The treatment is based on that of complications. The prognosis is generally not involved.

## Investigating the molecular basis of Cole Carpenter syndrome

### Charlotte Clews ^1^, Adam Benham^1^, Meena Balasubramanian^2^

#### ^*1*^ *Durham* *University, Durham, UK.* ^*2*^ *Sheffield* *Children*'*s Hospital, Sheffield, UK*

**Abstract**

Cole Carpenter syndrome (CCS) is a rare genetic syndrome affecting bone integrity and durability. It is believed to be related to osteogenesis imperfecta (OI), a group of genetic disorders commonly known as brittle bone disease. The molecular mechanism underpinning the syndrome has yet to be discovered; however, two distinct types have been identified: CCS1 and CCS2, linked to mutations in protein disulfide isomerase (PDI), and SEC24D (a component of the coat protein II [COPII] vesicle export complex), respectively. CCS1 is characterized by a single amino acid substitution mutation (Y393C) in PDI, an endoplasmic reticulum (ER)‐specific PDI and molecular chaperone involved in collagen folding. Without this chaperone, procollagen chains cannot fold into the triple helical tertiary structure of Collagen I, and this misfolded protein may accumulate in the ER, activating the unfolded protein response (UPR). It has been suggested that a failure to resolve this response may result in the brittle bone phenotype of CCS1. In this project, we have used microscopy and biochemical techniques in both primary cells and established cell lines to investigate the behavior of Y393C‐PDI and study the downstream effects of this mutation in the cells. Investigating collagen production, ER and lysosome organelle morphologies alongside overall cell viability provides preliminary work to shed light on the possible cause of this predominantly bone‐specific phenotype. This work will help to further understand the main cause of CCS1, alongside potentially other OI‐related disorders.

## Burosumab treatment in a child with cutaneous skeletal hypophosphatemia syndrome: a case report

### Manal Mustafa ^1^, Zulf Mughal^2^

#### ^*1*^ *Latifa* *Women and children hospital, Dubai,* *UAE* *.* ^*2*^ *Royal* *Manchester Children's Hospital, Manchester, UK*

**Abstract**

**Introduction:** The cutaneous skeletal hypophosphatemia syndrome (CSHS) is a rare mosaic disorder caused by somatic gain‐of‐function *RAS* mutations. It is characterized by segmental epidermal nevi and fibroblast growth factor‐23 (FGF23)‐mediated hypophosphatemic rickets. These patients also have dysplastic cortical skeletal lesions.

**Case Presentation:** We describe a 4‐year‐old Emirati child with CSHS, whose hypophosphatemic rickets and dysplastic skeletal lesions failed to heal after 1 year of treatment with oral phosphate supplements and alfacalcidol (conventional treatment). The diagnosis of CSHN with FGF23‐mediated hypophosphatemic rickets was made in our patient due to increased urinary phosphate excretion and hypophosphatemia, in the face of normal serum parathyroid hormone (PTH) levels and inappropriately elevated plasma FGF23 levels (Table 1). The whole‐exome sequence on cells from nevoid skin biopsy revealed a somatic missense variant c.182A>G p.(Gin61Arg) (chr11:533874;hg19) in the HRAS gene (OMIM *190020; chromosome 11p15.5).

**Results:** Recently, burosumab, a fully human immunoglobulin G1 monoclonal antibody to FGF23 was approved for the treatment of children with X‐linked hypophosphatemia (XLH), a multisystem disorder caused by increased expression of FGF23. Treatment of our patient with burosumab, for 12 months, resulted in normalization of her serum inorganic phosphate and alkaline phosphatase levels (Graph 1), healing of rickets, and improvement in her symptoms of myopathy and quality of life.

**Conclusion:** To the best of our knowledge, this is the first case report to describe the usage of the new treatment modality (burosumab) in a child with CSHS. Burosumab may have a role in the treatment of CSHS and dysplastic skeletal lesions.

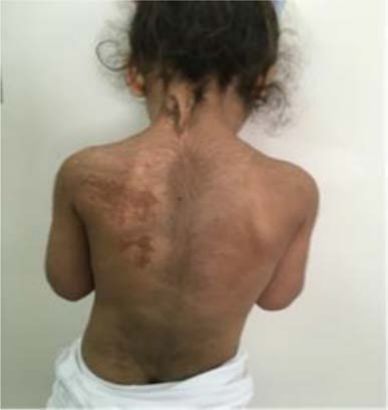

**Table:** 

Parameter	Biochemical markers after 7 days washout period from conventional Rx
Fasting serum phosphate	2.6 mg/dL (3.4–5.5)
Alkaline phosphatase	
PTH intact	12.5 pg/mL (15–65)
Serum calcium	9.5 mg/dL (8.4–10.6)
25‐hydroxy vitamin D	35.3 ng/mL (20–60)
1,25‐dihydroxyvitamin D (1,25 (OH)_2_D)	30.4 pg/mL (30–90)
Urine TmP/GFR	2.1 mg/dL (4–8)
C‐terminal fibroblast growth factor‐23 (FGF23)	479 RU/mL (39–91)

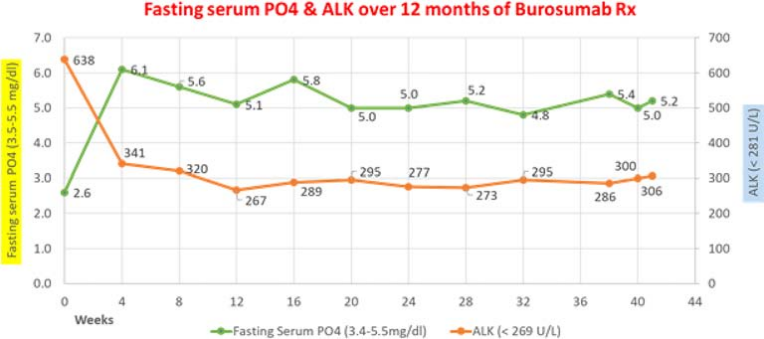

## Longitudinal and flare‐up–specific biomarkers in fibrodysplasia ossificans progressiva (FOP): data from a Global Natural History Study

### Robert J. Pignolo^1^, Mona Al Mukaddam^2^, Geneviève Baujat^3^, Carmen De Cunto^4^, Edward C Hsiao^5^, Richard Keen ^6^, Kathleen Harnett^7^, Rose Marino^7^, Mario Ippolito^8^, Euvian Tan^8^, Manjinder Bains^8^, Frederick S Kaplan^2^

#### ^*1*^ *Department* *of Medicine, Mayo Clinic, Rochester,* *MN* *,* *USA* *.* ^*2*^ *Departments* *of Orthopaedic Surgery & Medicine, The Center for Research in* *FOP* *and Related Disorders, Perelman School of Medicine, University of Pennsylvania, Philadelphia,* *PA* *,* *USA* *.* ^*3*^ *Département* *de Génétique, Institut* *IMAGINE* *and Hôpital Universitaire* *Necker‐Enfants* *Malades, Paris, France.* ^*4*^ *Pediatric* *Rheumatology Section, Department of Pediatrics, Hospital Italiano de Buenos Aires, Buenos Aires, Argentina.* ^*5*^ *Division* *of Endocrinology and Metabolism,* *UCSF* *Metabolic Bone Clinic, Institute of Human Genetics, and* *UCSF* *Program in Craniofacial Biology, Department of Medicine, University of* *California‐San* *Francisco, San Francisco,* *CA* *,* *USA* *.* ^*6*^ *Centre* *for Metabolic Bone Disease, Royal National Orthopaedic Hospital, Stanmore, UK.* ^*7*^ *Ipsen* *, Newton,* *MA* *,* *USA* *.* ^*8*^ *Ipsen* *, Slough, UK*

**Abstract**

**Background:** Fibrodysplasia ossificans progressiva (FOP) is an ultra‐rare, severely debilitating genetic disorder characterized by episodic heterotopic ossification (HO) and flare‐ups. Identification of biomarkers would be useful to predict flare‐ups, monitor FOP disease progression, and to identify when interventions are warranted and assess responses to these interventions.

**Objective:** To evaluate putative serum and urine biomarkers at baseline (BL), annually, and during the course of flare‐ups in a longitudinal FOP natural history study (NHS).

**Methods:** Individuals with FOP aged ≤65 years with a documented ACVR1R206H mutation were eligible to participate in a prospective, 36‐month NHS (NCT02322255) study investigating the longitudinal progression of FOP. This analysis evaluated biomarker data at BL and month 12, and during flare‐ups. Blood and urine samples were collected at BL and annual study visits, and at days 1, 42, and 84 of imaged flare‐ups to measure biomarkers of inflammation, angiogenesis, and bone/cartilage turnover. Low‐dose whole‐body computed tomography (WBCT, excluding the head) was performed to assess HO at months 12, 24, and 36; flare‐up HO volumes were assessed by CT at the flare‐up sites. Magnetic resonance imaging (MRI) or ultrasound was used to assess flare‐ups for the presence of edema. Data were analyzed using descriptive statistics.

**Results:** BL and month 12 assessments were completed for 99 of 114 participants; 93 had evaluable WBCT HO at both time points, of whom 37 (39.8%) had new HO at month 12. Overall, biomarker values did not change substantially from BL to month 12, and mean values were similar between participants with versus those without new HO at month 12 (Table). A total of 52 flare‐ups were CT‐imaged, of which 14 (26.9%) resulted in new HO at Day 84. Out of 42 flare‐ups assessed at Day 1 by MRI or ultrasound, 29 (69.0%) had edema; of these, 10 (34.5%) had new HO at Day 84 (versus 19 [65.5%] with no new HO). No serum biomarker was identified that was predictive of flare‐ups with BL edema or that resulted in development of new HO.

**Conclusions:** The identification of biomarkers to predict disease progression could greatly benefit the clinical management of FOP. As results from this NHS did not identify a signal for a longitudinal or flare‐up‐specific biomarker for FOP, potentially due to the limited panel of biomarkers tested, a narrow sampling window, and/or missing data for some participants, further research is required.

**Table:** **Table**. Mean change from BL in biomarker values at month 12 in participants with and without new HO

Biomarker	Mean change from baseline (SEM)	
	Without new HO	With new HO
Erythrocyte sedimentation rate (mm/h)	3.3 (1.7); *n* = 32	−3.9 (1.9); *n* = 21
C‐reactive protein (mg/L)	−0.4 (1.9); *n* = 45	−1.0 (1.0); *n* = 31
Interleukin‐6 (ng/L)	−1.0 (0.4); *n* = 44	−0.3 (0.4); *n* = 26
Interleukin‐1 beta (ng/L)	0.0 (0.0); *n* = 44	0.1 (0.1); *n* = 26
Tumor necrosis factor alpha (ng/L)	−0.3 (0.1); *n* = 44	−0.1 (0.1); *n* = 26
Creatine phosphokinase (U/L)	−5.8 (5.9); *n* = 45	3.1 (7.1); *n* = 31
Lactate dehydrogenase (U/L)	−18.7 (4.5); *n* = 45	−20.5 (7.8); *n* = 31
Fibroblast growth factor/urine creatine (μg/kg)	0.8 (0.7); *n* = 32	−1.1 (0.6); *n* = 21
Vesicular endothelial growth factor (ng/L)	−22.6 (12.1); *n* = 44	−16.4 (27.2); *n* = 25
Osteocalcin (μg/L)	−6.9 (3.7); *n* = 44	−12.0 (5.4); *n* = 29
Bone‐specific alkaline phosphatase (μg/L)	−4.1 (2.0); *n* = 44	3.9 (4.3); *n* = 29
C‐terminal propeptide of type 1 procollagen (μg/L)	−22.0 (13.6); *n* = 44	0.6 (23.3); *n* = 27
N‐terminal propeptide of type 1 procollagen (μg/L)	−66.6 (32.4); *n* = 44	−110.6 (46.9); *n* = 28
Collagen‐derived retinoic acid protein (ng/L)	−120.0 (56.5); *n* = 45	−87.3 (95.2); *n* = 26
C‐terminal telopeptide (μg/L)	−0.1 (0.0); *n* = 42	−0.1 (0.1); *n* = 27

Data not available for all participants at all time points. Biomarkers measured in serum, except fibroblast growth factor/urine creatine (urine) and erythrocyte sedimentation rate (blood). BL = baseline; HO = heterotopic ossification; SEM = standard error of the mean.

## Mobility and health‐related quality of life in adults with pediatric‐onset hypophosphatasia treated with asfotase alfa: interim analysis from the UK Managed Access Agreement Study

### Katie E. Moss ^1^, Richard Keen^2^, Katherine A Kirkwood^3^, Alexandros Zygouras^4^, Jennifer S Walsh^5^, Judith S Bubbear^2^

#### ^*1*^ *St* *. George′s University Hospitals, London, UK.* ^*2*^ *Royal* *National Orthopaedic Hospital, Stanmore, UK.* ^*3*^ *PAREXEL* *International Corp., Newton,* *USA* *.* ^*4*^ *Alexion* *Pharmaceuticals, Inc., Uxbridge, UK.* ^*5*^ *Northern* *General Hospital, Sheffield, UK*

**Abstract**

**Introduction:** Hypophosphatasia (HPP) is a rare, inherited metabolic disease caused by inadequate tissue‐nonspecific alkaline phosphatase (TNSALP) activity. Asfotase alfa (AA) is a TNSALP enzyme replacement therapy approved for the treatment of pediatric‐onset HPP. Data on the effect of AA on function and health‐related quality of life (HRQoL) in adults with HPP are limited.

**Methods:** This prospective study used data from the UK Managed Access Agreement (MAA) to assess mobility and HRQoL outcomes in nine adults with HPP treated with AA for ≥6 months. Data were collected at the time of MAA enrollment, 3 and 6 months after enrollment, and every 6 months thereafter. Patients were formally assessed every 12 months for AA discontinuation if they experienced a 6‐minute walk test (6MWT) improvement of <25 m or <10% over baseline, a decrease in Bleck score of more than one level, continued fractures over a 3‐year period, or no reduction in pain (no reduction in analgesic use, <2‐point improvement in Brief Pain Inventory–Short Form [BPI‐SF] score; <0.15 improvement in EuroQoL 5‐Dimension [EQ‐5D] 5‐level score). We report interim data from adults treated for HPP for up to 2 years. Results are presented as median (minimum, maximum).

**Results:** Of 12 adults enrolled, nine were included in the analysis population (six women, three men; all treatment‐naïve) and 11 were evaluated for safety. Age at enrollment was 47.0 (23.0, 60.0) years. Bleck score improved from 4.0 (2.0, 6.0) at baseline to 6.5 (5.0, 7.0) at 18 months (Figure); no patient's Bleck score decreased from baseline. 6MWT distance walked increased from 165.0 (75.0, 360.0) m at baseline to 237.5 (100.0, 543.0) m at 18 months, with total change greater than the minimal clinically important difference (MCID) of 31 m. BPI‐SF scores improved from 8.0 (7.0, 9.9) at baseline to 5.2 (1.0, 7.0) at 18 months. Improvements in EQ‐5D 3‐Level utility scores were greater than the MCID of 0.074. For patients with over 7 days of AA treatment, no new fractures occurred over a 2‐year period. Injection site reactions were reported in 72.7% of patients. Serious adverse events (AEs) were reported in two patients (flank pain, musculoskeletal/connective tissue disorder, and unknown); these were not related or unlikely related to treatment. No patient discontinued because of an AE or met prespecified discontinuation criteria.

**Conclusion:** The data suggest that AA improves mobility, pain, and HRQoL in adults with pediatric‐onset HPP, with a favorable benefit/risk profile.

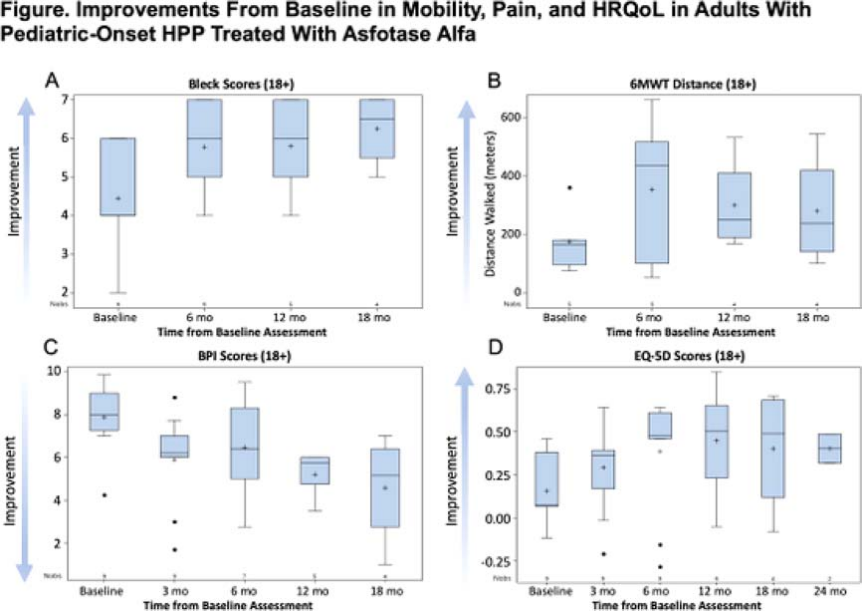

## Children with NF1 have significant deficits in muscle function

### Amish Chinoy ^1,2^, Alex Ireland^3^, Grace Vassallo^1^, Steve Roberts^2^, Judith Eelloo^1^, Eileen Hupton^1^, Peter Clayton^2,1^, Raja Padidela^1,2^, Zulf Mughal^1,2^

#### ^*1*^ *Manchester* *University Hospitals* *NHS* *Foundation Trust, Manchester, UK.* ^*2*^ *University* *of Manchester, Manchester, UK.* ^*3*^ *Manchester* *Metropolitan University, Manchester, UK*

**Abstract**

**Introduction:** A phenotype of muscle weakness has been identified in children with neurofibromatosis type 1 (NF1). However, there are a number of clinically‐relevant components of muscle function which have not been characterized in this population. We describe the muscle function of children with NF1 using a range of assessment tools, and have studied the relationship with other features of NF1 such as small muscle size, poor balance, perceived fatigue, and poor quality of life.

**Methods:** Children had their dynamic muscle function measured using Leonardo Mechanography (Novotec Medical), which measures peak power and force generation on single two‐legged jumping and multiple one‐legged hopping, respectively, and also jumping efficiency and balance. Grip force (isometric muscle function) and 6‐minute walk test (6MWT, assessing functional capacity) were also measured. Magnetic resonance imaging of the right thigh was undertaken to measure cross‐sectional area of knee extensor muscles. Questionnaires of perceived fatigue (PedsQL Multi‐dimensional Fatigue Scale) and quality of life (Child Outcome Rating Scale) were also completed. Data was compared to age‐ and gender‐specific reference data where available, to calculate standard deviation scores (SDS).

**Results:** A total of 44 children were recruited (20 males, 24 females; age range 6.1–16.5 years, mean age 10.6 years). Children with NF1 had significantly reduced jumping power SDS (mean −2.1; 95% confidence interval [CI], −2.5 to −1.8), jumping efficiency SDS (mean −1.2; 95% CI, −1.5 to −0.9), hopping force SDS (mean −2.4; 95% CI, −2.8 to −1.9), grip force SDS (mean −2.4; 95% CI, −2.7 to −2.1), and 6MWT SDS (mean −2.4; 95% CI, −2.8 to −2.0) (*p* < 0.001 for all), regardless of age or gender. Muscle size strongly correlated with jumping power (*r* = 0.80, *p* < 0.001), jumping efficiency (*r* = 0.45, *p* = 0.003), hopping force (*r* = 0.54, *p* < 0.001), and grip force (*r* = 0.53, *p* < 0.001), even when controlling for age, gender, height, and weight. No correlations were evident with measures of muscle function for balance performance, fatigue scores, or quality of life scores.

**Discussion/Conclusion:** Children with NF1 have very significant impairments in a variety of muscle function tests, indicating deficits in strength, endurance, and dynamic muscle function. These deficits exist throughout childhood, and regardless of gender. These deficits in muscle function correlate with muscle size, suggesting this impairment is at least partly due to issues with muscle bulk, highlighting the potential for improvement with physiotherapy exercises aimed at improving muscle bulk. These deficits do not seem to strongly impact on other features of NF1, presumably because these features are multifactorial in etiology.

## Pachydermoperiostosis—a rare form of hypertrophic osteoarthropathy mimicking acromegaly

### Swayamsidha Mangaraj, Liza Mohapatra

#### *IMS* *and* *SUM* *Medical College and Hospital, Bhubaneswar, India*

**Abstract**

**Introduction:** Pachydermoperiostosis (PDP) is a rare disorder characterized by digital clubbing, thickening of the skin (pachyderma), hyperhidrosis, and new periosteal bone formation. PDP can sometimes clinically mimic acromegaly due to many similar findings in both of these disorders. We describe an interesting case of a young male who was referred for evaluation of acromegaly but was subsequently diagnosed to have a complete form of PDP.

**Case Description:** A 22‐year‐old male presented with complaints of enlargement of distal ends of hand and feet, coarsening of facial features and increased seborrhea for around 8 years. There was no history of chronic fever, headache, or cardiac, pulmonary, hematologic, or gastro intestinal disease in past. There was no history of similar complaints in any family members. Clinical examination revealed presence of bilateral grade IV digital clubbing in upper and lower limbs, prominent facial furrows, hyperhidrosis, and bilateral blepharoptosis. The rest of the systemic examination was clinically unremarkable. Complete blood count, hepatic function tests, renal function tests, serum electrolytes, and glycemic parameters were within normal limits. Chest X‐ray and 2D echocardiogram were normal. Hormonal analysis including thyroid function tests and insulin‐like growth factor 1 were normal. Two‐hour post‐glucose growth hormone values were suppressed. X‐ray of both hands and feet showed cortical thickening, periosteal reaction, and bulbous enlargement of finger tips. Based on the above findings a diagnosis of pachydermoperiostosis was made. The patient was counseled regarding the nature of the disease and advised regarding use of nonsteroidal anti‐inflammatory drugs (NSAIDs) on a short‐term as and when required basis for symptomatic management.

**Discussion:** PDP is a rare inherited form of primary hypertrophic osteoarthropathy. PDP can be diagnosed based on established major and minor criteria. The three major criteria include pachyderma, periostosis and digital clubbing. Similarly, the minor criteria include hyperhidrosis, arthralgia, gastric ulcer, cutis verticis gyrata, blepharoptosis, joint effusion, edema, seborrhea, acne, and flushing. The disease has a male preponderance. The three major forms of PDP include: complete form (involving all three major symptoms); incomplete form (presence of periostosis but without pachyderma), and forme fruste (presence of pachyderma with minimal or no skeletal anomalies). Symptomatic management with NSAIDs, analgesics, and bisphosphonates have been tried. Apart from acromegaly, other clinical mimics include psoriatic arthritis, rheumatoid arthritis, and even thyroid acropachy.

**Conclusion:** PDP is a rare disorder that can clinically simulate acromegaly. Knowledge about this entity among physicians would be helpful for early identification and appropriate management.

## Physical function and physical activity in adults with X‐linked hypophosphatemia

### Giorgio Orlando ^1^, Judith Bubbear^2^, Shane Clarke^3^, Richard Keen^2^, Matthew Roy^3^, Marian Schini^4^, Jennie Walsh^4^, Kassim Javaid^5^

#### ^*1*^ *Manchester* *Metropolitan University, Manchester, UK.* ^*2*^ *Royal* *National Orthopaedic Hospital, London, UK.* ^*3*^ *Bristol* *Royal Infirmary, Bristol, UK.* ^*4*^ *University* *of Sheffield, Sheffield, UK.* ^*5*^ *University* *of Oxford, Oxford, UK*

**Abstract**

Although muscle weakness is commonly reported in adults with X‐linked hypophosphatemia (XLH), there is a dearth of information on multiple components of physical function in this population. We examined upper body strength, lower body power, functional capacity, mobility, and physical activity (PA) level and explored the relationships among these variables in adults with XLH.

Participants were recruited as part of a UK‐based prospective cohort study, the RUDY Study. They underwent a clinical visit and physical examination, including assessment of handgrip strength, jump power (mechanography), 6‐minute walk test (6MWT), short physical performance battery (SPPB), and the International Physical Activity Questionnaire (IPAQ). Performance data were analyzed using parametric and nonparametric tests, whereas correlations between physical activity and performance data were assessed by univariate analysis.

Twenty‐nine adults with XLH (52% males) with a mean age of 45.5 ± 15.9 years were recruited. Jump power and 6MWT distances were 47% and 39% lower, respectively, in individuals with XLH compared with normative values (*p* < 0.0001), whereas handgrip strength values were similar to expected values (*p* = 0.51). Twelve of 29 participants (41%) had an SPBB score of ≤9, indicating impaired mobility. Low and moderate PA levels were noted in 72% and 28% of the participants, and none were classified as highly active. These deficits were similar in individuals of both sexes and were not associated with age. Univariate analysis revealed only a correlation between PA and exercise‐induced feeling inventory (EFI) (*r* = 0.505, *p* = 0.023).

Adults with XLH are characterized by marked deficits in lower limb muscle power and functional capacity, with a high incidence of impaired mobility and inactivity. In addition to metabolic effects of XLH, low physical activity may contribute to deficits in lower limb power. Further studies are required to understand underlying mechanisms and to develop novel treatment approaches to improve physical function and mobility.

## Two rare GALNT3 mutations associated with high bone mass without evidence of disturbed phosphate homeostasis

### Neelam Hassan ^1^, Marc van der Kamp^1^, Celia Gregson^1^, Emma Duncan^2^, Jon Tobias^1^

#### ^*1*^ *University* *of Bristol, Bristol, UK.* ^*2*^ *King* *'s College London, London, UK*

**Abstract**

**Introduction:** This study aimed to identify novel monogenic causes of unexplained high bone mass (HBM), defined as a total hip and/or first lumbar vertebral bone mineral density (BMD) *Z*‐score of ≥+3.2.

**Methods:** All participants in the UK HBM cohort (355 HBM cases, 200 unaffected relatives) underwent clinical assessment and DXA scanning. Whole‐exome sequencing (WES) was undertaken in likely informative pedigrees, with data analyzed for carriage of at least one novel or rare (minor allele frequency [MAF] <0.005) nonsynonymous single‐nucleotide variant (SNV) or indel in a highly conserved region of a gene, segregating with HBM within the pedigree. Data were then filtered based on functional prediction using Polyphen‐2 and Sorting Intolerant From Tolerant (SIFT). WES data from the UK HBM cohort and 126 HBM cases in the Anglo‐Australasian Osteoporosis Genetics Consortium (AOGC) were then interrogated to identify other individuals carrying either the same or another rare variant within the same gene. WES data from 493 low bone mass (LBM) cases in AOGC were analyzed to ensure absence of the variant. Variants were validated using Sanger sequencing. Protein homology modeling was performed using the SwissModel server with visualization in PyMOL.

**Results:** Exome sequencing in a pedigree with unexplained and apparently autosomal dominant HBM (Fig. 1) identified a rare (MAF 0.000016) heterozygous missense variant in *GALNT3* (NM_004482.4:c.1657C>T, p.Arg553Trp) segregating with HBM. Analysis of a further 492 cases from UK HBM and AOGC resulted in the identification of another individual with a different, rare (MAF 0.000025), heterozygous missense variant in *GALNT3* (NM_004482.4:c.831T>A, p.Asp277Glu). Clinical and biochemical characteristics are described in Table 1.

*GALNT3* encodes a Golgi‐associated glycosyltransferase that initiates mucin‐type *O*‐glycosylation. p.Arg553Trp will result in disruption of salt‐bridge interactions, with predicted instability of the GALNT3 protein. p.Asp277Glu is predicted to disrupt manganese binding and consequently the catalytic function of GALNT3.

Common variants in *GALNT3* are associated with BMD in GWAS. Homozygous loss‐of‐function mutations in *GALNT3* are associated with disorders of phosphate homeostasis, specifically familial tumoral calcinosis (FTC) and hyperostosis‐hyperphosphatemia syndrome (HHS). However, our cases had no clinical or biochemical features of either condition. There was no evidence of disturbed phosphate homeostasis and BMD has previously been reported as low or normal in FTC and HHS when it has been measured.

**Conclusion:** Rare variants in *GALNT3* may cause HBM, without disturbing phosphate homeostasis. Further studies are underway to elucidate the mechanism by which this occurs.

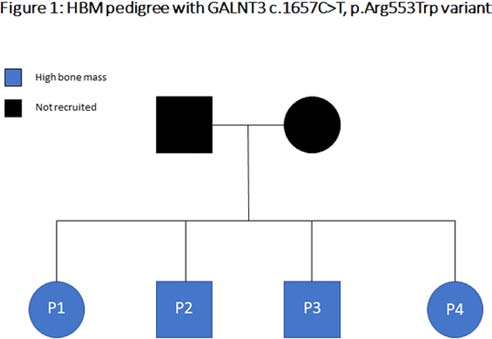

**Table:** 

	HBM pedigree *GALNT3* c.1657C>T, p.Arg553Trp			Additional isolated HBM case *GALNT3* c.831T>A, p.Asp277Glu	
Parameter	P1	P2	P3	P4	S1
Age (years)	64	58	67	59	61
Sex	Female	Male	Male	Female	Female
Ethnicity	British (white)	British (white)	British (white)	British (white)	British (white)
Height (cm)	162	182	178	160	162
Weight (kg)	89	94	86	74	69
BMI (kg/m^2^)	33.9	28.4	27.1	28.9	26.3
DXA L1 *Z*‐score	+5.2	+2.9	+3.4	+3.3	+5.2
DXA TH *Z*‐score	+5.1	+2.0	+1.8	+2.4	+3.2
Adult fracture	No	No	Yes	No	No
Dental overcrowding	Yes	Yes	Yes	Yes	No
Torus	No	No	Yes	No	No
Nerve compression	No	No	No	No	No
Buoyant when swimming	Yes	Yes	Not known	Yes	No
Bloods^a^					
Albumin (g/L)	44	45	40	47	51
Adjusted calcium (mmol/L)	2.43	2.28	‐	‐	‐
Phosphate (mmol/L)	1.2	0.92	‐	‐	1.29
iFGF23 (pg/mL)	52.5	22.8	43.4	‐	‐
cFGF23 (RU/mL)	59.5	28	25.2	‐	‐
ALP (IU/L)	78	70	50	‐	65
P1NP (μg/L)	27	16	28	‐	15
CTX (μg/L)	0.09	0.09	0.05	‐	0.09
Osteocalcin (μg/L)	12	12	12.21	‐	6

## Poster Abstracts

### Animal Models

## Effect of the microenvironment on osteoclast driven bone remodeling

### Erika Abbondati ^1^, Colin Farquharson^1^, Gurå Bergkvist^2,1^

#### ^*1*^ *The* *Roslin Institute, Easter Bush campus, Roslin, UK.* ^*2*^ *Royal* *Dick School of Veterinary Studies, Easter Bush campus, Roslin, UK*

**Abstract**

**Background:** Despite their rigid appearance, bones are dynamic organs going through continuous remodeling cycles. Osteoclasts are responsible for bone resorption and their activation is regulated by hormones, cytokines, growth factors, and physical properties of the bone microenvironment. Changes to this microenvironment can have profound effects on both osteoclastogenesis and osteoclast resorption activity. The aim of this project was to gain a better understanding of how an altered microenvironment influences osteoclast biology using companion animal cancers as a model of disrupted microenvironment. Feline oral squamous cell carcinoma (FOSCC) and canine osteosarcoma were selected because they represent good models of their human counterparts and they have the ability to invade bone and alter osteoclast activity in vivo.

**Method:** A novel FOSCC cell line was isolated from a bone invasive tumor and characterized. This bone invasive cell line, and a previously characterized, non‐bone invasive FOSCC cell line were used. Conditioned media from the cell lines was added during osteoclast differentiation from feline bone marrow precursors in presence of colony stimulating factor 1 (CSF‐1) and receptor activator of nuclear factor κB ligand (RANKL). Similarly, the effect of four different canine osteosarcoma cell lines conditioned media was tested on osteoclast differentiation from canine bone marrow cells. To determine which factors (known and unknown) might have an effect on osteoclast differentiation and activity, conditioned media from all the cell lines tested was analyzed with mass spectrophotometry.

**Results:** FOSCC conditioned media was able to enhance osteoclast formation and resorption activity on mineralized substrates. Moreover, when cultured in hypoxic conditions (0.5% O_2_) the presence of FOSCC conditioned media positively influenced survival of feline osteoclast precursor cells. Of the four canine osteosarcoma cell lines tested, only one showed an enhancing effect on osteoclastogenesis on plastic. A list of proteins emerged from mass spectrophotometry analysis of the conditioned media. These were searched against the literature to identify possible candidates able to mediate the effects observed experimentally.

**Conclusions:** The FOSCC cell lines enhancing effect observed on osteoclast differentiation and activity is likely to be mediated by factors secreted by the cell lines in their microenvironment (the conditioned media). Some of the proteins identified by mass spectrophotometry have already been recognized to modulate osteoclast biology but many others have not. It would be of interest to establish functionally which proteins might be having the effects observed because they could represent potential therapeutic targets.

## Bioengineering

## Regional segmentation of the murine bone cortex exposes spatial heterogeneity in lacunar: vascular organization

### Jacob Trend, Alisha Sharma, Patricia Goggin, Lysanne Michels, Heather Norman, Katrin Deinhardt, Phillipp Schneider, Claire Clarkin

#### University of Southampton, Southampton, UK

**Abstract**

Mechanical stressors are distributed heterogeneously across the bone cortex, and variation in regional cortical microarchitecture may reflect historical loading responses. Spatial alterations in bone microarchitecture have also been reported in skeletal pathology including osteoporosis.^(1)^ Although cortical microarchitecture comprises of allied networks including osteocyte lacunae and intracortical canals, the mechanisms driving their communication and organization remain poorly understood. Here, we describe an automated means to regionalize the bone cortex and characterize microstructural heterogeneity within the adult murine tibias using high‐resolution synchrotron radiation computed tomography (SR CT).

The tibia‐fibula junction (TFJ) of 13‐month‐old male mice (*n* = 3) was scanned postmortem by SR CT at 1.65 μm and an automated segmentation tool developed to isolate anterior, posterior, medial, and lateral regions, utilizing a combination of functions involving BoneJ's moment of inertia function within Fiji software. To investigate regional interactions between vascular canals and the lacunar network, 3D distance mapping tools were also developed within Dragonfly 2021.1. Consistent with literature,^(2)^ the posterior region exhibited greater canal density (0.23 per/100 μm^10‐3^ ± 0.11) versus the anterior (0.07 per/100 μm^10‐3^ ± 0.02), lateral (0.065 per/100 μm^10‐3^ ± 0.002), and medial (0.072 per/100 μm^10‐3^ ± 0.006) regions (*p* = 0.02; one‐way ANOVA). Osteocyte lacunae were also largest in the posterior region (652.7 μm^3^ ± 47.01) versus anterior (338.6 μm^3^ ± 64.03), and lateral (410.0 μm^3^ ± 14.7) regions (*p* = 0.003; one‐way ANOVA).

Evidence suggests that diffusion from vascular supply should support osteocyte survival to a distance of 100 μm.^(3)^ Computational 3D mapping of osteocyte lacunae distances from a vascular source comprising either (i) endosteal surface, (ii) periosteal surface, or (iii) intracortical canals across the TFJ showed that 6.4% ± 0.44% of lacunae were located >100 μm from a site of vascular supply. When distance mapping was performed in the absence of intracortical canals 26.8% ± 2.9% of lacunae within the posterior region were positioned >100 μm from a bone surface, suggesting that in this region a distinct subpopulation of osteocytes are “reliant” upon the vascular canals for survival. Furthermore, these “reliant” osteocyte lacunae found that they were significantly larger around the posterior intracortical canals (918.6 μm^3^ ± 47.7) versus lacunae in the anterior (429.3 μm^3^ ± 126.4, *p* < 0.05) and lateral (494.2μm^3^ ± 41.8, *p* < 0.05) regions.

Our study demonstrates heterogeneity in the bone vascular microstructure that is aligned to osteocyte lacunae size and spatial arrangements, and stresses the importance for regional study of cortical heterogeneity for optimal assessment of bone health and fracture risk.

**References**

1. Barger‐Lux MJ, Recker RR. Bone microstructure in osteoporosis: transilial biopsy and histomorphometry. *Top Magn Reson Imaging*. 2002;13(5):297‐305. DOI: 10.1097/00002142‐200210000‐00002

2. Núñez JA, Goring A, Javaheri B, et al. Regional diversity in the murine cortical vascular network is revealed by synchrotron X‐ray tomography and is amplified with age. *Eur Cell Mater*. 2018;35:281‐299. DOI: 10.22203/eCM.v035a20

3. Zahm AM, Bucaro MA, Ayyaswamy PS, et al. Numerical modeling of oxygen distributions in cortical and cancellous bone: oxygen availability governs osteonal and trabecular dimensions. *Am J Physiol Cell Physiol*. 2010;299(5):C922‐C929. DOI: 10.1152/ajpcell.00465.2009

## Cold atmospheric plasma as a potential treatment for diabetic foot osteomyelitis

### Dominic T Beith ^1,2^, Nishtha Gaur^2,3^, Jemma G. Kerns^1,2^, Rob D Short^2,3^

#### ^*1*^ *Lancaster* *Medical School, Faculty of Health and Medicine, Lancaster University, Lancaster, UK.* ^*2*^ *Material* *Science Institute, Lancaster University, Lancaster, UK.* ^*3*^ *Chemistry* *Department, Faculty of Science and Technology, Lancaster University, Lancaster, UK*

**Abstract**

Every week 75,000 diabetic foot ulcers (DFUs) are reported in the UK. DFUs are attributable for 80% of lower limb amputations, with 7000 amputations per year.^(1)^ The majority of DFUs at presentation are infected with polymicrobial biofilms, and 50% of severely‐infected DFUs exhibit osteomyelitis.^(2)^

Plasma medicine aims to address the issue of DFUs using the action of cold atmospheric plasma (CAP)—an ionized gas. CAP has the potential to destroy biofilms through the production of reactive oxygen and nitrogen species (RONS). CAP‐delivered RONS also have the potential to improve wound healing. Given the potential benefits, CAP devices are being trialed in DFUs.^(3)^

The aim of this study is to review the current clinical trial landscape on the use of CAP in DFUs and map to osteomyelitis. PubMed and Cochrane databases were searched using the keywords “cold atmospheric plasma,” “diabetic foot” and “randomized clinical trial”. From the 10 papers yielded, three duplicates were removed before screening titles and abstracts; three were included.

The randomized clinical trials (RCTs) compared standard/placebo treatment against CAP. The addition of CAP produced a statistically significant fractional decrease in wound size (Table 1). One RCT did not measure wound size, but showed that CAP decreases inflammatory cytokines.^(4)^

Our study shows that none of the RCTs conducted to‐date include diabetic foot osteomyelitis, highlighting a need for future RCTs. With a limited number of clinical trials, the improved patient outcomes and non‐antibiotic approach have raised confidence in the potential of CAP for DFUs.

**References**

1. National Institute for Health and Care Excellence (NICE). Diabetic foot problems: prevention and management. NICE Clinical Guidelines. 2019. NICE guideline [NG19]. Published: 26 August 2015. Last updated: 11 October 2019. https://www.nice.org.uk/guidance/ng19/chapter/Recommendations#treatment-2.

2. Giurato L, Meloni M, Izzo V, Uccioli L. Osteomyelitis in diabetic foot: a comprehensive overview. *World J Diabetes*. 2017;8(4):135‐142.

3. Stratmann B, Costea TC, Nolte C, et al. Effect of cold atmospheric plasma therapy vs standard therapy placebo on wound healing in patients with diabetic foot ulcers: a randomized clinical trial. *JAMA Netw Open*. 2020;3(7):e2010411.

4. Amini M, Sheikh Hosseini M, Fatollah S, et al. Beneficial effects of cold atmospheric plasma on inflammatory phase of diabetic foot ulcers; a randomized clinical trial. *J Diabetes Metab Disord*. 2020;19(2):895‐905.

5. Mirpour S, Fathollah S, Mansouri P, et al. Cold atmospheric plasma as an effective method to treat diabetic foot ulcers: a randomized clinical trial. *Sci Rep*. 2020;10(1):10440.

**Table:** **Table 1**. Fractional decrease in wound size after CAP

Author(s)	Wounds receiving CAP (*n*)	Wagner grade	Fractional decrease in wound size (%)
Stratmann et al.^(3)^	33	1/2	30.5*
Amini et al.^(4)^	68	2	‐
Mirpour et al.^(5)^	22	2	39*

* Denotes a statistically significant (*p* < 0.05) reduction.

## Cellular and Molecular

## Osteoclast fusion, size, and activation are regulated by extracellular pH

### Bethan Davies ^1^, Mark Hopkinson^1^, Gill Holdsworth^2^, Tim Arnett^3^, Isabel Orriss^1^

#### ^*1*^ *Royal* *Veterinary College, London, UK.* ^*2*^ *UCB* *Pharma Ltd., Slough, UK.* ^*3*^ *University* *College London, London, UK*

**Abstract**

Extracellular pH is a known modulator of osteoclast function. Acidosis directly stimulates bone resorption with near‐maximal effects at pH 7.0, whereas ≥pH 7.4 substantially limits osteoclast activity. This study investigated the mechanistic actions of low pH on osteoclast fusion and resorption. Mouse bone marrow–derived osteoclasts were cultured on dentine discs at pH 7.4 or pH 6.9 for 5 days. Osteoclast formation and activity were measured by image analysis of tartrate‐resistant acid phosphatase (TRAP)‐stained discs. The effect of pH on gene and protein expression and nuclei number was investigated using qPCR, Western blotting, and immunofluorescence, respectively.

Osteoclast number was ≤1.9‐fold (*p* < 0.001) higher in cells cultured at pH 6.9 compared to pH 7.4 osteoclasts. Extensive resorptive activity was observed in pH 6.9‐cultured osteoclasts; the level of resorption in pH 7.4 osteoclasts was 85% lower (*p* < 0.001). Culture at pH 7.4 was associated with the formation of larger, more nucleated osteoclasts (≤140 μm with 23 ± 15 nuclei/cell), whereas cells at pH 6.9 were ≤35 μm in size with 8 ± 3 nuclei/cell (*p* < 0.01). mRNA expression of osteoclast formation (eg, RANK, c‐FMS, TRAF6) and resorption genes (eg, cathepsin K, carbonic anhydrase II) was decreased in pH 7.4 osteoclasts. At the protein‐level, pH 7.4 osteoclasts unexpectedly expressed 1.5‐fold more cathepsin K (*p* < 0.01); however, enzymatic activity was ∼70% greater in resorbing pH 6.9 osteoclasts. Protein expression of the fusion marker DC‐STAMP was 1.5‐fold greater in pH 7.4‐cultured osteoclasts compared to pH 6.9; no differences were observed at the mRNA‐level.

Short‐term acid exposure of pH 7.4 osteoclasts reduced osteoclast size by 30% 4 hours post‐acidification. Osteoclast numbers increased 2.3‐fold by 24‐hours, potentially indicating fission. Resorptive activity is visible at 8 hours and is extensive 24 hours post‐acidification. Acidification of osteoclasts originally cultured at pH 7.4 results in a near similar resorptive activity by 24 hours to osteoclasts continually cultured at pH 6.9 (*p* < 0.05). Taken together, the current work indicates that extracellular pH modulates osteoclast fusion and size and may prime cells for subsequent resorptive activity.

## Activated CD4 T cells from the bone marrow participate in type 2 diabetes bone fragility

### Saul Ernesto Cifuentes‐Mendiola , Arnulfo Martinez‐Davalos, Ana Lilia Garcia‐Hernandez

#### Universidad Nacional Autonoma de Mexico, Ciudad de Mexico, Mexico

**Abstract**

The mechanisms leading to the development of osteopathy in type 2 diabetes mellitus (T2D) are currently unknown. The presence of activated T cells in bone marrow may take part in diabetic osteopathy by inducing an inflammatory microenvironment in bone tissue, which leads to osteoblast and osteoclast dysfunction and conduces to increased bone fragility. Because of this, we focused on determining the participation of activated bone marrow T cells in microstructural alterations and bone fragility in a mouse model of T2D.

A model of T2D was developed in C57BL/6 male mice with a hypercaloric diet and low doses of streptozotocin; we inhibited T cell activation with CTLA4‐Ig. We evaluated the glycemic profile during the experimental time (4 to 24 weeks of age), serum cytokine concentration by cytometric bead array (CBA) flow cytometry, T cell activation in bone marrow was determined by CD69 surface marker; tumor necrosis factor α (TNF‐α) and interleukin 17 (IL17) production by flow cytometry, TNF‐α concentration in bone tissue were determinate by ELISA; bone microarchitecture was evaluated by μCT and fracture resistance by three‐point assay. Our results showed that inhibition of T lymphocyte activation decreased blood glucose levels, insulin resistance, and serum pro‐inflammatory cytokines; reduced TNF‐α in bone and improved mineral density decreased trabecular bone porosity and increased resistance to fracture in comparison with diabetic mice without the inhibitor.

We conclude that activated bone marrow CD4+ T cell participates in microstructural alterations and bone fragility in T2D, probably by the production of TNF‐α in bone tissue.

## Targeting age‐related multi‐morbidities with bisphosphonates

### Helen Knowles ^1^, James Dunford^1^, Hal Ebetino^2^, Graham Russell^1,3^, James Edwards^1^

#### ^*1*^ *University* *of Oxford, Oxford, UK.* ^*2*^ *University* *of Rochester, Rochester,* *USA* *.* ^*3*^ *University* *of Sheffield, Sheffield, UK*

**Abstract**

The bisphosphonate class of drugs is well‐established and widely used in the treatment of excessive bone loss. The strong calcium‐binding affinity of bisphosphonates (BPs) was largely thought to direct biological effects to mineralized tissues, with bone‐resorbing osteoclasts as the primary target. Recent clinical observations suggest a range of unanticipated beneficial effects following BP treatment, including a reduction in cardiovascular events, cancer occurrence, and overall mortality. Interestingly, nitrogen‐containing but not non‐nitrogen‐containing BPs are associated with improved overall survival.

We have investigated effects of different concentrations of nitrogen‐containing BPs (zoledronate, alendronate, risedronate), non‐nitrogen‐containing BPs (clodronate, etidronate), and novel nitrogen‐containing BPs with modified binding affinity and biological activity (lidadronate, OX14) on a panel of cell lines from a broad range of cell types (monocyte [THP1], T cell [Jurkat], cardiomyocyte [HL1], fibroblast [MRC5], kidney [HEK]). Cell number (cell confluence) and cell death (Sytox green staining) were measured following exposure to 0.1nM to 100μM BP for 3 to 7 days. Significant apoptosis was only seen with high doses (10–100μM) of nitrogen‐containing BPs, regardless of the duration of exposure, that was prevented by rescue of the mevalonate pathway with 10μM geranylgeraniol (GGOH). Interestingly, low doses of all BPs (0.1nM–1μM) reduced basal levels of cell death and increased cell proliferation in some cell types. This effect of BP action was not affected by treatment with GGOH.

Similar protective effects were observed in response to induced stress. All types of BP could protect against H_2_O_2_‐induced cell death in some cell types, via a mevalonate pathway‐independent mechanism. Interestingly, risedronate alone was able to protect against UV‐induced cell death.

This data suggests that BPs exert a broad protective effect on multiple cell types, protecting against apoptosis and increasing cell proliferation in both basal conditions and in response to apoptotic stimuli. Although the individual effects are small, long‐term exposure to low‐dose BP (as would be expected in extraskeletal sites) could be anticipated to reduce the adverse effects of environmental insults that accumulate with age, so promoting healthy aging.

Funded by UK‐SPINE.

## Analysis of the in vivo frequency and in vitro phenotypic stability of human skeletal stem/progenitor cells identified by podoplanin and CD146 expression

### Rhayra Dias ^1^, César Fontenelle^2^, Márcio Schiefer^2^, Pietro Mannarino^2^, Lourenço Pinto Peixoto^3^, Marcelo Almeida^3^, Maria Isabel Rossi^1^, Marcos Farina^1^, Danielle Bonfim^1^

#### ^*1*^ *Institute* *of Biomedical Sciences, Rio de Janeiro, Brazil.* ^*2*^ *Orthopedics* *Service, Clementino Fraga Filho University Hospital, Rio de Janeiro, Brazil.* ^*3*^ *Santa* *Martha hospital, Niterói, Brazil*

**Abstract**

Although the identity of the skeletal stem cells (SSCs) in humans is still a matter of debate, a new set of phenotypic markers was proposed to discriminate the SSCs and their progeny. SSCs would comprise the podoplanin (PDPN)+ CD146− CD164+ CD73+ subset; its downstream bone, cartilage, and stroma progenitor (BCSPs) would be identified by the simultaneous expression of PDPN and CD146, whereas the osteoprogenitors (OPs) and chondroprogenitors (CPs) would be found in the PDPN− CD146+ and PDPN+ CD146− subsets, respectively.

Envisioning the optimization of cell products for use in bone repair strategies, we investigated the frequency of the aforementioned populations in the human adult bone marrow, the stability of their immunophenotypic profiles in vitro, and how these profiles correlate with that of bone marrow stromal cells (BMSCs). Following approval (n° 21768719.0.0000.5257), 15 surgical waste bone/marrow samples were collected from patients of both sex, older than 18 years, undergoing primary hip arthroplasty. After leukocyte and endothelial cell depletion, the frequencies of SSCs, BCSPs, OPs, and CPs were determined by fluorescence‐activated cell sorting (FACS).

Our analysis showed that the BCSPs were 3.12% ± 3.67% of the CD45− CD31− population; the CPs were 13.40% ± 10.14%; the OPs were 7.68% ± 11.01%; whereas SSCs accounted for 0.23% ± 0.26% of the cells. Due to the low frequency of the SSCs, we sorted the cells into two major subsets: the PDPN− CD146+ pool, which included the OPs; and the PDPN+ CD146−, which included the SSCs and the CPs. Both subsets were in vitro expanded and serially evaluated by FACS. BMSCs were used for comparison. As soon as the first passage, all cells in the PDPN− CD146+ population had acquired PDPN expression. Similarly, the cells of the PDPN+ CD146− subset started to express CD146. Thereafter, the expression of PDPN progressively decreased in both populations; and at the end of passage three, the phenotypic profiles of both populations were similar to that of BMSCs.

Therefore, the in vitro modulation of PDPN and CD146 in the sorted populations and the convergence of their profiles toward that of BMSCs suggest that the use of these markers might not be useful for the manufacture of cell products with better and predicted regenerative properties. Further transcriptomic and functional analysis will be performed to determine the relationship of the populations identified by PDPN and CD146 in vivo versus in vitro and versus BMSCs.

## Potential interactions between SQSTM1, KEAP1, and PARK2 in Paget's disease of bone

### Yvette Oppong ^1^, Marc Hansen^2^

#### ^*1*^ *University* *of Connecticut, Storrs,* *USA* *.* ^*2*^ *UConn* *School of Medicine, Center for Molecular Oncology, Storrs,* *USA*

**Abstract**

Paget's disease of bone (PDB) is a metabolic disease resembling excessive bone remodeling causing disorganized, structurally weak bone. The genetic etiology of PDB is heterogeneous, but a significant portion is attributed to mutations in the SQSTM1 gene. The SQSTM1 gene product (p62) is a 62‐kD protein linked to a number of cellular processes including autophagy. KEAP1 and PARK1 are proteins shown to interact with p62 during autophagy.

The hypothesis is that mutations in SQSTM1 associated with PDB could alter KEAP1 and/or PARK2 interaction with p62. Wild‐type (hFOB1.19) and pagetic (PSV10) osteoblast cell lines were treated with a combination of Vitamin D (VitD) and lipopolysaccharide (LPS) with or without simvastatin, a drug that blocks p62 activity in autophagy. The cells were then fixed and analyzed by immunofluorescence using antibodies against RELA, KEAP1, or PARK2. Activation of RELA (a known downstream target of p62 activation), KEAP1, and PARK2 were detected by changes in cellular localization of these proteins to the nucleus. Results showed activation of RELA in the PSV10 but not hFOB1.19 cells in response to LPS and VitD as measured by nuclear accumulation. Simvastatin addition appeared to eliminate this activation.

There was also nuclear accumulation of KEAP1 in the PSV10 cells following LPS and VitD stimulation, which was again blocked by simvastatin addition. However for PARK2, although there was nuclear accumulation in the PSV10 cells following LPS and VitD stimulation, simvastatin addition did not eliminate the nuclear localization of PARK2. In conclusion, VitD and LPS appeared to activate RELA, KEAP1, and PARK2 in PSV10 cells but not hFOB1.19 cells. Simvastatin appeared to suppress activation of RELA and KEAP1 but not PARK2. This suggests that mutations in SQSTM1 associated with PDB may play different roles in activating RELA, KEAP1, and PARK2 and that simvastatin affects only some of those roles.

## Aberrant paracrine signaling underlies the mutant histone‐driven giant cell tumor of bone

### Lucia Cottone ^1^, Lorena Ligammari^1^, Helen J Knowles^2^, Hang‐Mao Lee^3^, Stephen Henderson^1^, Sara Bianco^1^, Christopher Davies^4^, Fernanda Amary^4^, Ana Paula Leite^1^, Roberto Tirabosco^4^, Paul O′Donnell^4^, Kristian Haendler^3^, Joachim L Schultze^3^, Javier Herrero^1^, Agamemnon E Grigoriadis^5^, Paolo Salomoni^3^, Adrienne M Flanagan^1^

#### ^*1*^ *University* *College London, London, UK.* ^*2*^ *University* *of Oxford, Oxford, UK.* ^*3*^ *German* *Center for Neurodegenerative Diseases (* *DZNE* *), Bonn, Germany.* ^*4*^ *Royal* *National Orthopaedic Hospital (* *RNOH* *), London, UK.* ^*5*^ *King* *'s College London, London, UK*

**Abstract**

Oncohistones represent compelling evidence for a causative role of epigenetic perturbations in cancer. Giant cell tumors of bone (GCTs) are characterized by a mutated histone H3.3 as the sole genetic driver present in bone‐forming osteoprogenitor cells but absent from the osteoclasts, which represent a conspicuous component of the tumor microenvironment (TME). Denosumab treatment depletes GCT of osteoclasts, inducing growth arrest and maturation of tumor cells, suggesting a close crosstalk between the oncohistone‐bearing osteoprogenitor cells and osteoclasts in GCT pathogenesis.

To dissect the role of the oncohistone and to investigate the mechanisms of osteoclast recruitment in the GCT, we used in vitro models of human mesenchymal stem cells and osteoblasts in which we stably expressed mutant H3.3 (H3.3 G34W) as well as an in vitro human osteoclast assays and patient samples.

Here, we demonstrate that the H3.3 G34W mutation confers no direct growth advantage on mutant osteoprogenitors. We show that H3.3 G34W induces changes in the epigenome and transcriptome in mesenchymal cells which are sufficient to increase osteoclast recruitment. Osteoclasts in GCT are in part brought about by reduced expression of a transforming growth factor β (TGFβ)‐like soluble factor, SCUBE3, which has previously been implicated in bone biology. Osteoclasts in turn secrete semaphorin‐4D (SEMA4D), which promotes maturation arrest and proliferation of mutated H3.3 osteoprogenitors. In contrast, malignant GCTs develop autonomous growth, often transforming into osteosarcomas, and no longer respond to osteoclast‐depleting treatment.

We provide here a mechanism for GCT initiation and its response to denosumab treatment. More generally, this study emphasizes the importance of the TME in tumorigenesis.

## Clinical Cases

## Bone health in pheochromocytoma/paraganglioma

### VP Jyotsna ^1^, Saurav Khatiwada^1^, S Agarwal^2^, D Kandasamy^3^, R Kumar^4^, R M Pandey^5^, N Tandon^1^

#### ^*1*^ *Dept* *of Endocrinology, All India Institute of Medical Sciences, New Delhi, India.* ^*2*^ *Dept* *of Pathology, All India Institute of Medical Sciences, New Delhi, India.* ^*3*^ *Dept* *of Radiology, All India Institute of Medical Sciences, New Delhi, India.* ^*4*^ *Dept* *of Urology, All India Institute of Medical Sciences, New Delhi, India.* ^*5*^ *Dept* *of Biostatistics, All India Institute of Medical Sciences, New Delhi, India*

**Abstract**

**Introduction:** This study reports prevalence of low bone mineral density (BMD)/osteoporosis, areal BMD (aBMD), and trabecular bone score (TBS)‐adjusted Fracture Risk Assessment Tool (FRAX) score in subjects with paraganglioma (PPGL) and postoperative change at 3 to 15 months of follow‐up.

**Methods:** A total of 32 consecutive cases of pheochromocytoma/PPGL were taken. Provisional diagnosis was made on imaging, supported by biochemical evidence of catecholamine excess but confirmation of diagnosis was done histopathologically.

Subjects underwent focused history‐taking (including FRAX questionnaire), physical examination, documentation of tumor size, 24‐hour urinary catecholamines/metanephrines/vanillylmandelic acid (VMA) levels. Fasting serum samples for calcium, phosphate, creatinine, alkaline phosphatase, 25‐hydroxy vitamin‐D3, and intact parathyroid hormone (iPTH) were analyzed. BMD was measured by dual‐energy X‐ray absorptiometry (DXA) machine (Discovery A 84023; Hologic Inc., Marlborough, MA, USA). Lumbar spine TBS was obtained from DXA images using TBS iNsight software (version 3.0.2.0; Medimaps, Merignac, France) based on pixel‐level variations in DXA images. This software is calibrated with the DXA machine. BMD and TBS of spine were done preoperatively. Subjects with low BMD/osteoporosis or low vitamin‐D levels were routinely supplemented with calcium and vitamin D by hospital protocol.

Both DXA for BMD and TBS were repeated postoperatively at 3 to 15 months to see change among subjects who had complete excision of tumor.

International Society for Clinical Densitometry (ISCD) 2019 nomenclature was used for defining BMD categories. aBMD‐adjusted FRAX and TBS‐ and aBMD‐adjusted FRAX of subjects (>40 years of age) adjusted for Indian subjects were calculated individually online by entering data in Sheffield University website for FRAX calculation.

**Results:** Average age of subjects was 41 ± 13 years. A total of 18 of 32 (56%) subjects had either “lower BMD than expected for age” or osteoporosis.

Although a statistically significant difference in aBMD‐adjusted FRAX versus TBS‐ and aBMD‐adjusted FRAX was present, clinically meaningful difference was not present as similar number of subjects qualified for anti‐osteoporotic measures by both.

Among the factors predicting low *Z*‐score expected for age or osteoporosis, every kilogram less weight was associated with 12% lower *Z*‐score (statistically significant). Higher 24‐hour VMA level and presence of hyperadrenergic spells showed trends toward lower *Z*‐score, without statistical significance. No association was noted with size of tumor or 25‐hydroxy Vit‐D3 level and iPTH levels. No statistically significant change in BMD or TBS at lumbar spine was noticed at a median follow‐up of 4 months postoperatively.

## Common MSK Disorders

## The link between bone loss and coronary artery disease prospective study about 46 cases

### Kawtar Nassar, Saadia Janani

#### Department of Rheumatology, Ibn Rochd University Hospital Center, Hassan II University, Faculty of Medicine and Pharmacy. Casablanca, Casablanca, Morocco

**Abstract**

**Background:** Both osteoporosis and cardiovascular disease are causes of morbidity and mortality. Several publications have shown a link between coronary heart disease and osteoporosis. Their coexistence was considered to be distinct age‐related conditions and was attributed primarily to aging and common risk factors including diabetes, dyslipidemia, and smoking.

**Purpose of the Study:** To assess the prevalence of densitometric bone loss in patients followed for coronary artery disease compared to control subjects.

**Methods:** This is a prospective, case‐control, longitudinal study of patients followed for coronary artery disease and control subjects. The exclusion criteria were patients with known bone diseases. The patients included have been assessed and monitored at bone disease unit in rheumatology department of Ibn Rochd University Hospital and were divided into two groups: The first one with coronary artery disease and the second control group with normal coronary angiography. All patients underwent a complete clinical examination, blood and urinary phosphocalcic explorations, metabolic assessment, and bone densitometry (DXA).

**Results:** The study included 46 patients: 24 women and 22 men. The mean age was 65.7 ± 6.5 years. Regarding past medical history: 34% were diabetic type II, 60% of whom were under insulin therapy; 21% were dyslipidemic (80% of whom were under statins); 32% were hypertensive; 17% were chronic smokers. Ten cases (21.7%) had osteoporosis, 19 (41.3%) had osteopenia, and 17 (37%) had normal bone mineral density. The prevalence of osteoporosis and osteopenia was significantly higher in group I compared to the control group (with significant *p* value).

**Discussion and Conclusion:** Numerous publications describe a link between bone fragility and cardiovascular disease. The mechanisms are multiple and still imperfectly understood. However, osteoprotegerin is one of the factors that may explain a pathophysiological link between bone fragility and the constitution of atheromatous plaque. It inhibits the receptor activator of nuclear factor κB–receptor activator of nuclear factor κB ligand (RANK‐RANKL) system, a strong osteoclast activator, and also plays a role in protecting vascular diseases. Its decrease during osteoporosis could explain the occurrence of coronary artery disease. Our study joined the literature results, showing the high frequency of bone loss among patients followed for coronary artery disease. The exact link cannot be confirmed given the comorbidities found in our population. It seems important to request DXA to patients followed for vasculopathies and a cardiac assessment to osteoporotic subjects.

## Predictors factors of falls among 444 patients admitted for bone assessment by DXA

### Kawtar Nassar, Saadia Janani

#### Rheumatology Department, Ibn Rochd University Hospital Hassan II, University of Medicine and Pharmacy. Casablanca‐MAROC, Casablanca, Morocco

**Abstract**

**Background:** Osteoporosis affects bone mineral density (BMD) and bone microarchitecture at the origin of an increased risk of fracture. The reduction in bone density assessed by dual‐energy X‐ray absorptiometry (DXA) and fall history represent the first two risk factors of non‐vertebral fracture after menopause.

**Study purpose**: Evaluation of predictors factors of falls among patients admitted for bone assessment by DXA.

**Methods:** Transversal and descriptive monocentric cohort study conducted for 24 months in 444 patients referred by physicians regularly use prescription of BMD by DXA. The realization of this exploration by the same DXA‐Hologic in the rheumatology department at the University Hospital Ibn Rochd was the criterion for entry into the study. All patients were interviewed on the same day on the risk factors for osteoporosis and fractures justifying the prescription of a BMD. The falls predictors factors was evaluated by multivariate logistic regression.

**Results:** Data included 410 women and 34 men. Most women were postmenopausal (90.2%). The average age was 59.3 years (σ = 12.6), 34% were ≥65 years. The mean body mass index (BMI) was at 27.6; 18.3% of patients had at least a history of falls in the previous 12 months and 22.5% a history of fracture after a low‐energy trauma; 42.6% were osteoporotic and 36.7% had osteopenia in at least one of these sites: lumbar spine, femoral neck, total hip.

Past history of fractures, walking aid, vision disturbances, rheumatoid arthritis, diabetes were the predictors of falls in our population (Table).

**Summary and conclusion:** The fall history is an independent risk factor for fracture. This risk is particularly important in case of fragility bone densitometry.

The clinical history and fall risk factors should be taken into consideration in the assessment of fracture risk and in the anti‐osteoporotic treatment strategy. Thus, falls seem to be a legitimate indication for DXA and as a parameter that deserves to be integrated into the assessment of fracture risk by the FRAX score.

**Table:** Multivariate logistic regression results: adjustment for predictors factors of falls

**Table:** 

Variables	OR (95% CI)	*p*
Age ≥65 years	0.986 (0.412–2.357)	0.974
Past history of fracture	4.271 (1.719–10.611)	0.02
Walking aid	11.214 (2.815–44.670)	0.01
Vision disturbances	8.587 (3.540–20.829)	<0.001
Rheumatoid arthritis	8.047 (2.218–29.192)	0.02
Diabetes	3.194 (1.217–8.382)	0.018
Corticosteroid ≥3 months	0.603 (0.156–2.331)	0.463
Smoking	0.241 (0.013–4.518)	0.341
Osteopenia (−1 ≤ *T*‐score <−2.5)	2.204 (0.875–5.552)	0.094

## Sarcopenia in postmenopausal women

### Kawtar Nassar, Ahlam Ajerouassi, Saadia Janani

#### *Department of Rheumatology, Ibn Rochd University Hospital Center, Hassan* *II* *University, Faculty of Medicine and Pharmacy. Casablanca, Casablanca, Morocco*

**Abstract**

**Background:** From the age of 50 years onward a progressive loss of muscle mass may be observed, it often leads to real sarcopenia. Age, sedentary lifestyle, and hormonal factors represent major risk factors. The decrease in muscle leads to a loss of function, an altered quality of life, and the increase of fracture risk. Sarcopenia has a high prevalence in postmenopausal women. The purpose of the study is to evaluate the presence or absence of sarcopenia in menopausal women by the skeletal muscle index (SMI).

**Methods:** Transversal and descriptive monocentric study conducted in postmenopausal women homogeneous regarding demographic and risk factors. All women underwent a dual‐energy X‐ray absorptiometry (DXA) scan, in the rheumatology department at Ibn Rochd University Hospital, with evaluation of the body mass composition. The index was calculated to assess the presence or absence of sarcopenia. The sarcopenia is retained when the SMI is <5.45 kg/m^2^ in women.

**Results:** Twenty postmenopausal women was including. The mean age was 63.5 ± 7.2 years. The mean body mass index (BMI) was 27.9 ± 4.7 m/kg^2^. There was no past medical history apart from fracture in 30%. The mean age of menopause was at 48.2 ± 6 years. The *T*‐score (m) at the lumbar spine was at −2.82 ± 0.97, femoral neck at −1.76 ± 0.6, and total hip at −1.5 ± 0.87. The evaluation of the whole body found the mean total leg fat mass at 4337 ± 1460 g, mean fat total leg mass at 8272 ± 1313 g, and the bone mineral content at 364 ± 60 g. The mean whole fat mass was 20,013 ± 7705 g, the mean fat whole mass was 44,576 ± 6010 g, and the whole bone mineral content was 2061 ± 219 g. The total fat mass (kg)/taille2 (m) was at 18.99 ± 2.66 kg/m^2^. Finally, the skeletal muscle index was at 4.24 ± 0.76 kg/m^2^, correlated with sarcopenia.

**Discussion and Conclusion:** Sarcopenia has a high prevalence in postmenopausal women and it joins the results of our study. It affects at least 20% of the population from the age of 70 years and also obese subjects, posing multiple diagnostic problems (obesity‐sarcopenia syndrome). It is predominant in the lower limbs with a loss of muscle mass of around 15%. Sarcopenia is the consequence of multiple factors, which participate in its genesis, but also in its progression: neuronal, hormonal factors, lifestyle, vitamin D deficiency. The evaluation and prevention of sarcopenia are important in the management of postmenopausal women by acting on risk factors, muscle strengthening and prevention of falls and fractures.

## Association between spondyloarthritis activity and body composition

### Kawtar Nassar, Ahlam Ajerouassi, Saadia Janani

#### Department of Rheumatology, Ibn Rochd University Hospital Center, Hassan II University, Faculty of Medicine and Pharmacy. Casablanca, Casablanca, Morocco

**Abstract**

**Background:** Spondyloarthritis (SpA) is associated with altered body composition due to many factors such as inflammation and immobilization. Patients often present with low muscle mass and decreased strength with high fat mass. The muscle changes may occur in the early stages and persist throughout the disease duration like the excess of fat mass.

**Purpose:** To assess the link between the activity of SpA and the change of the fat and lean body mass.

**Method:** Transversal and descriptive monocentric study conducted in 20 SpA patients followed in the rheumatology department at the University Hospital Ibn Rochd. The study include patients with active disease and control group with remission disease. Demographic characteristics were noted in all patients as well as disease activity and treatment characteristics. All patients underwent a dual‐energy X‐ray absorptiometry (DXA) scan with evaluation of the body mass; precisely, the lean and the fat mass. Finally, the data of the two groups were compared to assess the relationship between disease activity and disturbances in body mass.

**Results:** A total of 20 patients were included. The mean age was 30 ± 10.3 years, with men predominant (85%). The mean body mass index (BMI) was 21.1 ± 4.7 m/kg^2^. Mean duration of primitive SpA was 11.4 ± 6.3 years. Mean sedimentation rate was 33.7 ± 26.8 mm/h and C‐reactive protein (CRP) 32.7 ± 33 mg/L. The mean ankylosing spondylitis disease activity score with CRP (ASDAS‐CRP) was 2.5 ± 1.2. Regarding the evaluation of body mass, we found the following results: total leg average fat mass 1067 ± 2012 g, total leg mean lean mass 10,757 ± 2568 g, and total leg bone mineral content (BMC) 391 ± 545 g. The whole‐body average fat was 6484 ± 13,054 g, the average lean whole body was 54,605 ± 9889 g, and the whole body BMC was 2673 ± 419 g (Table).

**Discussion and Conclusion:** The assessment of fat and lean mass is important in patients followed for chronic inflammatory rheumatism such as SpA. Inflammation induces an increase in fat mass and a decrease in lean mass with the risk of osteoporosis and fall, especially in patients with active rheumatism and obesity. Our study showed an increase in fat content in patients with active SpA and the decrease of the lean mass but without statistically significant difference with the control group. This can be explained by the low number of patients and the few associated comorbidities. The changes in body composition during SpA are linked to a cascade of metabolic abnormalities with inflammatory factors. There are multiple, complex interactions which remain largely unknown.

## PGSM and PCLMA polyHIPE scaffolds for osteochondral regeneration

### María Fernanda Velázquez de la Paz

#### The University of Sheffield, Sheffield, UK

**Abstract**

**Introduction:** Osteochondral (OC) defects are one of the most common musculoskeletal conditions in the UK. They are known for their low rate of healing and concomitant pathologies; they affect three of 10 citizens over the age of 45 years. Every year, the National Health Service (NHS) performs 100,000 hip and knee replacements; 90% of the patients are osteoarthritic. We propose an integrated biomaterial and tissue engineering (TE) approach as an early corrective solution.

**Methods:** PCL and PGS prepolymer solutions were synthesized through ROP and condensation reactions. Further methacrylation was developed through methacrylic anhydride. Prepolymer solutions were purified; H+NMR and GPC were performed as characterization. PolyHIPE emulsions were manufactured photocuring under UV light. Molding was used to create mono disks. SEM imaging, water contact angle and mechanical testing were performed. Early cell work was done on bulk and porous materials. BACs and hES‐MPs were seeded on PGSM and PCLMA scaffolds, respectively, to assess cytotoxicity, cell attachment, proliferation, early migration, and ECM production; the latter two were reviewed through fluorescent imaging and histology assays.

**Results:** High yield methacrylation was achieved by the control of number of free hydroxyl groups. Results showed photo‐responsive and thermo‐responsive polymers. DM was corroborated through NMR peaks on LM‐PCL (35%) and HM‐PCL (50%). PolyHIPEs were successfully manufactured through W/O emulsions. Emulsion stability was determined by experimenting with the type of solvent, speed and time of mixing, temperature of emulsion, and volume of internal phase. Porosity and pore sizes have been tailored for each TE application. Production of collagen and proteoglycans in chondral tissues, and collagen and calcium sulfates in bone, was observed. Material shrinkage reported for PCL‐HIPEs (15%–20%) and PGS (30%), and stability over 25 days. Shrinkage, and degradation assays were run on PBS and alkaline environments. Cell migration was measured with viability assays; an increase in proliferation reflected an increase in migration (Alamar Blue residues). Scaffolds were imaged through SEM, confocal and LightSheet; cellular‐like bodies were identified. Qualitative measures for collagen, calcium sulfates, proteoglycans, and alkaline phosphatase were developed in 2D and 3D cultures. Finally, the use of alginate hydrogels for cell encapsulation was successful for hES‐MPs after 7 days with no reported natural polysaccharide degradation.

**Conclusions:** PCLMA and PGSM have mechanical properties suitable for OC applications. Both polyHIPEs possess porous structures that allow cells to attach, proliferate and to slowly migrate through the scaffold. PCLMA‐ LM and PGSM 80% have shown better results for early cell attachment and proliferation.

## Fibula response to disuse: a longitudinal analysis in people with spinal cord injury

### Shima Abdelrahman ^1,2,3^, Mariel Purcell^2^, Sylvie Coupaud^1,2^, Alex Ireland^3^

#### ^*1*^ *Department* *of Biomedical Engineering, University of Strathclyde, Glasgow, UK.* ^*2*^ *Queen* *Elizabeth National Spinal Injuries Unit, Queen Elizabeth University Hospital, Glasgow, UK.* ^*3*^ *Research* *Centre for Musculoskeletal Science & Sports Medicine, Department of Life Sciences, Manchester Metropolitan University, Manchester, UK*

**Abstract**

Cross‐sectional studies suggest that the fibula responds differently to loading and disuse compared to the tibia, despite a similar loading environment. Although tibial bone changes following spinal cord injury (SCI) have been established in longitudinal studies, fibular changes remain unexplored.

In this study, fibular and tibial bone parameters were assessed in 13 individuals with SCI (aged 16–76 years). A set of peripheral quantitative computed tomography (pQCT) scans at 4%, 38%, and 66% distal‐proximal tibia length were acquired within 5 weeks (baseline) and at 12 months postinjury. Changes in total bone mineral content (BMC), bone cross‐sectional area (CSA), and bone mineral density (BMD) were assessed at the 4% site, and total BMC, total CSA, and cortical BMD and cortical CSA were analyzed at 38% and 66% in both bones using paired *t* tests. In addition, relationships between bone loss in the two bones at the same site were assessed using correlation and paired *t* tests.

At the 4% site, both total BMC and BMD declined over 12 months, but tibial BMC losses were greater than those observed in the fibula (−14.8% ± 12.4% and −6.9% ± 5.1%, respectively, *p* = 0.02). At the diaphyseal sites (38%, 66%), BMC reduced at both sites in the tibia, but only at the 66% site in the fibula, with that loss being larger in the tibia compared to the fibula (*p* = 0.03). The cortical BMD reduced by ∼2.5% in the tibia, but there was no change at either fibula site (both *p* > 0.4). Changes in bone CSA were similar between the two bones. No evidence of a correlation was found between individual changes in BMC between the two bones (all *p* > 0.25).

These results support cross‐sectional evidence of smaller disuse‐related bone changes in the distal fibula compared to the tibia, although in contrast to previous findings bone loss in the fibula shaft was observed. The lack of association between losses in the two bones, suggests that different mechanisms; eg, local mechanical or systemic metabolic changes, following injury might be responsible.

## The role of Tregs in osteoclast function in healthy and pathological aging

### Raquel Lopera‐Burgueno ^1^ , Bethan Davies^2^, Isabel Orriss^3^, Peter Barlow^1^, Katherine Staines^4^, Graham Wright^1^

#### ^*1*^ *Edinburgh* *Napier University, Edinburgh, UK.* ^*2*^ *University* *of London, London, UK.* ^*3*^ *Univeristy* *of London, London, UK.* ^*4*^ *Brighton* *University, Brighton, UK*

**Abstract**

Osteoarthritis has traditionally been considered a “non‐inflammatory” form of arthritis. However, there is increasing evidence to suggest that the characteristic articular cartilage degradation follows biochemical changes to the joint, and furthermore, there is clear evidence of immune cell invasion in the damaged joint. Regulatory T cells (Tregs) (CD4+Foxp3+) are a subpopulation of T helper cells thought to play a role in osteoclastic bone resorption; however, their role in osteoarthritis is unknown.

In order to determine the aging changes on Treg presence in peripheral blood, CD4+Foxp3+ Treg quantification was undertaken in peripheral blood obtained from healthy young and healthy old participants using flow cytometric analysis, as well as quantification of bone homing markers CXCR4 and CCR4 to determine the possibility of Treg migration toward bone. Moreover, the effects of Tregs on osteoclast activity was further analyzed. Primary human osteoclast cultures were established and co‐cultured with Tregs for up to 21 days. Osteoclast number, size, and resorption were quantified via tartrate‐resistant acid phosphatase (TRAP) staining. Additionally, localization of Tregs in a surgical murine model of osteoarthritis (destabilization of the medial meniscus) was analyzed by immunohistochemistry for the presence of Foxp3+.

Quantification of Tregs (CD4+Foxp3+) in peripheral blood by flow cytometry showed no significant difference in numbers when comparing healthy young with healthy old participants. However, the percentage of bone homing markers (CCR4 and CXCR4) expressed by CD4+Foxp3+ Tregs decreased with aging (62% in healthy young versus 40% in healthy old for CXCR4 and 42% in healthy young versus 28% in healthy old for CCR4 and, *p* < 0.05). Although preliminary data utilizing osteoclast co‐cultures showed co‐culture of osteoclasts with Tregs had no effect on the number of osteoclasts formed, a decrease in osteoclast activity was observed when these cells were cultured in the presence of Tregs, in comparison to osteoclasts alone. This data suggests that Tregs play a role in osteoclast function.

Together these data provide further evidence for Tregs to play a role in osteoclastic bone resorption, and this process to be disrupted by aging. Ongoing studies will determine the effects of osteoarthritis on these processes.

**Epidemiology**

## Ecological study of fractures in pediatric Melanesian communities with varying endemic environmental fluoride exposure

### Elizabeth Webb^1^, Ahmed Elmansouri^2^, Rebecca Ross^2^, Michael Clynes^2^, Carol Stewart^3^, Elaine Dennison ^1,2^

#### ^*1*^ *Victoria* *University, Wellington, New Zealand.* ^*2*^ *MRC LIfecourse* *Epidemiology Unit, Southampton, UK.* ^*3*^ *Massey* *University, Wellington, New Zealand*

**Abstract**

**Introduction:** Osteoporotic fracture is a major public health burden worldwide, leading to very significant mortality and morbidity. Studies that have reported bone health in areas of high endemic fluorosis have commonly reported adverse skeletal as well as dental effects. To date most of these studies have been conducted in India, Turkey, and Iran. Vanuatu, sited in the Pacific, has six continuous degassing volcanoes on separate islands, resulting in a natural experiment as an ecological study of relationships between naturally occurring fluoride exposure (F) and fracture incidence in pediatric populations.

**Methods:** This ecological study recruited 1029 participants, lifetime residents of rural Vanuatu islands. Research sites were selected to represent differing fluoride exposure distance from the active volcanic cones and known high rate of fluoride in the local environment. A short questionnaire was administered detailing gender, age, and residential history. Participants were asked if they had broken a bone, and if so were asked to mark its location on a questionnaire manikin. Dental fluorosis was assessed as Dean's index. Community drinking‐water samples were sampled for fluoride concentration.

**Results:** Measured water fluoride concentration and recorded dental fluorosis displayed expected gradients from Aneitym (low) to Ambrym (high) (*p* < 0.001, Table 1). However, highest self‐reported fracture rates were recorded in the area with medium fluoride levels (Lamap/Uliveo), where 14.9% boys and 15.6% girls reported a fracture. On Ambrym, where the mean age of participants was similar, corresponding fracture rates were 4.5% and 2.6% (*p* value for difference all *p* < 0.05).

**Conclusions:** In this ecological study we have reported self‐recorded fractures among children living on three islands in Vanuatu with very different fluoride concentrations. Reports of fractures were common in these children, but demonstrably higher on Lamap, the region with medium fluoride concentrations, than on Ambrym, an island with very high rates of naturally occurring fluoride levels. Longer‐term studies that report validated fracture after peak bone mass acquisition are required.

**Table:** **Table 1** Summary statistics for study population

	Aneitym		Lamap/Uliveo		Ambrym	
Parameter	Boys (*n* = 118)	Girls (*n* = 74)	Boys (*n* = 255)	Girls (*n* = 269)	Boys (*n* = 110)	Girls (*n* = 115)
Mean water F (mg/L)	0.05	0.05	0.47	0.47	1.20	1.20
Age (years), mean ± SD	8.6 ± 1.4	7.8 ± 1.2	10.3 ± 3.1	10.6 ± 3.7	9.53 ± 3.0	10.2 ± 3.3
Dental fluorosis (%)	0	0	25.8	25.8	56.4	56.4
Self‐reported fractures (%)	4.6	5.4	14.9	15.6	4.5	2.6

## Changes in BMD in South African women transitioning through menopause, and the impact of HIV infection: a longitudinal study

### Tafadzwa Madanhire ^1,2^, Lisa Micklesfield^1^, Kate Ward^1,3^, Julia H Goedecke^1,4^, Nicole Jaff^5^, Nigel Crowther^6^, Shane Norris^1^, Rashida Ferrand^2,7^, Andrea Rehman^8^, Celia Gregson^1,9^

#### ^*1*^ *SAMRC* */Wits Developmental Pathways for Health Research Unit, School of Clinical Medicine, Faculty of Health Sciences, University of the Witwatersrand, Johannesburg, South Africa.* ^*2*^ *Biomedical* *Research and Training Institute, Harare, Zimbabwe.* ^*3*^ *MRC* *Lifecourse Epidemiology, Human Development and Health, University of Southampton, Southampton, UK.* ^*4*^ *Non‐Communicable* *Diseases Research Unit, South African Medical Research Council, Cape Town, South Africa.* ^*5*^ *Department* *of Chemical Pathology, Faculty of Health Science, University of the Witwatersrand,, Johannesburg, South Africa.* ^*6*^ *Department* *of Chemical Pathology, Faculty of Health Science, University of the Witwatersrand, Johannesburg, South Africa.* ^*7*^ *Clinical* *Research Department, Faculty of Infectious and Tropical Diseases, London School of Hygiene and Tropical Medicine, London, UK.* ^*8*^ *MRC* *International Statistics and Epidemiology Group, Department of Infectious Disease Epidemiology, Faculty of Epidemiology and Population Health, London School of Hygiene and Tropical Medicine, London, UK.* ^*9*^ *Musculoskeletal* *Research Unit, Translational Health Sciences, Bristol Medical School, University of Bristol, Bristol, UK*

**Abstract**

**Background:** An estimated 25.8% of South African women live with HIV. Antiretroviral therapy (ART) rollout has improved life expectancy, so many more women now reach menopause, increasing osteoporosis risk. We aimed to quantify changes in bone mineral density (BMD) during menopausal transition, in urban‐dwelling South African women living with and without HIV and to determine whether HIV infection modifies the effect of menopausal transition on BMD loss.

**Methods:** A 5‐year population‐based longitudinal study recruited women aged 40 to 60 years resident in Soweto, Johannesburg, and collected demographic data, clinical history including HIV status, anthropometry, and BMD, at baseline and follow‐up. All women were staged into one of three menopausal categories (premenopause, perimenopause, and postmenopause) at both time points. Multivariate linear regression assessed the relationships between HIV infection, menopause, and change in BMD (∆BMD), evaluating the interaction between HIV infection and menopausal transition on ∆BMD.

**Results:** At baseline, 450 women had mean ± standard deviation (SD) age 49.5 ± 5.7 years, 65% ± 14.4% had HIV, and 140% ± 31.1%, 119% ± 26.4%, and 191% ± 42.4%, were premenopausal, perimenopausal, and postmenopausal, respectively. After mean ± SD follow‐up of 4.8 ± 0.8 years, 38 (8.4%), 84 (18.7%), and 328 (72.9%) were premenopausal, perimenopausal, and postmenopausal. A body mass index (BMI) ≥30 kg/m^2^ was common, and seen more often in HIV‐uninfected women, than those with HIV (260/385 [67.5%] versus 33/65 [50.8%]; *p* = 0.002). Overall, at baseline 20 of 205 women age ≥50 years had HIV and 34 of 205 (13.6%) had a *T*‐score ≤−2.5 at either total hip (TH) or lumbar spine (LS).

At follow‐up, compared against HIV‐uninfected women, women with HIV had lost more total body (TB) BMD (mean difference −0.013; 95% confidence interval [CI], −0.026 to −0.001 g/cm^2^; *p* = 0.040) and gained more weight 1.96 [95% CI, 0.32–3.60 kg; *p* = 0.019. In multivariate analysis, after adjusting for age, weight, weight change, and follow‐up time, the transition from premenopause to postmenopause was associated with greater TB BMD losses in women with HIV (−0.092 [95% CI, −0.042 to −0.142] g/cm^2^; *p* = 0.001) than in women without HIV (−0.038 [95% CI, −0.016 to −0.060] g/cm^2^; *p* = 0.001), interaction *p* = 0.034. Similarly, in women postmenopausal throughout, those with HIV lost more TB BMD (−0.070 [95% CI, −0.031 to −0.108]; *p* = 0.001), than women without HIV (−0.036 [95% CI, −0.015 to −0.057]; *p* = 0.001), interaction *p* = 0.049. Findings were similar at the LS.

**Conclusion:** Menopause‐related bone losses appear greater in women with HIV. These findings suggest, women with HIV maybe at greater risk of developing osteoporosis when transitioning into menopause. This may increase future osteoporosis‐related fracture risk. South African HIV services should consider routine bone health assessment in menopausal women as part of long‐term HIV care delivery.

## Skeletal familial associations as measured by pQCT, HR‐pQCT, and DXA

### Camille Parsons ^1^, Nicholas Fuggle^1^, Mícheál Ó Breasail^2^, Michael Clynes^1^, Gregorio Bevilacqua^1^, Sarah Carter^1^, Elaine Dennison^1,3^, Cyrus Cooper^1,3,4^, Kate Ward^1,2^

#### ^*1*^ *MRC* *Lifecourse Epidemiology Unit, University of Southampton, Southampton, UK.* ^*2*^ *MRC* *Nutrition and Bone Health Research Group, Cambridge, UK.* ^*3*^ *National* *Institute for Health Research Biomedical Research Centre, University of Southampton and University Hospital Southampton* *NHS* *Foundation Trust, Southampton, UK.* ^*4*^ *National* *Institute for Health Research Musculoskeletal Biomedical Research Unit, University of Oxford, Oxford, UK*

**Abstract**

**Background:** A familial association of areal bone mineral density (aBMD), the gold standard for diagnosis of osteoporosis, has been reported in several studies. However, bone microarchitecture, as well as BMD, contributes to fracture risk, and previous work has considered relationships in mother‐offspring pairs when the offspring were young adults. The aim of this study was to explore parent‐to‐child associations in bone size, geometry, and microarchitecture using data collected from the Hertfordshire Cohort Study (HCS).

**Methods:** Data from across three generations of community‐dwelling HCS study participants were collapsed into parent‐to‐child pairs to maximize statistical power. All participants underwent peripheral quantitative computed tomography (pQCT, Stratec XCT2000L), high‐resolution pQCT (HR‐pQCT, XTreme I, Scanco), and dual‐energy X‐ray absorptiometry (iDXA, GE‐Lunar) (hip, spine) scans. Images of the non‐dominant radius and tibia were obtained from pQCT and HR‐pQCT. Due to lack of clustering within family lines, linear regression was used to explore associations between parent‐to‐child bone parameters, adjusting for age of parent and child, sex of parent and child, child's social class, and child's height. Results are presented as β (95% confidence interval [CI]).

**Results:** Parents (*n* = 61) had a mean age of 69 years (range, 49 to 88 years) and 74% (*n* = 45) were women. Children (*n* = 64) had a mean age of 42 years (range, 18 to 65 years) and 72% (*n* = 46) were women. Positive parent‐to‐child associations were found between tibial total volumetric BMD (vBMD, mg/cm^3^) at the 4% slice (0.47 [95% CI, 0.15–0.79]), and tibial cortical area (mm^2^), cortical thickness (mm), and total area (mm^2^) at the 38% slice (0.52 [95% CI, 0.24–0.81], 0.37 [95% CI, 0.10–0.64], and 0.30 [95% CI, 0.01–0.60], respectively) obtained from pQCT scans. Positive parent‐to‐child associations were also observed for tibial trabecular vBMD (mg/cm^3^), tibial trabecular number (mm^−1^) and tibial trabecular thickness (mm) (0.49 [95% CI, 0.11–0.88], 0.28 [95% CI, 0.01–0.54], and 0.32 [95% CI, 0.01–0.63], respectively) obtained from HR‐pQCT scans. These findings are consistent with iDXA‐measured aBMD, with positive parent‐to‐child associations in BMD (g/cm^2^) observed at the total hip and lumbar spine (0.41 [95% CI, 0.13–0.70] and 0.47 [95% CI, 0.26–0.69], respectively).

**Conclusions:** This is the first study using pQCT, HR‐pQCT, and iDXA images in parents and children. Our results demonstrated a familial association in vBMD, likely due to associations in trabecular microarchitecture and cortical bone parameters. These results provide further evidence of familial associations in bone size, strength, and geometry, extending previous work reporting relationships in mothers and young adult offspring.

## Applicability and agreement of two methods of bone age assessment in Zimbabwean children

### Farirayi Nyakoko ^1,2,3^, Celia Gregson^4,5^, Andrea Rehman^6^, Tafadzwa Madanhire^2^, Lynda Stranix‐Chibanda^3^ , Ruramayi Rukuni^2,6^, Amaka Offiah^7^, Lisa Micklesfield^5^, Rashida Ferrand^2,6^, Cyrus Cooper^1^, Kate Ward^1^

#### ^*1*^ *University* *of Southampton, Southampton, UK.* ^*2*^ *Biomedical* *Research Training Institute, Harare, Zimbabwe.* ^*3*^ *University* *of Zimbabwe, Harare, Zimbabwe.* ^*4*^ *University* *of Bristol, Bristol, UK.* ^*5*^ *University* *of the Witwatersrand, Johannesburg, South Africa.* ^*6*^ *London* *School of Hygiene and Tropical Medicine, London, UK.* ^*7*^ *University* *of Sheffield, Sheffield, UK*

**Abstract**

**Introduction:** Bone age (BA) measurement in children is important to identify growth delay. The two most used methods, (Greulich and Pyle [GP] and Tanner and Whitehouse [TW]), are based upon assessment of a hand‐wrist radiograph. In sub‐Saharan Africa few studies have determined BA despite it being a region where childhood growth in children is often affected by, for example, HIV and malnutrition. We aimed to determine applicability and agreement between these two methods of BA assessment, and chronological age in Zimbabwean children and adolescents.

**Methods:** We conducted a cross‐sectional study of children aged 8 to 16.5 years who tested negative for HIV. Boys and girls were recruited by stratified random sampling from schools in Harare. Hand‐wrist radiographs were taken of the nondominant hand and BA assessed using both GP and TW3 methods. Paired sample Student's *t* tests were used to compare the mean differences between BA by GP (GPBA); by TW3 (TW3BA) and chronological age (CA) in boys and girls separately; data are presented as mean difference (95% confidence interval [CI]). Bland‐Altman plots assessed agreement between CA and BA as determined by both methods, and between GPBA and TW3BA.

**Results:** We recruited 230 children (102 girls). For girls, mean ± standard deviation (SD) CA was 11.72 ± 2.00 years; GPBA 11.58 ± 2.40 years; and TW3BA 11.94 ± 2.06 years. In boys, mean ± SD CA was 12.32 ± 2.44 years; GPBA 11.59 ± 2.90 years; and TW3BA 11.92 ± 2.48 years. CA and BA, by either method, was similar in girls (CA‐GP 0.19 [95% CI, −0.02 to 0.41] years; CA‐TW3 –0.17 [95% CI, −0.38 to 0.04] years). In contrast in boys, both GP and TW3 identified a younger BA than CA, suggesting that boys were skeletally less mature than their CA (CA‐GP 0.74 [95% CI, 0.53–0.93] years; CA‐TW3 0.40 [95% CI, 0.22–0.59] years). In both sexes, Bland‐Altman plots showed wider limits of agreement between CA‐GPBA than CA‐TW3BA. The agreement between CA and TW3BA did not show bias across the CA range studied, whereas the mean difference between CA and GPBA was positively biased toward higher BA values in younger children and lower BA values in older adolescents in both sexes (Fig. 1a,b). In both boys and girls, GP measured consistently lower than TW3 (girls −0.36[95% CI, −0.54 to −0.18] years; CA‐TW3 –0.33 [95% CI, −0.49 to −0.17] years).**Conclusion:** Zimbabwean boys were less skeletally mature than girls of the same age. The TW3 method is more applicable to the Zimbabwean population because TW3 BA was in closer agreement with CA and showed no systematic bias with CA. The TW3 and GP methods do not agree and therefore cannot be used interchangeably.

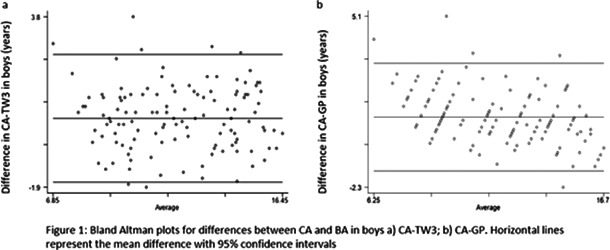

## Variability and predictors of CCG level dispensing of anti‐osteoporosis medication in 2019: An ecological study

### Sobia S Janjua ^1^, Arvind Sami^2^, Antony Johansen^3^, Helen Boardman^1^, Li Shean Toh^1^, M Kassim Javaid^2^

#### ^*1*^ *Division* *of Pharmacy Practice and Policy, School of Pharmacy, University of Nottingham, Nottingham, UK.* ^*2*^ *Nuffield* *Department of Orthopaedics, Rheumatology, and Musculoskeletal Sciences, Botnar Research Centre, University of Oxford, Oxford, UK.* ^*3*^ *University* *Hospital of Wales, Cardiff, UK*

**Abstract**

**Objective:** The role of the community pharmacist has been highlighted in services model to improve osteoporosis management. The use and type of anti‐osteoporosis medication (AOM) should reflect the risk of fragility fracture. We examined the variability and predictors of AOMs dispensed via community pharmacies in England at the Clinical Commissioning Group (CCG) level.

**Methods:** This ecological study used the Open Prescribing database to provide AOM items dispensed via community pharmacies in England in 2019. CCGs were mapped to Health Authority (HA) areas. Characteristics of the local HA population included proportion aged >65 years, life expectancy for women and men, current smoking, alcohol‐related hospital admissions, and percentage of active adults. Hip fractures admissions per CCG were extracted from the National Hip Fracture Database (NHFD). CCGs unable to be mapped to HA(s) were excluded. Atorvastatin was used as a comparator medication for long‐term conditions. CCGs with fewer than 100 reported hip fractures were excluded (*n* = 9). Independent predictors of AOM use were identified using regression methods. Quantile regression was used for the ratio between denosumab and alendronate dispensed.

**Results:** Of the 135 CCGs in England, 85 were mapped to HA areas with sufficient hip fractures. The median CCG population was 318,885 (interquartile range [IQR] 237,354–556,229), with an average of 17.6% aged ≥65 years. The rate of hip fractures per 1000 adults aged ≥65 years varied from 2.2 to 12.6 (median 5.9). The number of AOMs dispensed in 2019 per 1000 population aged ≥65 years varied between 379 and 1036 items (median 690). Neither smoking, reported activity, alcohol‐related admissions, or hip fracture admissions predicted total AOM dispensed after adjusting for age (*p* > 0.1). In contrast, amount of atorvastatin dispensed remained a significant (*p* < 0.001) positive predictor of total AOM dispensed even after adjusting for age. Excluding three CCGs with no community dispensing of denosumab, the ratio of denosumab versus alendronate dispensed varied from 0.01% to 9.6% (median 0.5%) across CCGs, with the proportion of adults reported as physically active the only independent predictor (*p* = 0.024) and no relationship with local hip fracture rates (Figure).

**Conclusion:** The amount and type of AOM dispensed by community pharmacies within a CCG varied considerably with few ecological predictors. Due to their accessibility, community pharmacists are ideally placed to play a vital role in osteoporosis management. Further work is needed to optimize the roles of community pharmacists to support effective and equitable osteoporosis management.

## Imaging

## Automated image segmentation for analysis of in vitro osteoclast endpoints

### Bethan Davies ^1^, Andrew Hibbert^1^, Mark Hopkinson^1^, Gill Holdsworth^2^, Isabel Orriss^1^

#### ^*1*^ *Royal* *Veterinary College, London, UK.* ^*2*^ *UCB* *Pharma Ltd., Slough, UK*

**Abstract**

Quantification of osteoclast culture endpoints (eg, osteoclast numbers, bone area) often relies on manual analysis methods. Although this approach enables user confirmation of osteoclasts and associated pits, it is labor‐intensive, extremely time‐consuming, and results in substantial user variability (coefficient of variation ≤40%). This study aimed to develop and validate an automated, machine learning (ML)‐based workflow to simultaneously, reliably, and robustly quantify osteoclast culture endpoints.

Historic images of tartrate‐resistant acid phosphatase (TRAP)‐stained mouse bone marrow–derived osteoclasts cultured on dentine discs were used to train the ilastik‐based ML algorithm. Assessment of algorithmic training revealed that osteoclast numbers and the total area resorbed strongly correlated between manual‐ and automatically‐quantified values (*r* = 0.75 and 0.83, respectively). Osteoclasts were faithfully segmented when visually compared to the original images. The accuracy of automated osteoclast number quantification was validated using zoledronate, a bisphosphonate with well‐characterized inhibitory effects on osteoclasts, and ticagrelor, a P2Y_12_ receptor antagonist with less well‐known effects on osteoclast biology. A 70% reduction (*p* < 0.01) in osteoclast number was detected, irrespective of quantification method, when cultured with 10nM zoledronate. Both methods also detected a dose‐dependent decrease in osteoclast number when treated with 1 to 10μM ticagrelor (*p* < 0.05). Development of the ilastik algorithm reduces user variability by ≤100% (*p* < 0.001).

Resorption pits were occasionally inaccurately identified using the ilastik algorithm; therefore, μCT‐based protocols quantifying bone resorption were also developed. Dentine discs with adherent osteoclasts were scanned at 2 μm; resorption area was visualized and quantified using CTvox and CtAn software, respectively. Three‐dimensional reconstructions reveal significant reductions in osteoclast resorptive activity following zoledronate treatment; μCT and manual quantification values were comparable.

Overall, an automated image segmentation and analysis workflow was developed and validated to consistently and sensitively identify osteoclasts, but not resorption pits. μCT analysis could provide an alternative solution to automate bone resorption analysis. This pipeline significantly reduces user variability of endpoint measurements and analysis time by 75%.

## A new method for segmentation and analysis of bone callus in rodent fracture models using μCT

### Mark Hopkinson ^1^, Gareth Jones^1^, Lucinda Evans^1^, Ran Magnusdottir^1^, Stephanie Gohin^1^, Chantal Chenu^1^, Phil Salmon^2^, Richard Meeson^1^, Behzad Javaheri^1^, Andrew Pitsillides^1^

#### ^*1*^ *Royal* *Veterinary College, London, UK.* ^*2*^ *Bruker* *, Kontich, Belgium*

**Abstract**

Increased fracture burden has created a need to better understand bone repair processes under different pathophysiological states. Evaluation of structural and material properties of the mineralized callus, which is integral to restoring biomechanical stability in indirect fracture repair, is, therefore, vital. μCT can facilitate noninvasive imaging of fracture repair within experimental settings; however, current methods for callus segmentation from cortical bone are only semiautomated, restricted to defined regions, time/labor intensive, and prone to user variation. Herein, we share a newly developed automatic method for segmenting callus from cortical bone in μCT tomograms that will allow for objective, quantitative analysis of the bone fracture microarchitecture.

Fractured and nonfractured mouse femurs were μCT scanned and then processed by both manual and automated segmentation of fracture callus from cortical bone after which the microarchitectural parameters were analyzed. Results showed that the automatic segmentation method could reliably and consistently segment callus from cortical bone with Pearson's correlation coefficient showing a strong linear relationship for bone and tissue volume measurements (*r* = 0.99, *p* = 0.0002, and *p* = 0.0004, respectively). When compared to manual segmentation, the newly developed method was faster (30 minutes manually versus 1 minute automatic) and eliminated user‐bias and variation (tissue volume % CV = 15.6%). Scalability and translatability of the method within rodent models was demonstrated using μCT scans of fractured rat femurs with varying gap sizes, which demonstrated advantages in extending evaluation to the entire fracture callus volume in our method.

Together, these data serve to validate a new automated method for segmentation of callus and cortical bone in μCT tomograms that we have developed, which we share as a fast, reliable, and less user‐dependent tool for application to the study of bone callus in fracture, and potentially elsewhere.

## Other

## Bone recovery after treatment: a feasibility study exploring changes in myeloma bone disease in patients receiving first line cancer treatment—is there a role for bone anabolics?

### Becky Andrews ^1,2^, Ingrid Jolley^2^, Janet Brown^1,2^, Michelle Lawson^1^, Andrew Chantry^1,2^

#### ^*1*^ *University* *of Sheffield, Sheffield, UK.* ^*2*^ *Sheffield* *Teaching Hospitals,* *NHS* *Foundation Trust, Sheffield, UK*

**Abstract**

**Background:** The majority of patients with a diagnosis of multiple myeloma (MM) experience myeloma bone disease (MBD), resulting in life‐changing symptoms such as chronic pain and poor mobility. Current bone‐targeted therapies available for the treatment of MBD are limited to bisphosphonates or denosumab, which aim to prevent further bone destruction, but do not repair the damage. We propose that use of bone anabolic therapies in MBD could significantly improve patient outcomes.

**Objectives:** The primary aim of this study is to evaluate the feasibility of assessing bone recovery in MM patients undergoing first‐line cancer treatment. The study will provide valuable information to design a larger‐scale interventional trial assessing bone anabolic treatments for MBD. Secondary exploratory objectives include comprehensive assessment of changes in bone architecture (macroscopic and microscopic), bone turnover, and alterations to the surrounding microenvironment indicative of bone recovery.

**Methods:** This is a prospective, observational, feasibility study, recruiting patients at the Royal Hallamshire Hospital (Sheffield, UK) with a new diagnosis of MM, for longitudinal assessment of bone health throughout (and following) their first‐line treatment. Monitoring includes bone marrow biopsies, fasting serum samples for bone turnover markers, whole‐body low‐dose CT imaging, and quality of life (QOL) questionnaires. Major follow‐up time points are planned at 3 months and 1 year post completion of treatment (+/− autologous stem cell transplant) and compared to baseline.

**Results:** By March 2020 there were 20 active participants (the original recruitment target). Unfortunately, the study was closed to data collection from March 2020 as a result of the coronavirus disease 2019 (COVID‐19) pandemic, and therefore multiple study time points have been lost. The study was recently approved to re‐open and awaits ethical approval to incorporate later follow‐up data collection points. Preliminary results demonstrate dramatic bone recovery for some study participants, evidenced on follow‐up CT imaging and bone marrow trephine microarchitecture, potentially due to the use of bortezomib chemotherapy in treatment regimes.

**Conclusions:** This study will evaluate the feasibility of a full‐scale early‐phase interventional trial assessing efficacy of bone anabolic agents in MBD, with particular focus on acceptability, implementation, and practicality of extensive bone monitoring alongside first‐line cancer‐targeting treatment. In addition, the study will provide exploratory outcomes to assess changes in the bone microenvironment during treatment.

**Ethical Permissions:** This study was reviewed by Yorkshire & The Humber ‐ Bradford Leeds Research Ethics Committee and approved by the Health Research Authority [REC18/YH/0275].

**Funding:** This project is funded by Weston Park Cancer Charity.

## A comparison of the associations between accelerometer‐derived moderate and vigorous intensities of physical activity and bone health outcomes in children and adolescents: a systematic review

### Gemma Brailey ^1^, Brad Metcalf^1^, Rebecca Lear^1^, Lisa Price^1^, Sean Cumming^2^, Victoria Stiles^1^

#### ^*1*^ *University* *of Exeter, Exeter, UK.* ^*2*^ *University* *of Bath, Bath, UK*

**Abstract**

**Purpose:** Accelerometer‐derived moderate (MPA) and vigorous (VPA) intensities of physical activity (PA) have been shown to have positive associations with bone outcomes in children and adolescents. However, it is unclear whether a particular intensity is more beneficial. This systematic review aimed to determine whether the magnitude of association between PA and bone outcomes was consistently stronger for a particular intensity of activity (MPA, MVPA, or VPA) and to summarize the accelerometry methods used to obtain PA data in these studies.

**Methods:** A systematic electronic search was conducted in MEDLINE, EMBASE, Web of Science, SPORTDiscus, and the Cochrane Central Register of Controlled Trials to identify observational studies that had assessed associations between accelerometer‐derived habitual MPA and/or MVPA *and* VPA with bone outcomes in children and adolescents (≤18 years). Thirty articles were included for review. Chi‐square tests were used to determine which PA intensity (MPA, MVPA, or VPA) had the greatest proportion of “statistically significant associations” with bone outcomes and which intensity had the greatest proportion of “strongest within‐study associations.”

**Results:** There was considerable heterogeneity in the accelerometry methods used. Studies varied in terms of the monitor make and model, wear criteria, epoch length, and cut‐point definitions of activity intensities. Regardless of the accelerometry methods employed, results were still indicative of a greater benefit of VPA over MPA/MVPA to bone outcomes. Of the 570 association analyses, 186 were significant (*p* < 0.05). Of these significant associations, chi‐square tests demonstrated that the proportion of strongest within‐study associations differed by PA intensity (3 × 2 χ^2^ = 86.6, *p* < 0.001) and was significantly higher for VPA (90/228) than for MVPA (8/151, 2 × 2 χ^2^ = 55.3, *p* < 0.001) and MPA (18/191, 2 × 2 χ^2^ = 49.1, *p* < 0.001).

**Conclusion:** Findings from this systematic review indicate that accelerometer‐derived VPA is more beneficial to bone outcomes compared to MPA or MVPA in children and adolescents aged ≤18 years. However, the widely varying accelerometry methods used to obtain PA data prevent the precise, beneficial amount of VPA from being identified. The frequent use of long epoch lengths (most commonly 60 seconds and 15 seconds) and numerous different intensity cut‐point definitions make it likely that bone‐relevant PA has been misrepresented in this population. The use of shorter epoch lengths (eg, 1 second) and development of bone‐specific activity intensity thresholds should be explored in the future.

## Other Biology

## Subchondral vascular modifications for hydraulic pressure load transmission

### Michael Beverly, David W Murray

#### *OOEC* *, Botnar Research Centre,* *NDORMS* *, Oxford* *OX3 7LD* *, Oxford, UK*

**Abstract**

**Introduction:** (i) We previously showed that intraosseous pressure (IOP) at rest is not a constant but is variable and proportional to local perfusion pressures at the needle tip. With activity there are significant pressure fluctuations of several atmospheres above arterial pressure with ordinary weight bearing. (ii) We previously described hypodense marks on upper tibial magnetic resonance imaging (MRI) scans which appear to be vascular, and which are reduced in number in early osteoarthritis. (iii) We note that bone fat is essentially liquid at body temperature. It flows from fractures and sprays from orthopedic saws or drills. No previous literature describes or explains this. (iv) Load appears to be transferred partly by hydraulic pressure acting through soft lipid and vascular tissues to transferring pressure on to the trabeculae and so to the cortical shaft. In this preliminary qualitative study, we sought histological evidence for those subchondral vascular marks on MRI and other subchondral structures that might allow and support hydraulic pressure load transfer during weight bearing.

**Method**: We examined axial or transverse plane histology for vessel morphology that might confirm that the MRI marks were vascular. We also looked for features that might represent a mechanism for controlling flow in and out of the subchondral region when pressurized. Normal human upper tibial bone was used (*n* = 6).

**Results:** Radiating vessels running parallel to the articular surface in the subchondral plane were found similar to those seen on MRI scans. The vessels penetrate the cortex near the joint margin. There are complex distortions at that point.

**Conclusion:** We identified previously undescribed vessels running in the subchondral plane consistent with marks seen on MRI scans. As they reach the cortical margin, complex distortions exist which may be choke valves that shut off to prevent high‐pressure flow out from the cancellous interior. Such a mechanism would prevent turbulent flow in fine capillaries and around delicate adipocytes. Arthritic bone had none of the longitudinal subchondral vessels or the subcortical choke valve morphological features. We suggest that there is a possible vasculo‐mechanical basis for osteoarthritis.

**Figure:**
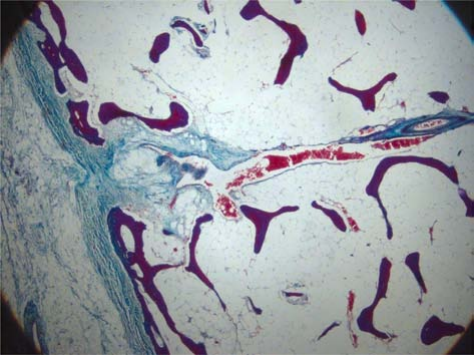
Figures 1 Human upper tibial sections cut in the subchondral plane. Cortex to the left. The vessel bundle is seen to approach the cortex but kink or distort immediately before entering the cortex. The appearances may represent a choke or valve or similar device. Masson's trichrome at x 2 magnification.

## Rare Conditions

## Measuring outcomes in ultra‐rare bone diseases: methodology of the palovarotene fibrodysplasia ossificans progressiva (FOP) clinical development program

### Robert J Pignolo^1^, Geneviève Baujat^2^, Matthew A Brown^3^, Carmen De Cunto^4^, Maja Di Rocco^5^, Edward C Hsiao^6^, Richard Keen ^7^, Mona Al Mukaddam^8^, Andrew Strahs^9^, Donna R Grogan^9^, Rose Marino^9^, Euvian Tan^10^, Mario Ippolito^10^, Manjinder Bains^10^, Frederick S Kaplan^8^

#### ^*1*^ *Department* *of Medicine, Mayo Clinic, Rochester,* *MN* *,* *USA* *.* ^*2*^ *Département* *de Génétique, Institut* *IMAGINE* *and Hôpital* *Necker‐Enfants* *Malades, Paris, France.* ^*3*^ *Guy* *'s & St. Thomas'* *NHS* *Foundation Trust and King′s College London* *NIHR* *Biomedical Research Centre, London, UK.* ^*4*^ *Pediatric* *Rheumatology Section, Department of Pediatrics, Hospital Italiano de Buenos Aires, Buenos Aires, Argentina.* ^*5*^ *Unit* *of Rare Diseases, Department of Pediatrics, Giannina Gaslini Institute, Genoa, Italy.* ^*6*^ *Division* *of Endocrinology and Metabolism,* *UCSF* *Metabolic Bone Clinic, Institute of Human Genetics, and* *UCSF* *Program in Craniofacial Biology, Department of Medicine, University of* *California‐San* *Francisco, San Francisco,* *CA* *,* *USA* *.* ^*7*^ *Centre* *for Metabolic Bone Disease, Royal National Orthopaedic Hospital, Stanmore, UK.* ^*8*^ *Departments* *of Orthopaedic Surgery & Medicine, The Center for Research in* *FOP* *and Related Disorders, Perelman School of Medicine, University of Pennsylvania, Philadelphia,* *PA* *,* *USA* *.* ^*9*^ *Ipsen* *, Newton,* *MA* *, USA.* ^*10*^ *Ipsen* *, Slough, UK*

**Abstract**

**Background:** Fibrodysplasia ossificans progressiva (FOP) is an ultra‐rare genetic disorder characterized by heterotopic ossification (HO) of soft and connective tissues (often preceded by flare‐ups), cumulative disability, and early mortality. Palovarotene (PVO) is a selective retinoic acid receptor gamma agonist under investigation for the treatment of FOP. Conducting trials in FOP has multiple challenges, including the low number of confirmed cases worldwide, a limited understanding of disease progression, and the need to identify disease‐specific biomarkers and optimize assessment of HO progression.

**Objective:** To develop a methodological approach addressing the challenges of conducting clinical trials in FOP.

**Methods:** Here, we describe the methodology of: a non‐interventional, prospective, protocol‐specified, longitudinal natural history study (NHS; NCT02322255); a multicenter, randomized, double‐blind, placebo‐controlled phase II trial (NCT02190747); and an ongoing open‐label extension (OLE) to the phase II trial (NCT02279095) (Table). Studies were designed adaptively. PVO doses were administered episodically (high dose for 2 or 4 weeks, followed by low dose for 4 or ≥8 weeks, from flare‐up onset) or chronically (daily). In the double‐blind period of the phase II trial, participants were randomized in two cohorts: 0:3:1 (aged ≥15 years) or 3:3:2 (≥6 years) to PVO 5/2.5 mg, PVO 10/5 mg or placebo, episodically for 2 weeks (high dose) then 4 weeks (low dose). HO incidence and volume are assessed annually by standardized low dose whole‐body computed tomography and/or at 12 weeks during the course of flare‐ups (defined as ≥2 [≥1 in OLE Part C] of pain, swelling, stiffness, decreased range of motion, redness, or warmth). Other clinical, functional, and patient‐reported outcomes are assessed using FOP‐specific measures of physical function, including the Cumulative Analogue Joint Involvement Scale (CAJIS) and FOP Physical Function Questionnaire (FOP‐PFQ). Studies were approved by independent ethics committees. A total of 151 unique participants were enrolled. Learnings informed the design of the phase III MOVE trial (NCT03312634).

**Conclusions:** Novel methodological approaches are needed to develop disease‐modifying treatments for serious, ultra‐rare diseases such as FOP. HO is the main cause of disability in patients with FOP. This program could be used as an example to inform the development of new treatments in rare bone diseases.

**Table:** **Table:** Clinical development program of PVO in FOP

	NHS	Phase II trial	Phase II OLE			
			Part A	Part B		Part C
	*n* = 114	*n* = 40	*n* = 40	*n* = 54		*n* = 48
			[a]	[b]		[c]
Patient age/skeletal maturity	0–65 years	≥6 years	≥6 years	Skeletally immature [d]	Skeletally mature [e]	≥6 years
PVO dosing regimen	N/A	Episodic (2/4 weeks)	Episodic (2/4 weeks)	Episodic (≥4/8 weeks)	Chronic (daily) + Episodic (≥4/8 weeks)	
PVO dose (mg)	None	5/2.5; 10/5; PBO	10/5	20/10	Chronic 5 + Episodic 20/10	

PVO doses were weight‐adjusted in skeletally immature children; [a] Including all 40 pts from the phase II trial; [b] Including 36 pts from OLE Part A and 18 new pts; 52 pts received PVO; [c] 48 pts from OLE Part B; [d] <90% skeletal maturity on hand/wrist radiography; [e] ≥90% skeletal maturity on hand/wrist radiography. FOP: fibrodysplasia ossificans progressiva; NHS: natural history study; OLE: open‐label extension; PBO: placebo; pts: participants; PVO: palovarotene.

## Femoral anteversion (FNA), and other lower limb geometry parameters in individuals with X‐linked hypophosphatemia (XLH)

### Matteo Scorcelletti ^1^, Serhan Kara^2^, Med. Lothar Seefried^3^, Jochen Zange^2^, Jörn Rittweger^2^, Alex Ireland^1^

#### ^*1*^ *Manchester* *Metropolitan University, Manchester, UK.* ^*2*^ *Institute* *of Aerospace Medicine* *DLR* *, Cologne, Germany.* ^*3*^ *University* *of Würzburg, Würzburg, Germany*

**Abstract**

**Background/Introduction:** X‐linked hypophosphatemia (XLH) is a rare genetic condition that affects phosphate metabolism, resulting in osteomalacia. Individuals with XLH are also at risk of lower limb deformities and early onset of hip osteoarthritis. These two factors may be linked, because abnormal femoral anteversion (FNA) (femoral torsion) is a risk factor for hip osteoarthritis. The contributions of regional femoral torsion, eg, intertrochanteric torsion (ITT), shaft torsion (ST), and condylar torsion (CT) to FNA differ between clinical groups and are important when planning femoral osteotomies to correct FNA. Other lower limb deformities such as bowing of femur and tibia and lower limb alignment have to be considered as well.

**Purpose:** This study aimed to compare total and regional femoral torsion, lateral and frontal bowing of the femur and tibia, and limb alignment between adults with XLH and controls.

**Methods:** Thirteen individuals with XLH (five male, age 49 ± 9 years) and 12 age‐, sex‐, and weight‐matched control participants (seven male, age 49 ± 8 years) were recruited following ethical approval and informed consent. Magnetic resonance imaging (MRI) scans of the femur were obtained, from which total and regional femoral torsion, lateral and frontal bowing of the femur and tibia, and limb alignment were measured. Data were normally distributed; therefore, group differences were assessed using *t* tests.

**Results:** FNA was 29 degrees lower in individuals with XLH than controls (*p* < 0.005). This resulted mainly from lower ITT (*p* < 0.001) and in part CT (*p* < 0.05) whereas ST was similar in the two groups (Fig. 1). Femoral lateral bowing was higher in individuals with XLH (13.1 ± 7.0 degrees) than controls (−1.0 ± 2.5 degrees, *p* < 0.001), as was femoral frontal bowing (31.4 ± 7.3 degrees in XLH, 17.8 ± 1.4 degrees in controls; *p* < 0.001). There was a 2.9‐degree difference in the mechanical axis between the XLH group (5.6 ± 5.3 degrees) and the control group (1.5 ± 2.5 degrees, *p* < 0.05).

**Conclusion(s):** Adults with XLH have substantial differences and greater interindividual variation in lower limb bone geometry, principally lower femoral torsion originating in the intertrochanteric region, and higher lateral and frontal femoral bowing, whereas differences detected between the groups in the mechanical axis and tibiofemoral angle are minimal. These differences are likely to be due to the higher malleability of the bones, variations in gate, delayed motor development, and impaired muscle function. The analyzed parameters may contribute to clinical problems such as hip osteoarthritis common in XLH. Information on region‐specific differences may be useful in planning corrective surgeries.

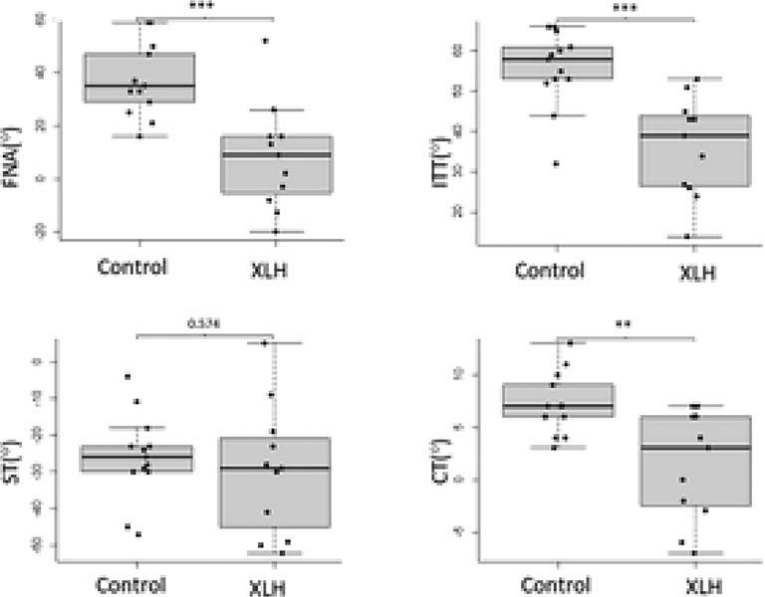

## Estimated prevalence of adults with X‐linked hypophosphatemia (XLH) in England based on burosumab early access experience from five sites

### Richard Keen ^1^, Judith Bubbear^1^, Gavin Clunie^2^, M Kassim Javaid^3^, Robin H Lachmann^4^, Elaine Murphy^4^, Matthew Roy^5^, Marian Schini^6^

#### ^*1*^ *Royal* *National Orthopaedic Hospital, Stanmore, UK.* ^*2*^ *Cambridge* *University Hospitals* *NHS* *Foundation Trust, Cambridge, UK.* ^*3*^ *University* *of Oxford, Oxford, UK.* ^*4*^ *National* *Hospital for Neurology and Neurosurgery, London, UK.* ^*5*^ *Bristol* *Royal Infirmary, Bristol, UK.* ^*6*^ *University* *of Sheffield, Sheffield, UK*

**Abstract**

Adults with X‐linked hypophosphatemia (XLH) experience a significant burden, living with a lifelong, progressive and debilitating condition.

Burosumab, is an European Medicines Agency (EMA)‐approved treatment for XLH that addresses the pathophysiology of the disease (fibroblast growth factor 23 [FGF23]‐induced hypophosphatemia) and has been trialed in symptomatic adults with the condition. Based on the latest evidence for UK prevalence using routine primary care records, the XLH population in England ranges from 291 to 578 adults. Uncertainty remains on the number of patients affected by debilitating symptoms and clinical complications. According to clinical practice not all patients will be eligible for burosumab, and the lower range is most applicable to the likely treatment population.

We used data from an early access program (EAP) to inform an estimate of the number of adults with XLH, and thus the number with debilitating symptoms who might be eligible for burosumab as per the EAP criteria.

A survey was conducted to gather information on the adult XLH population across five centers in England participating in the burosumab EAP. Each specialist unit was asked to provide the number of adult XLH patients known to their service, the number receiving burosumab through the EAP, and the total catchment population served by their unit. Where the adult XLH population was unknown or uncertain, an estimate was made using the observed ratio of burosumab to total adult XLH patient numbers based on data from other centers. Data from the EAP centers were then compared with expected XLH prevalence data, using published Office for National Statistics (ONS) population figures and estimated published prevalence for adult XLH.

Across the five sites, of 180 adult XLH patients known to the centers, 86 (47.8%) patients received burosumab through the EAP to date. The five centers are known to serve a combined catchment population of approximately 26 million adults (60% of England's total adult population). Assuming the number of patients known to the EAP centers is accurate, this would indicate a total of 305 adults with XLH for the whole of England, with approximately 152 patients eligible for burosumab based on the severity of symptoms. With a 10% inflation, the upper expected number of adults eligible for burosumab would be 167.

Using experience from five specialist units who regularly manage adults with XLH, and are engaged in an EAP, it is estimated that there are 305 adults with XLH presenting to the National Health Service (NHS) across England, of which 152 may be eligible for treatment with burosumab.

### Peer Review

The peer review history for this article is available at https://publons.com/publon/10.1002/jbm4.10552.

